# Comparative metabolomics of single-strain and co-cultures of seven *Fusarium* strains causing *Fusarium* head blight reveals metabolic modifications induced by strain co-occurrence

**DOI:** 10.1016/j.crmicr.2026.100657

**Published:** 2026-08-04

**Authors:** Valentin Fiévet, Laëtitia Pinson-Gadais, Marie-Noëlle Verdal-Bonnin, Maxime Maurin, Stéphane Bernillon, Louis Carles, Florence Richard-Forget

**Affiliations:** INRAE, MycSA, 33883, Villenave d’Ornon, France

**Keywords:** *Fusarium* Head Blight, Endometabolome, Exometabolome, Metabolic biomarker, Microbial interactions

## Abstract

*Fusarium* Head Blight (FHB) is a devastating fungal disease affecting cereal crops, mainly caused by *Fusarium graminearum, Fusarium culmorum, Fusarium poae, Fusarium avenaceum, Fusarium langsethiae, Fusarium sporotrichioides,* and *Fusarium tricinctum*. In addition to inducing yield losses, the previous species are responsible for grain contamination with mycotoxins. Co-occurrence of these species is frequent and driven by fungal interactions that influence disease outcomes. To investigate those interactions, intra- and extracellular metabolomic profiles of one representative strain per FHB species were compared. In total, 932 intracellular and 1170 extracellular metabolites were detected. Strains from *F. graminearum, F. culmorum*, F*. sporotrichioides*, and *F. tricinctum* exhibited the highest number of biomarkers (141, 79, 69, and 58, respectively), whereas fewer than 15 biomarkers were detected for strains of *F. avenaceum, F. poae, and F. langsethiae*. Tracking these biomarkers in *in vitro* co-cultures revealed that only those associated with the most abundant strains, *F. culmorum* followed by *F. graminearum*, were consistently detected. Comparison of biomarker dynamics between single-strain and co-cultures revealed significant differences in accumulation profiles and concentrations, with some biomarkers showing abundance differences of up to 70-fold. This allowed us to raise several hypotheses, including up- and down-accumulation of some biosynthetic pathways, in addition to detoxification, biotransformation, or cross-feeding mechanisms induced by fungal interactions. This work establishes a first conceptual and methodological framework to study *Fusarium* interactions through metabolomic profiling of *Fusarium* co-cultures. It aimed to improve our understanding of the FHB disease process to ultimately mitigate its impact.

## Highligths

• Metabolomics revealed a core-metabolome shared by 7 FHB-associated strains.
• Strain biomarkers were identified for 7 FHB-associated strains.
• Metabolomic clustering of FHB-associated strains partially reflected phylogeny.
• Tracking strain biomarkers revealed metabolic shifts induced by co-cultivation.

## Graphical abstract

**Figure:**
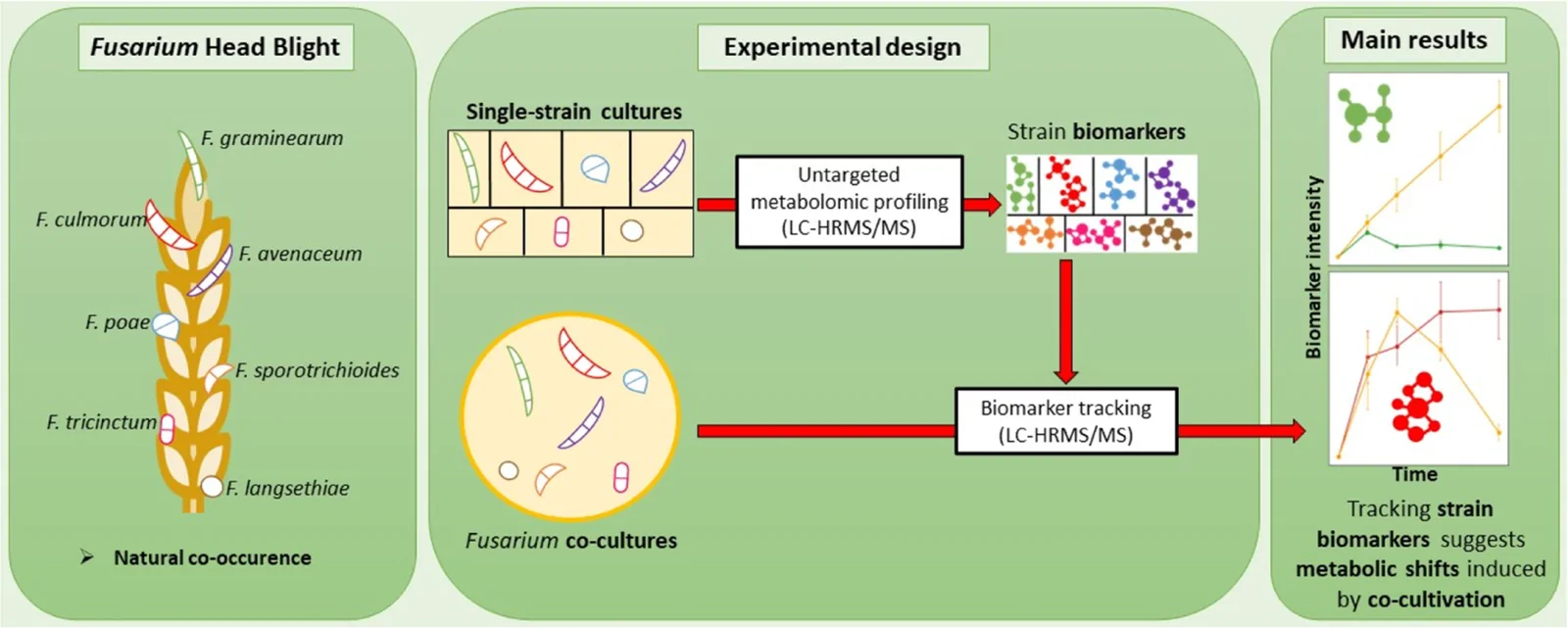

## 1 Introduction

*Fusarium* Head Blight (FHB) is one of the most destructive fungal diseases worldwide, causing severe yield losses in small-grain cereals as well as maize (Karlsson, 2015; Parry et al., 1995; Xu and Nicholson, 2009). More than 20 *Fusarium* species were reported as associated with FHB across the world (Becher et al., 2013; Karlsson et al., 2021; Valverde-Bogantes et al., 2020; Xu and Nicholson, 2009). The genus *Fusarium* is currently divided into 23 informal multispecies lineages, termed species complexes (Geiser et al., 2021). In Europe, the majority of FHB pathogens belong to the *Fusarium sambucinum* species complex (FSAMSC). These pathogens include *Fusarium graminearum sensu stricto* (referred to hereafter as *F. graminearum*), *Fusarium culmorum, Fusarium poae, Fusarium langsethiae*, and *Fusarium sporotrichioides*. In addition, members of the *Fusarium tricinctum* species complex (FTSC), such as *Fusarium avenaceum* and *Fusarium tricinctum*, are also prevalent in Europe (Bottalico and Perrone, 2002; Johns et al., 2022; Karlsson et al., 2021; Xu et al., 2005; Xu and Nicholson, 2009). Beyond yield losses, FHB is responsible for the contamination of grain with mycotoxins, which are fungal specialized metabolites posing serious food safety and health concerns (Bottalico and Perrone, 2002; Leslie et al., 2021; Mielniczuk and Skwaryło-Bednarz, 2020; Miller, 2008). *Fusarium* species are known to produce a wide array of mycotoxins which encompass minor and major mycotoxins, with the concentration of most of them being regulated in crops and derived products by the European Union (EU) Regulation 2024/1022 (European Commission, 2024). *Fusarium* mycotoxins in cereal-derived products can pose a serious risk to human health, due to their toxicity, even at low concentrations (Azam et al., 2021). Major mycotoxins associated with FHB include type A trichothecenes (TCTA), whose main representatives are T-2 toxin, HT-2 toxin, and diacetoxyscirpenol (DAS), mainly produced by *F. sporotrichioides* and *F. langsethiae* (Alisaac and Mahlein, 2023; Munkvold, 2017)*.* Type B trichothecenes (TCTB) are EU-regulated and encompass deoxynivalenol (DON) and its acetyl derivatives, 3- and 15-acetyldeoxynivalenol (3- and 15-ADON), nivalenol (NIV), and fusarenon-X (FX), mainly produced by *F. culmorum* and *F. graminearum*. The two former species can also produce the mycotoxin zearalenone (ZEN). Regarding *F. poae,* this species can synthesize both TCTA (DAS) and TCTB (NIV/FX) (Alisaac and Mahlein, 2023; Munkvold, 2017). In addition, a group of “emerging” mycotoxins, which are not routinely monitored and regulated, is associated with FHB. This group includes beauvericin (BEA) and enniatins (ENNs), particularly the A, A1, B, and B1 analogs, produced primarily by *F. avenaceum, F. tricinctum,* and to a lesser extent *F. poae* (Alisaac and Mahlein, 2023; Munkvold, 2017).

In addition to mycotoxins, *Fusarium* species are capable of producing a wide diversity of specialized metabolites (Amuzu et al., 2024). Genes encoding biosynthetic pathways of specialized metabolites are commonly co-localized in biosynthetic gene clusters (BGCs). These clusters typically include genes coding for a backbone enzyme that defines the chemical class—polyketide synthases (PKSs), non-ribosomal peptide synthetases (NRPSs), and terpene synthases/cyclases (TSs/TCs)—along with genes coding for tailoring enzymes responsible for chemical structure modifications, for cluster-specific transcription factors, and sometimes self-protection genes (Chiang et al., 2010; Eagan and Keller, 2025; Hur et al., 2012; Keller, 2015; Schmidt-Dannert, 2015). Diversity of metabolites produced among *Fusarium* species has been mainly investigated using genomic approaches (Amuzu et al., 2024; Li et al., 2020; Toghueo, 2020; Wei and Wu, 2020). Genome mining revealed that *Fusarium* genomes typically harbor an average of 50 BGCs, ranging from 38 to 65 depending on the species (Hoogendoorn et al., 2018; Lin et al., 2023). Lin et al. (2023) demonstrated that the predicted BGC type distribution is correlated with phylogenetic lineage, with NRPS BGCs being the most commonly encountered across *Fusarium* species (Lin et al., 2023). While some BGCs are widely conserved across pathogenic species (*e.g.*, BGCs for gibepyrones, fusarubins, bikavericins) (Lin et al., 2023), other BGCs appear to be lineage-specific or even species-specific (Hoogendoorn et al., 2018). Among FHB-associated species, *F. tricinctum* and *F. avenaceum* harbor the largest number of predicted BGCs, approximately 60 BGCs per genome, including a high percentage of NRPS clusters (Lin et al., 2023). *F. culmorum, F. graminearum,* and *F. poae* showed similar BGC richness, with a number of predicted BGCs ranging from 41 to 48 (Lin et al., 2023). Regarding the other *Fusarium* species, data related to *F. langsethiae* were published by Lysøe et al. (2016), who documented 47 genes coding for backbone enzymes, the most prevalent being TSs (22 predicted), followed by NRPS (13 predicted) and PKS (12 predicted) (Lysøe et al., 2016). To the best of our knowledge, no comprehensive studies addressing BGCs prediction in the *F. sporotrichioides* genome have been published so far. However, many of the BGC clusters may remain silent under standard laboratory conditions, complicating their functional characterization (Zhgun, 2023). Hence, while genomic approaches are useful for characterizing an overall biosynthetic potential, they need to be combined with other strategies, such as metabolomics, to identify specialized metabolites that are actually produced in a defined environment. Therefore, genomic predictions alone are insufficient to understand the metabolites effectively produced under ecologically relevant conditions.

By combining, both genomic and metabolomic approaches, Niehaus et al. (2016) compared species within the *Fusarium fujikuroi* species complex (*e.g., Fusarium proliferatum, Fusarium verticillioides, Fusarium mangiferae, Fusarium oxysporum*), revealing significant interspecific differences in specialized metabolite gene clusters and corresponding metabolite production profiles (Niehaus et al., 2016). Regarding FHB-associated species, the assessment of metabolic diversity has been hitherto mainly focused on known mycotoxins and virulence factors (Beccari et al., 2020, 2018; Crippin et al., 2019; Thrane et al., 2004; Witte et al., 2024). There is currently a notable lack of comparative studies using untargeted metabolomics to analyze the variability of metabolic profiles between *Fusarium* species associated with FHB.

In the field, co-occurrence of *Fusarium* species causing FHB is a common situation (Doohan et al., 2003; Karlsson et al., 2021; Parikka et al., 2012; Xu et al., 2008; Xu and Nicholson, 2009). Such co-occurrences are driven by fungal interactions, that, *in fine*, strongly affect disease outcomes, including disease symptoms and mycotoxin contamination (Xu and Nicholson, 2009; Karlsson et al., 2021; Ferrigo et al., 2016; Wang et al., 2023). Yet the principles governing these interactions and their consequences on specialized metabolism remain poorly understood. Even for mycotoxins, their ecological roles remain largely unresolved. Some hypotheses suggest that they participate in plant–fungus interactions, confer competitive advantages within microbial communities, serve as defense compounds against predators, and contribute to complex stress-response systems. (Eagan and Keller, 2025; Perincherry et al., 2019; Ponts, 2015; Venkatesh and Keller, 2019). It is now well established that microbial interactions affect BGCs expression, and conversely, that specialized metabolites are central mediators of these interactions (Adejor et al., 2025; Eagan and Keller, 2025; Zakariyah et al., 2024; Zhgun, 2023). For instance, Chevrette et al. (2022) demonstrated that specialized metabolism dynamics of a simplified model of the rhizosphere composed of *Bacillus cereus, Flavobacterium johnsoniae*, and *Pseudomonas koreensis* were influenced by community composition, with BGC transcript abundance and metabolite features differing among single-strain cultures, dual co-cultures, and a tripartite bacterial community (Chevrette et al., 2022). Similarly, co-cultivation of *Aspergillus nidulans* with other filamentous fungi was shown to activate multiple silent BGCs and, consequently, to broadly modify the profile of yielded specialized metabolites (Wang et al., 2022). However, how interspecific interactions among FHB-associated species shape their specialized metabolomes remains largely unexplored.

The present study aims to support the promising use of metabolomics to provide new insights regarding both the diversity of *Fusarium* specialized metabolites and investigate the impact of fungal interactions involved in the FHB disease. We hypothesized that (i) FHB-associated species share a common metabolic backbone but display distinct species-specific metabolomic signatures and (ii) co-cultivation alters these metabolic profiles, revealing interaction-driven modulation of specialized metabolism. To test these hypotheses, the metabolomic profiles of *Fusarium* strains, from seven major species causing FHB (*F. graminearum, F. culmorum, F. poae, F. sporotrichioides, F. langsethiae, F. avenaceum*, and *F. tricinctum*) were compared, and strain-specific metabolites (*i.e.*, biomarkers) were identified. Monitoring the behavior of these biomarkers in single-strain cultures and within co-cultures was implemented to demonstrate the impact of *Fusarium* strains co-occurrence on individual metabolism. This study provides the first untargeted comparative metabolomic analysis of seven FHB-associated *Fusarium* strains. Based on a restricted set of *Fusarium* strains (one per species), the present study does not aim to decipher FHB interspecies interactions but to establish a methodological framework for investigating how such interactions could shape fungal specialized metabolism, opening the way towards an increased understanding of the community-level processes underlying FHB.

## 2 Materials and methods

### 2.1 Biological material

Seven *Fusarium* strains (one strain per species; *F. graminearum, F. culmorum, F. sporotrichioides, F. langsethiae, F. poae, F. avenaceum*, and *F. tricinctum*) were used in this study. These strains, together with information relative to their origin and capacity to produce mycotoxins, are summarized in Table 1. The strains from INRAE-Bordeaux MycSA collection are deposited in the International Center for Microbial Resources—Filamentous Fungi (CIRM-CF, https://www.cirm-fungi.fr/, Marseille, France).

**Table 1:** Seven strains of seven different *Fusarium* species were used.

Species	Strain	Supplier Identifier	Origin	Chemotype
*F. avenaceum*	Fav498	129	Bayer, France	ENNs, BEA
*F. culmorum*	Fcu337	INRA 337	MycSA collection, France	NIV/FX, ZEN
*F. graminearum*	Fgr149	INRA 149	MycSA collection, France	DON/15-ADON, ZEN
*F. langsethiae*	Fla509	117 (7CNJMB21)	Bayer, France	T2, HT2, DAS
*F. poae*	Fpo073	CBS 317.73	Westerdijk Fungalbio Diversity Institute, The Netherlands	DAS, NIV/FX, ENNs, BEA
*F. sporotrichioides*	Fsp101	INRA 101	MycSA collection, France	T2, HT2, DAS
*F. tricinctum*	Ftr521	1 (7CNAG17)	Bayer, France	ENNs, BEA

15-ADON: 15-acetyldeoxynivalenol; BEA: Beauvericin; DAS: diacetoxyscirpenol; DON: deoxynivalenol; ENNs: enniatins; FX: fusarenon-X; HT2: HT-2 toxin; T2: T-2 toxin; ZEN: Zearalenone.

### 2.2 Spore production

Fungal cultures were maintained at 4 °C on Potato Dextrose Agar (PDA; BD Difco, Franklin Lakes, NJ, USA). When inoculum was required, the fungal strain was grown on PDA plates at 25 °C in the dark for 5 days. Spore suspensions were prepared with four plugs of fungus in 20 mL of carboxymethyl cellulose (CMC) liquid medium (15 g CMC, 1 g yeast extract, 0.5 g MgSO_4_ 7H_2_0, 1 g NH_4_NO_3_, 1 g KH_2_PO_4_, for 1 L medium) and incubated in the dark at 25 °C and at 180 rpm in a Multitron incubator shaker (Infors HT, Bottmingen, Switzerland). After 2 to 5 days, conidia were harvested by filtration through a 40 μm cell strainer (Biologix *G*roup Limited, Jinan, China) and recovered using centrifugation (4800 × g for 10 min). Supernatants were discarded, and spore pellets were resuspended in 2 mL of sterile distilled water. Spore concentration was determined using a Malassez counting chamber and diluted with sterile standardized sucrose synthetic medium (MS: 20 *g*·L⁻¹ sucrose, 0.5 *g*·L⁻¹ KH₂PO₄, 0.6 *g*·L⁻¹ K₂HPO₄, 0.017 *g*·L⁻¹ MgSO₄, 1 *g*·L⁻¹ (NH₄)₂SO₄, and 0.1 mL·L⁻¹ Vogel mineral salts solution) to reach a concentration of 8 × 10⁵ spores·mL⁻¹ for single-strain cultures experiment. For the co-cultures experiment, equal volumes of spore suspension of each strain were combined, corresponding to a final concentration of 1.14 × 10⁵ spores·mL⁻¹ per strain, with a total spore concentration of 8 × 10⁵ spores·mL⁻¹.

### 2.3 Inoculation and sampling

Liquid cultures were performed in 55 mm Petri dishes containing 8 mL of a standardized sucrose synthetic medium (MS) (Merhej et al., 2010). For the single-strain cultures experiment, 100 µL of spore suspension at 8 × 10⁵ spores·mL⁻¹ was inoculated in 8 mL of MS to reach a final concentration of 1 × 10⁴ spores·mL⁻¹. Similarly, for the co-cultures experiment, 100 µL of the mixed spore suspension was added to reach 1.42 × 10³ spores·mL⁻¹ per strain, totaling 1 × 10⁴ spores·mL⁻¹. Cultures were incubated at 25 °C in the dark for 14 days. Samples were collected at 3, 5, 7, 10, and 14 days post-inoculation. Mycelium was scraped with a sterile spatula, and cultures were filtered through a 100 μm cell strainer (Biologix Group Limited, Jinan, China) to separate the medium and mycelium. For untargeted metabolomics analysis, 1 mL of culture medium was filtered through a filter syringe (Acrodisc 13 mm mini spike with 0.2 μm wwPTFE; Pall Corporation, Port Washington, NY, USA), immediately frozen in liquid nitrogen to quench metabolism, and stored at −80 °C. Mycelium was rinsed with 5 mL of sterile distilled water, frozen in liquid nitrogen, and stored at −80 °C for biomass and metabolomic analysis. Additionally, mycelium samples from co-cultures were collected for biomass measurement and DNA extraction and stored at −80 °C. Freeze-drying of mycelium was performed using a Pilote Compact freeze-drier (Cryotec, Saint-Gély-du-Fesc, France). The process consisted of three successive phases: freezing, primary drying, and secondary drying. Samples were first frozen on the shelves by decreasing the temperature to −25 °C and maintaining this temperature for 30 min to ensure complete solidification of the material. After freezing, primary drying was initiated under reduced pressure. A chamber pressure of approximately 0.402 mbar was applied, with heating starting once the vacuum reached 2.023 mbar. During primary drying, the shelf temperature was progressively increased in a stepwise program with controlled ramps of 0.5 °C min⁻¹. The shelves were sequentially stabilized at −25 °C (9 h), −10 °C (9 h), 0 °C (9 h), and 10 °C (9 h), followed by a final increase to 20 °C with a short stabilization period (20 min). At the end of primary drying, the temperature was further increased to 25 °C and maintained for 120 min to complete water sublimation. Then, fungal biomass was determined by weighing freeze-dried mycelium.

### 2.4 Untargeted metabolomic profile analysis

#### 2.4.1 Sample preparation and metabolites extraction

For mycelium, 10 mg (± 0.5 mg) of freeze-dried material was mixed with 1 mL of methanol:water (70:30, v/v) containing 0.1 % formic acid and methyl vanillate (Sigma-Aldrich, St. Louis, MO, USA) as an internal standard at a final concentration of 1.37 mM. Samples were sonicated in a melting ice bath for 15 min, centrifuged at 14,500 × g for 5 min at 4 °C, and filtered using a filter syringe (Acrodisc 13 mm mini spike with 0.2 μm wwPTFE, Pall Corporation, Port Washington, NY, USA) into HPLC vials. For culture medium, 500 µL of medium was mixed with 500 µL of methanol:water (1:1, v/v) containing 2.74 mM methyl vanillate, resulting in a final concentration of 1.37 mM. Samples were filtered as described above and stored at −20 °C until analysis by LC–HRMS with tandem MS (LC–HRMS/MS).

#### 2.4.2 LC-HRMS/MS analysis of polar and semi-polar metabolites

Metabolites were separated on a Hypersil GOLD aQ C18 column (100 × 2.1 mm, 1.9 μm; Thermo Fisher Scientific, Waltham, MA, USA) using a Vanquish UHPLC system (Thermo Fisher Scientific, Waltham, MA, USA). Solvent A was 0.1 % formic acid in water, and solvent B was 0.1 % formic acid in acetonitrile. The gradient was: 0–1.4 min, 5 % B; 1.4–4.5 min, 5 % B; 4.5–11 min, 25 % B; 11–14 min, 70 % B; 14–17.5 min, 95 % B; 17.5–18 min, 95 % B; 18–23 min, 5 % B. The flow rate was set at 0.4 mL.min⁻¹, the column temperature at 35 °C, and the injection volume at 2 μL. Detection was performed using a Q Exactive Focus mass spectrometer (Thermo Fisher Scientific, Waltham, MA, USA) in positive electrospray ionization (ESI) mode. Full-scan acquisition (*m/z* 80–1100) was performed at a resolving power of 70,000. Data-dependent MS² (dd-MS²) acquisition was performed at 17,500 resolving power, with a 3.0 *m/z* isolation window and 2 s dynamic exclusion. Fragmentation was carried out in the HCD cell with 30 % normalized collision energy. Nitrogen was used as the sheath, auxiliary, and sweep gas at 50, 13, and 0 arbitrary units (AU), respectively. S-lens RF level was 50 %. Extraction blanks and medium-only blanks were analyzed throughout. A quality control (QC) sample, containing 10 µL of each sample, was injected every 10 sample injections. A pooled mix of mycotoxin standards was injected to support metabolite annotation and increase confidence in compound identification. The mix was composed of the Enniatins and Beauvericin mixture STD-ENABE-10 (Libios, Toulouse, France), composed of enniatins A, A1, B, B1, and beauvericin, and the Biopure mix 4 (Romer Labs, Tulln, Austria), composed of 3-acetyldeoxynivalenol, deoxynivalenol, nivalenol, fusarenon-X, HT-2 toxin, T-2 toxin, diacetoxyscirpenol, and zearalenone. Because 3-ADON and 15-ADON are isomers with identical mass and very similar chromatographic behavior, they cannot be distinguished under standard LC-MS conditions. Therefore, in the strain Fgr149 known to produce only 15-ADON (Table 1), the 3-ADON standard was used as a reference to identify 15-ADON based on retention time and fragmentation patterns.

#### 2.4.3 Metabolomic data processing

Raw data were processed using Compound Discoverer v3.3 (Thermo Fisher Scientific, Waltham, MA, USA) with an untargeted metabolomic workflow including chromatographic alignment (ChromAlign), feature detection and grouping, gap filling, background subtraction using blanks, and QC-based correction (SERRF). Putative metabolite annotation was done by combining accurate mass, elemental composition prediction, and spectral matching against mzCloud, ChemSpider, and custom mass lists. Candidate annotations were prioritized using mzLogic, and pathway mapping was performed using Metabolika. Single-strain cultures and co-cultures datasets were processed separately, and mycelium and culture medium samples were analyzed independently. Mycelium data were normalized to the extracted mycelial weight. Features identified as background, with a retention time inferior to 1.50 min, lacking MS² spectra, or with a peak rating inferior to 5, were excluded. Manual inspection of chromatographic peaks ensured data quality. The final features tables were exported for downstream analysis. Confidence level of identification (CLI), following the level described by Schymanski et al. (2014), was added to the features with an in-house Excel macro used on the metabolomic features tables returned by Compound Discoverer v3.3: Level 1 indicates confirmed structure via an authentic reference standard match (*i.e.*, MS, MS/MS, and retention time match); Level 2a indicates probable structure according to a full spectral matches in mzCloud; Level 2b (not used in this study) represents a diagnostic of the structure, evidenced via MS/MS fragments and/or ionization behavior, parent compound information and the experimental context; Level 3 represents tentative structures identified through a partial spectral match in mzCloud; Level 4 corresponds to compounds identified solely by predicted molecular formulas without any structural information (*i.e.*, no matches in mzCloud) and any associated compound names retrieved in non-spectral databases (*e.g.*, ChemSpider, Metabolika, in-house MassList of *Fusarium* metabolites) are speculative and should not be interpreted as reliable identifications; Level 5 applies to features having only a confirmed measured mass with a lack of information to assign or even predict a formula related to the mass or the matches in non-spectral databases, and, therefore, putative formula and name returned by Compound Discoverer v3.3, at this level, were removed.

### 2.5 Strain composition analysis

#### 2.5.1 DNA extraction

For *Fusarium* co-cultures, DNA was extracted from 10 mg (± 0.5 mg) of freeze-dried mycelium using the DNeasy Plant Mini Kit (Qiagen, Hilden, Germany) with minor modifications. Mycelium was ground with 0.5 g of 0.5 mm glass beads using a Precellys Evolution (Bertin Instruments, Montigny-le-Bretonneux, France) at 6800 rpm for 2 × 25 s with a 5 s pause. 400 µL of AP1 buffer was added, and samples were homogenized at 7500 rpm for 2 × 25 s with a 3 s pause. Tubes were centrifuged at 17,000 × g. RNase A (4 µL) was added, and samples were incubated at 65 °C for 1 h with mixing every 5 min. DNA was eluted with 50 µL elution buffer, and a second elution was performed using the same eluate. DNA quality was assessed using a DS-11 FX+ fluorometer–spectrophotometer (Denovix Inc., Wilmington, DE, USA), and quantity was measured using Qubit dsDNA BR Assay Kit (Invitrogen, Waltham, MA, USA). DNA was diluted to 10 ng·µL⁻¹ with nuclease-free water.

#### 2.5.2 Strain quantification by real-time PCR

Species-specific qPCR was adapted from Elbelt et al. (2018), targeting the *TEF1* gene (Elbelt et al., 2018). Primers and probes are listed in Table 2. Probes were synthesized by Integrated DNA Technologies (IDT, Coralville, IA, USA), labeled with FAM, and double-quenched with internal ZEN and 3′ Iowa Black® FQ. qPCR reactions (final volume of 10 µL) included 5 µL of Gene Expression Master Mix (IDT, Coralville, IA, USA), 0.3 µL of forward and reverse primers (10 µM each; IDT, Coralville, IA, USA), 0.1 µL of probe (10 µM), 1 µL of DNA (10 ng·µL⁻¹), and 3.3 µL of nuclease-free water. Experiments were performed using a QuantStudio 5 system (Thermo Fisher Scientific, Waltham, MA, USA). Thermal cycling conditions consisted of an initial denaturation at 95 °C for 3 min, followed by 40 cycles of 95 °C for 5 s, annealing at the species-specific temperature listed in Table 3 for 30 s, and 4 °C for 45 s. Reactions were performed in triplicate. Standard curves were generated using gBlocks *TEF1* gene fragments (IDT, Coralville, IA, USA) for each species (Table 3). DNA quantity was expressed as *TEF1* gene copies per ng of total DNA.

**Table 2:** Primer and probe sequences used for species-specific qPCR quantification of *Fusarium* spp., including reporter and quencher labels and probe annealing temperatures.

**Target species**	**Primer name**	**Primer sequence (5′-3′)**	**Probe name**	**Probe sequence (5′-3′)**
***F. avenaceum***	EF1-FA_F2	CATCTTGCTAACTCTTGACAGACCG	Probe_Fave_Ef1alpha-FAM	/56-FAM/ AGCGAGTCG/ZEN/TGGGAATCGATGGG/3IABkFQ/
	EF1-FA_R3	GGGTAATGAATGCGTTTCGAA		
***F. culmorum***	EF1-FC_F2	CGAATCGCCCTCACACG	Probe_Fculmo_Ef1alpha-FAM	/56-FAM/ ATGAGCCCC/ZEN/ACCAGAAAAATTACGACAA/3IABkFQ/
	EF1-FC-R2	GTGATGGTGCGCGCCTAG		
***F. graminearum***	EF1-FC FG_F	TCGATACGCGCCTGTTACC	Probe_Fgrami_Ef1alpha-FAM	/56-FAM/ AGCCCCACC/ZEN/GGGAAAAAAATTACGACA/3IABkFQ/
	EF1-FG_R	ATGAGCGCCCAGGGAATG		
***F. langsethiae***	EF1 FL F3	GCCGTGTCGTAATTTTTTTTGTG	Probe_Flang_Ef1alpha-FAM	/56-FAM/GGGCTCATA/ZEN/CCCCGCCACTCGA/3IABkFQ/
	EF1 FL R3	AAATGGCTATGTGGGAAGGAAG		
***F. poae***	EF1-FP2_F	CTCGAGCGATTGCATTTCTTT	Probe_Fpoae_Ef1alpha-FAM	/56-FAM/ CGCGAATCG/ZEN/TCACGTGTCAATCAGTT/3IABkFQ/
	EF1_FP2_R	GGCTTCCTATTGACAGGTGGTT		
***F. sporotrichioides***	EF1-FS_F3	GGCTCATACCCCGCCG	Probe_Fsporo_Ef1alpha-FAM	/56-FAM/ TGGGAAGGG/ZEN/CAAAAGCGCCTGT/3IABkFQ/
	EF1-FS_R2	GCGCCCATGTAAATGGATG		
***F. tricinctum***	EF1-FT_F2	CCACGATTCGCTCCCTCAC	Probe_Ftric_Ef1alpha-FAM	/56-FAM/ AGCGGGGTA/ZEN/ATGGATGCGTTTCGAGT/3IABkFQ/
	EF1-FT_R2	GGTAAGATACCCCACCAGAAAAA		

ZEN: internal ZEN quencher; 3IABkFQ 3′: 3′ Iowa Black®FQ quencher; 56-FAM: FAM reporter fluorophore.

**Table 3:** List of synthesized *TEF1* gene fragments used as species-specific DNA standards for the generation of qPCR calibration (standard) curves for each *Fusarium* strain.

***Species***	**Synthesized *TEF1* gene fragment (5′-3′)**
***F. avenaceum***	CTGGCGGGGTATCACCACGACATCTTGCTAACTCTTGACAGACCGGTCACTTGATCTACCAGTGCGGTGGTATCGACAAGCGAACCATCGAGAAGTTCGAGAAGGTTAGTCAATATCCCTTCGATTACGCGCGCTCCCATCGATTCCCACGACTCGCTCCCTCATTCGAAACGCATTCATTACCCCGCTCAAGTCCGAAAATTTT
***F. culmorum***	TTCCCTTTCGAAACATCATTCGAATCGCCCTCACACGACGACTCGATACGCGCCTGTTACCCCGCTCGAGGTCAAAAATTTTGCGGCTTTGTCGTAATTTTTCTGGTGGGGCTCATACCCCGCCACTCGAGCGACAGGCGCTTGCCCTCTTCCCACAAACCATTCCCTAGGCGCGCACCATCACGTGTCAATCAGTTACTAACC
***F. graminearum***	AATCGCCCTCACACGACGACTCGATACGCGCCTGTTACCCCGCTCGAGGTCAAAAATTTTGCGGCTTTGTCGTAATTTTTTTCCCGGTGGGGCTCATACCCCGCCACTCGAGCGACAGGCGTCTGCCCTCTTCCCACAAACCATTCCCTGGGCGCTCATCATCACGTGTCAACCAGTCAC
***F. langsethiae***	CGCTCGAGTTTAAAATTTTACGGCCGTGTCGTAATTTTTTTTGTGGTGGGGCTCATACCCCGCCACTCGAGTGACAGGCGCTCTTCCTTCCCACATAGCCATTTACATGGGCGCGCATCATCACGT
***F. poae***	TTTCGGTGGGGCTTATACCCCGCCACTCGAGCGATTGCATTTCTTTGGGCGCGAATCGTCACGTGTCAATCAGTTACTAACCACCTGTCAATAGGAAGCCGCCGAGCTCGGTAAGGGTTCTTTCA
***F. sporotrichioides***	ACGGCTGTGTCGTGATTTTTTGATAGTGGGGCTCATACCCCGCCGCTCGAGTGACAGGCGCTTTTGCCCTTCCCACACATCCATTTACATGGGCGCGCATCATCACGTGTCAATCAGTCACTAAC
***F. tricinctum***	CGCTCCCATCGATTCCCACGATTCGCTCCCTCACTCGAAACGCATCCATTACCCCGCTCGAGCCCGAAAATTTTGCGGTGCGACCGTGATTTTTTTTCTGGTGGGGTATCTTACCCCGCCACTCGAGTGA

### 2.6 Statistical analysis and results visualization

Statistical analyses and visualization of growth, metabolomic, and DNA quantification data were conducted using R v4.5.2 (R Foundation, Vienna, Austria) within the RStudio environment v2026.01.1 (Posit Software, PBC, Boston, MA, USA) (Posit Software, PBC, 2025; R Core Team, 2026). Metabolomic features were considered as detected in a sample when their abundance exceeded ten-fold the thresholds of noise for endometabolomic features (mycelia) or when they exceeded ten-fold the mean value of the control condition, *i.e.* without inoculum, for exometabolomic features (culture media). Shared features between mycelium and medium datasets were matched with a tolerance of 0.005 Da for calculated molecular weight and 0.033 min for retention time. Principal component analysis was performed with FactoMineR after log₂ transformation and autoscaling (Husson et al., 2025). Hierarchical clustering heatmaps were generated using ComplexHeatmap (Gu, 2025), where samples and features were clustered using Euclidean distance and the complete-linkage method. Sets of metabolites shared by all seven strains, by subsets of strains, or uniquely present in individual isolates were visualized with upset plots using the ComplexUpset package (Krassowski, 2021). Shared biomarkers between single-strain cultures and co-culture experiments were matched with a tolerance of 0.005 Da and 0.2 min, combined with MS² spectral comparison. Peak intensities were normalized to the internal standard (methyl vanillate) when comparing accumulation dynamics of biomarkers in single-strain cultures and co-culture experiments.

## 3 Results

### 3.1 Growth kinetics of the seven *Fusarium* strains

**Fig. 1:**
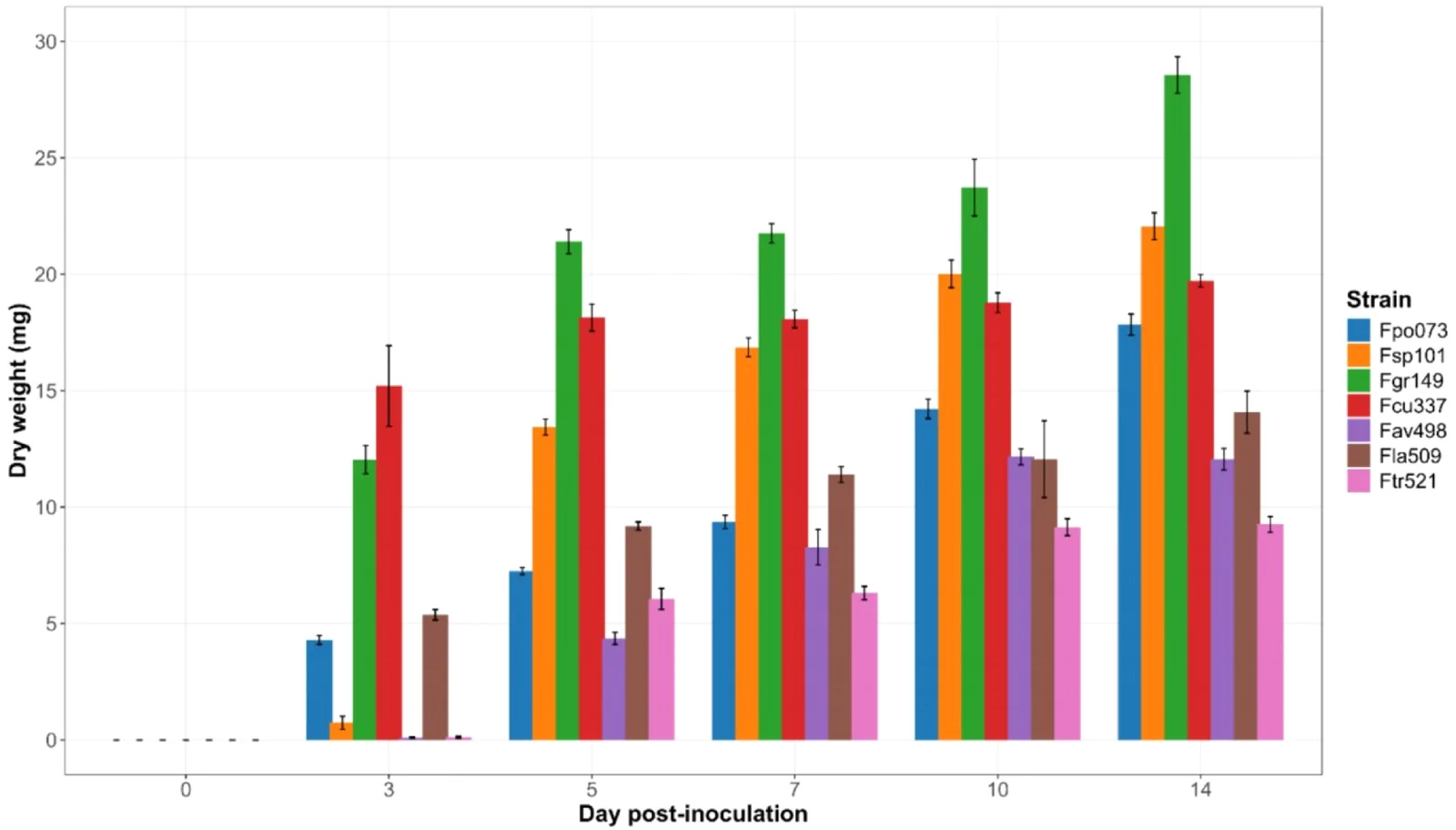
Differential growth, as a function of time, between seven *Fusarium* strains, cultivated for 14 days in MS medium at 25 °C. Growth was measured with mycelial dry weight previously freeze-dried. Each bar indicates the mean, and vertical bars indicate the standard deviation calculated from five biological replicates per strain and day post-inoculation.

The growth of each *Fusarium* strain (single-strain cultures experiment) was monitored using mycelial dry weight at 3, 5, 7, 10, and 14 days post-inoculation (dpi). Growth kinetics for each of the strains studied, reported in Fig. 1, exhibited clear differences. A lag phase exceeding 3 days was observed for three strains, Fav498, Ftr521, and Fsp101. Fav498 and Ftr521 were also characterized by reduced growth rates compared to the others, which was not the case for Fsp101. Actually, Fsp101, similarly to Fgr149 and Fcu337, was the strain with the highest growth rate in the experimental conditions used in the present study. Altogether, our data indicate that Fgr149 and Fcu337 can be considered as fast-growing strains, while Fav498 and Ftr521, but also Fpo073 and Fla509, appear as slower-growing strains.

### 3.2 Comparative analysis of metabolomic profiles of seven *Fusarium* strains

#### 3.2.1 Endo- and exo-metabolomic profiles

The metabolomic profiles of the seven *Fusarium* strains were characterized both at the intracellular (mycelia; *i.e.*, endometabolome) and extracellular (culture media; *i.e.*, exometabolome) levels. After sampling, extraction, data acquisition, and processing, a total of 932 features were obtained for endometabolomic analysis and 1170 features from exometabolomic analysis, including all time points and strains. The number of metabolite features per strain and time of culture is reported in Table 4. Metabolomic features were considered as detected only when their abundance exceeded tenfold the noise threshold for mycelium samples or the levels measured in non-inoculated medium for culture medium samples. The metabolomic features tables and the raw MS data were deposited in the Recherche Data Gouv repository and are publicly accessible (DOI: 10.57745/SJNC38).

**Table 4:** Number of detected metabolomic features in the mycelium (endometabolome) and in the culture medium (exometabolome) of seven *Fusarium* strains grown for 14 days in MS medium at 25 °C. Metabolomic features were considered as detected only when their abundance was exceeding tenfold the noise threshold for mycelium samples or the levels measured in non-inoculated medium for culture medium samples. For each strain and each time point (3, 5, 7, 10, and 14 dpi), five biological replicates were analyzed.

**Strain**	**Mycelium**					**Cultures medium**				
	**3 dpi**	**5 dpi**	**7 dpi**	**10 dpi**	**14 dpi**	**3 dpi**	**5 dpi**	**7 dpi**	**10 dpi**	**14 dpi**
**Fpo073**	174	177	183	188	215	5	18	18	29	126
**Fsp101**	111	388	414	459	471	3	438	527	661	733
**Fgr149**	367	551	562	542	521	247	782	806	824	823
**Fcu337**	334	528	536	523	545	118	631	735	804	832
**Fav498**	70	184	293	312	284	3	11	117	154	166
**Fla509**	156	146	144	151	169	19	45	42	64	127
**Ftr521**	78	254	299	286	269	6	49	137	182	206

Overall, as observed in Table 4, the number of intracellular features remained stable across time points, except for strains Fav498, Ftr521, and Fsp101, where, at 3 dpi, a lower number of metabolites compared to the others was observed, likely reflecting delayed mycelial growth (Fig. 1). Conversely, Fcu337 and Fgr149 displayed an increase in detected metabolites between 3 and 5 dpi. At 14 dpi, strains Fsp101, Fgr149, and Fcu337 exhibited the richest metabolic profiles (471, 521, and 545 features, respectively), while Fp073, Fav498, Fla509, and Ftr521 displayed reduced metabolite numbers (169–284 features).

Regarding culture supernatants, detected features consistently increased over time for all strains, reflecting the accumulation of secreted metabolites. As observed for intracellular extracts, strain-dependent differences were evidenced at 14 dpi. Fsp101, Fgr149, and Fcu337 were characterized by the most complex exometabolomic profiles (733–832 features), whereas Fp073, Fav498, Fla509, and Ftr521 accumulated fewer metabolites (126–206 features). These differences in metabolite yields may be linked to the biosynthetic potential of each strain, such as its expression in the culture conditions used in the present study, but also to variations in growth kinetics, with fast-growing strains producing more biomass (Fsp101, Fgr149, and Fcu337; Fig. 1) and potentially more metabolites.

**Fig. 2:**
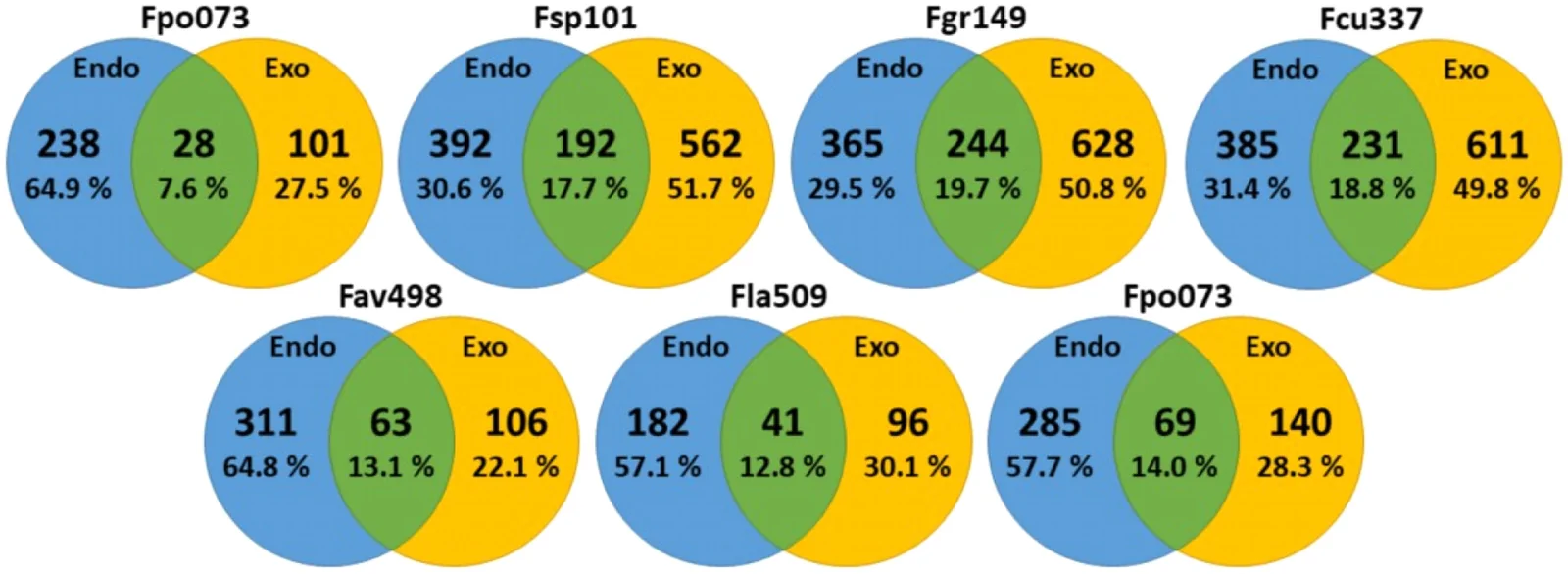
Venn diagrams showing the number and proportion of shared and unique metabolic features between endo- (blue) and exometabolomes (yellow) of seven *Fusarium* strains after 14 days of cultivation in MS medium at 25 °C.

For each strain, endo- and exometabolomes were compared to determine the proportion of strain-specific intra- and extracellular features, as well as the number of shared features (Fig. 2). Shared features were defined using a tolerance of 0.005 Da for calculated molecular weight and 0.033 min for retention time. Strains with the richest metabolomic profiles under our experimental conditions (Fsp101, Fgr149, and Fcu337) displayed a higher proportion of strain-specific extracellular features (51.7 %, 50.8 %, and 49.8 %, respectively) compared to intracellular ones (30.6 %, 29.5 %, and 31.4 %, respectively; Fig. 2). In contrast, strains Fp073, Fav498, Fla509, and Ftr521 showed the opposite trend, with a lower proportion of extracellular-specific features (27.5 %, 22.1 %, 30.1 %, and 28.3 %, respectively) compared to intracellular-specific ones (64.9 %, 64.8 %, 57.1 %, and 57.7 %, respectively; Fig. 2). Across all strains, shared features represented only 7.6–19.7 % of the total detected metabolome. These patterns suggest differential metabolic activities among strains, where the higher proportion of specific extracellular metabolites in Fsp101, Fgr149, and Fcu337 may reflect a more active secondary metabolism compared to the other strains in our experimental conditions.

Among the secondary metabolites retrieved, several mycotoxins were putatively annotated and verified by injection of a pooled mix of mycotoxin standards (*i.e.*, ENN A, ENN A1, ENN B, ENN B1, DON, ADON, NIV, FX, ZEN, T2, HT2, DAS). NIV, ZEN, and BEA, known to be produced by several strains used in this study (Table 1), were not detected. ENNs were identified in both mycelia and culture media of Fav498 and Ftr521. While Enniatin A1, Enniatin B, and Enniatin B1 were found in both compartments, Enniatin A was restricted to the mycelium. For trichothecene-producing strains, both TCTA and TCTB were detected. Within TCTA, T2, HT2, and DAS were identified. T-2 Toxin was consistently observed in both mycelia and culture media of Fsp101 and Fla509. In contrast, HT-2 Toxin was detected only in the culture medium of Fsp101. DAS was identified in the culture media of Fpo073 and Fsp101. Although the Fla509 strain was described to produce both T2 and DAS in previous experiments (Garcia et al., 2025), these mycotoxins were not detected with the untargeted approach used in this study. Regarding Fpo073, no ENNs, BEA, and TCTB (*i.e.*, NIV/FX) were detected in its metabolomics profiles. For TCTB, DON, ADON, and FX were annotated. DON and 15-ADON were retrieved in Fgr149 samples, with 15-ADON found in mycelium and culture medium, while DON was only observed in culture medium. FX was found in both compartments for Fcu337 samples, while NIV was not detected.

#### 3.2.2 Principal component analysis of metabolomic profiles

**Fig. 3:**
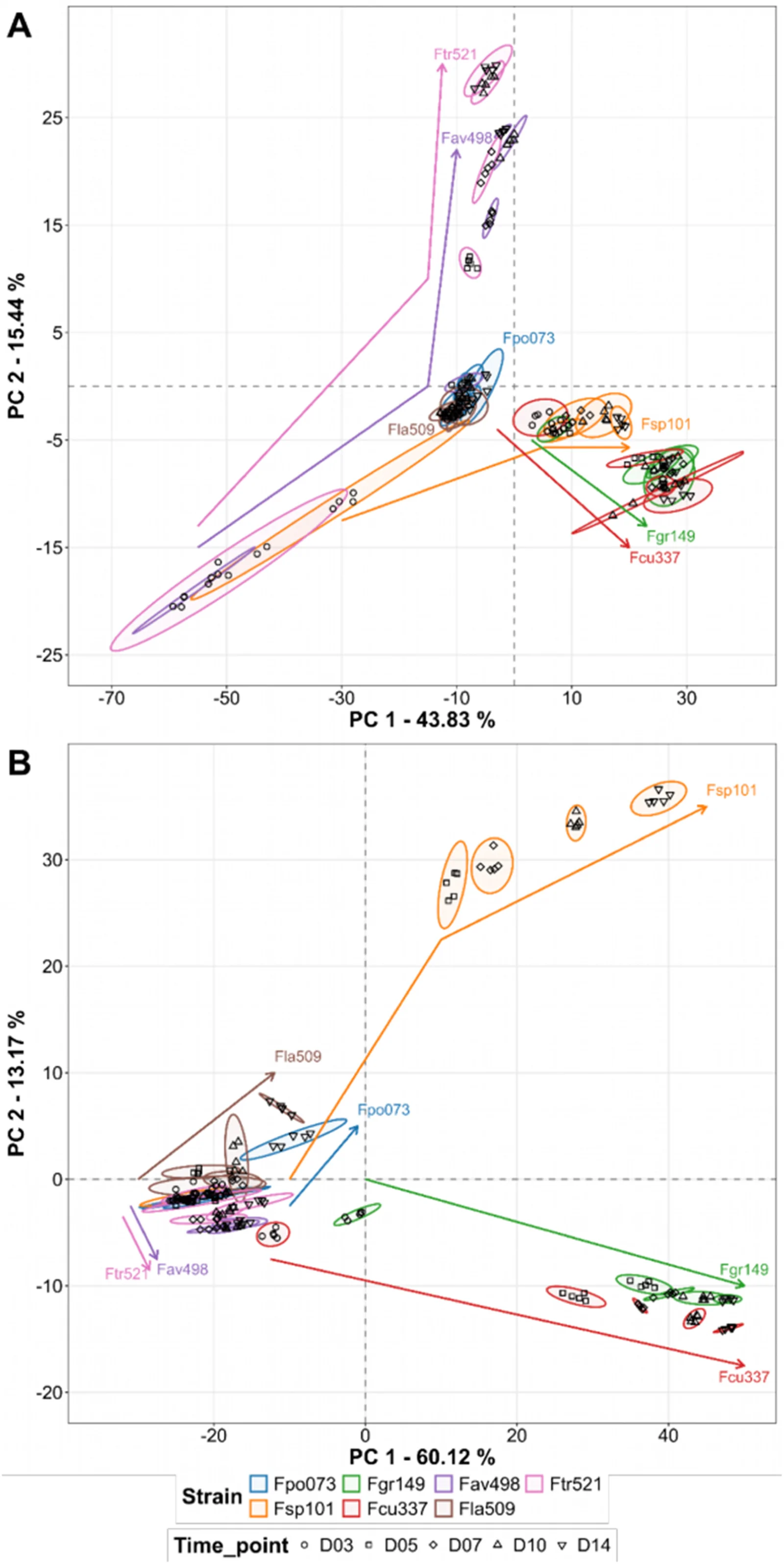
Principal component analysis (PCA) of the endo- and exometabolomic profiles of seven *Fusarium* strains, cultivated in MS medium at 25 °C. (A) Score plot of endometabolomic profiles analysis performed on 175 individuals, described by 932 features (B). Score plot of exometabolomic profiles analysis performed on 175 individuals, described by 1170 features. On score plots, each point represents a sample, and ellipses represent the 95 % confidence interval based on 5 biological replicates. The arrows, added manually, indicate the evolution of metabolomic profiles as a function of time for each strain.

To compare metabolomic profiles between strains, principal component analysis (PCA) was performed, using data from endo- (Fig. 3A) and exometabolome (Fig. 3B) for all strains at the different cultivation time points. For the endometabolome, the analysis included 175 samples represented by 932 mass features. Peak areas were normalized to the biomass dry weight used for metabolite extraction and to quality control samples, then log₂-transformed and auto-scaled. The first two principal components (PCs) explained 59.27 % of the variance (PC1: 43.83 %, PC2: 15.44 %; Fig. 3A). As shown by the 95 % confidence ellipses reported in Fig. 3A, a clear separation was observed according to both factors, time of cultures and strain. For Ftr521 and Fav498, temporal differentiation occurred primarily along PC2. The largest separation was observed for 3 and 5 dpi, coinciding with the time interval in which exponential growth started (Fig. 1) and with a significant increase in the number of detected metabolites (Table 4). While endometabolomic profiles of the two previous strains were very close at 3 and 5 dpi, significant differences were evidenced at 14 dpi, with no ellipses overlap. For Fsp101, Fgr149, and Fcu337, differentiation between time points occurred along both PC1 and PC2, with the greatest separation between 3 and 5 dpi, followed by minimal change after 5 dpi. This trend likely reflects the plateau phase of fungal growth (Fig. 1) and stabilization of detected metabolite numbers (Table 4). Fgr149 and Fcu337 exhibited highly similar endometabolomic profiles with overlapping ellipses throughout the time course, whereas Fsp101 was distinct. For Fpo073 and Fla509, metabolic profiles did not show any demarcation whatever the culture time. At 14 dpi, their metabolomic profiles were similar to each other but distinct from those of other strains.

Regarding exometabolomic profiles, 175 samples described by 1170 features were included in the PCA analysis. Peak areas were normalized to quality control samples, log₂-transformed, and auto-scaled. The first two PCs explained 73.29 % of the variance (PC1: 60.12 %, PC2: 13.17 %; Fig. 3B). In the score plot reported in Fig. 3B, clear separation according to time and strain factors was observed. For Fgr149 and Fcu337, temporal differentiation occurred mainly along PC1; exometabolomic profiles remained distinct between time points, reflecting continuous secretion and accumulation of different metabolites. The largest separation was observed between 3 and 5 dpi and could be related to their growth profile, 5 dpi corresponding to the beginning of the plateau (Fig. 1). For Fpo073, Fsp101, and Fla509, temporal differentiation occurred along both PCs, where Fsp101 showed the greatest temporal separation, particularly between 3 and 5 dpi. The three previous strains displayed distinct profiles, with profiles of Fpo073 and Fla509 appearing closer to each other than to Fsp101. Throughout the time course, Fsp101 exometabolic profiles remained close to those associated with Fgr149 and Fcu337 along PC1 and were separated only along PC2, which accounted for 13.17 % of the variance. For Fav498 and Ftr521, differentiation between time points occurred primarily along PC2, with weak separation. At 14 dpi, their exometabolomic profiles were close but distinct, as indicated by the lack of overlap of the ellipses. Exometabolic profiles of Fav498 and Ftr521 remained close to those of Fpo073 and Fla509, consistent with the reduced number of secreted metabolites observed for these four strains (Table 4).

#### 3.2.3 Hierarchical clustering analysis of metabolomic profiles

**Fig. 4:**
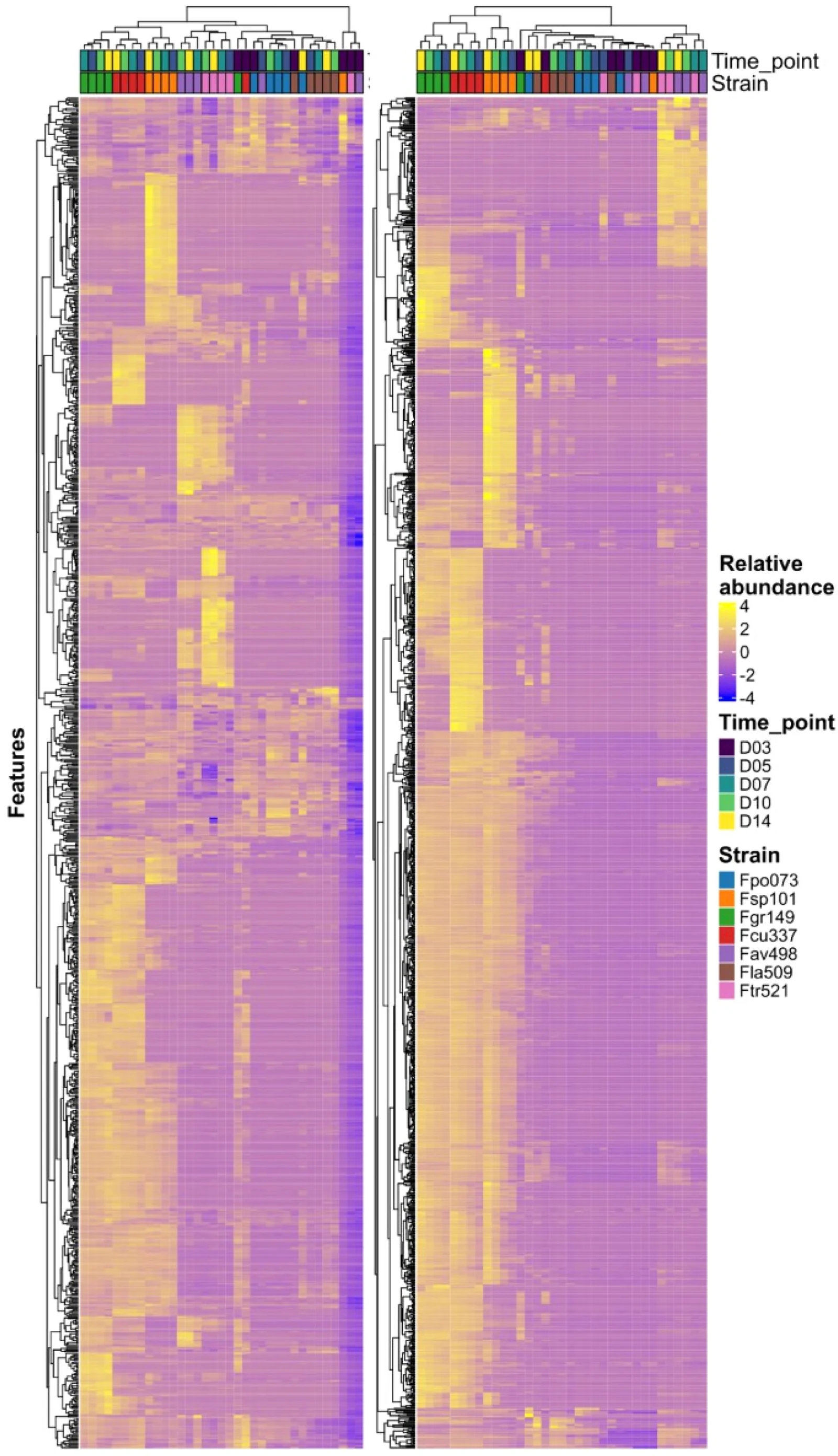
Hierarchical clustering heatmaps of endo- and exometabolomic features detected in the mycelia and culture media of seven *Fusarium* strains from seven species associated with *Fusarium* Head Blight, cultivated in MS medium at 25 °C. (A) Heatmap of endometabolomic profiles based on 175 samples and 932 detected features. (B) Heatmap of exometabolomic profiles based on 175 samples and 1170 detected features. Feature abundances were log-transformed and auto-scaled prior to analysis. Samples and features were hierarchically clustered using Euclidean distance and complete-linkage based on the mean abundance of 5 biological replicates. Color intensity indicates relative feature abundance, with yellow representing higher abundance and purple/blue representing lower abundance or absence.

Hierarchical clustering analysis was performed based on the degree of similarity of metabolite abundance profiles in all mycelia and all culture supernatants (Fig. 4). Samples and features were clustered using Euclidean distance and the complete-linkage method. Metabolites with similar abundance patterns were positioned closer together. As shown in the heatmap and dendrogram reported in Fig. 4, samples were primarily clustered according to the strain, and secondarily to the culture time, especially from 5 to 14 dpi, consistent with the differentiation observed in PCA analysis in Fig. 3.

Regarding endometabolomes (Fig. 4A), a first major cluster gathered the profiles associated with mycelia of Fsp101, Fcu337, and Fgr149, which shared a large number of metabolites with similar relative abundance. Within this cluster, the metabolomic profiles of Fcu337 and Fgr149 exhibited more similarities to each other than to Fsp101. Despite their overall similarity, Fcu337, Fgr149, and Fsp101 profiles were also characterized by strain-specific metabolites, highlighting the occurrence of distinct metabolic signatures. A second cluster gathered Fav498 and Ftr521. Finally, Fpo073 and Fla509 were grouped into a third cluster, closer to Fav498 and Ftr521 than to the metabolically rich strains (Fsp101, Fcu337, Fgr149). Additionally, a subset of common metabolites was retrieved, shared across all strains, indicating the presence of a conserved core-endometabolome.

Regarding exometabolomes, clustering patterns corroborated those based on endometabolome data, with samples mainly clustered according to the strain (Fig. 4B). A first cluster encompassing the exometabolomic profiles of Fsp101, Fgr149, and Fcu337 was evidenced. As for endometabolome analysis, Fcu337 and Fgr149 were more closely related to each other than to Fsp101, while strain-specific metabolites could be highlighted. Fig. 4B also indicated the presence of a second cluster that gathered Fav498 and Ftr521, and a last one composed of Fpo073 and Fla509. For this last cluster, the occurrence of cluster-specific metabolites was less visible.

Overall, the hierarchical clustering analysis allowed us to identify similarities and differences among metabolomics profiles of the *Fusarium* strains. It revealed 3 main clusters of strains: (cluster 1) Fsp101, Fgr149, and Fcu337, with Fgr149 and Fcu337 closely related; (cluster 2) Fav498 and Ftr521; (cluster 3) Fpo073 and Fla509, which are closer to the second cluster than the first one.

#### 3.2.4 Strain biomarkers and shared metabolites identified by upset plot analysis

**Fig. 5:**
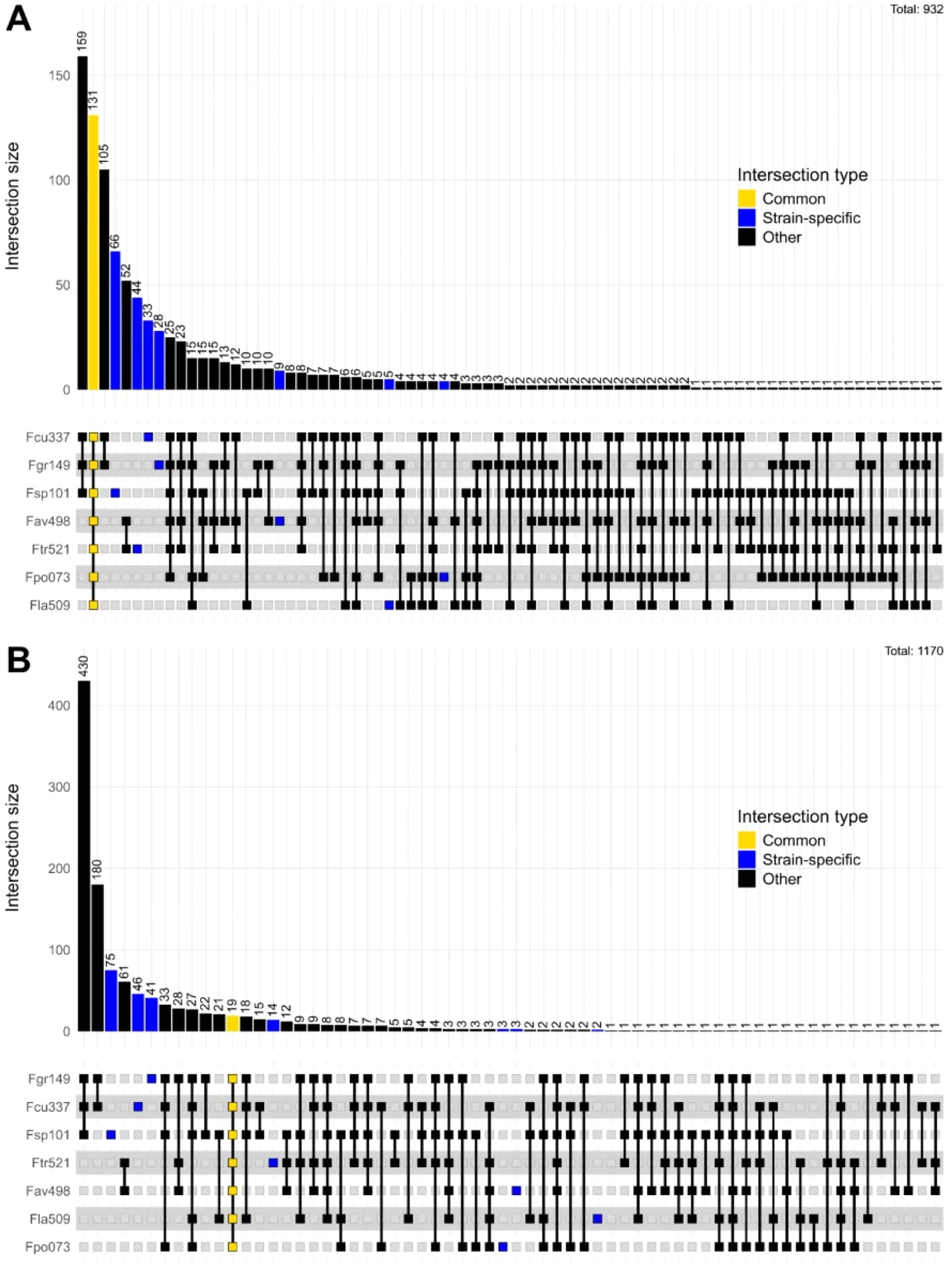
Upset plots of detected features highlighting discriminant (blue) and common (yellow) features between seven *Fusarium* strains, cultivated for 14 days in MS medium at 25 °C. (A) Upset plot of endometabolomic features between *Fusarium* strains based on 175 individuals, described by 1170 features. (B) Upset plot of exometabolomic features between *Fusarium* strains based on 175 individuals, described by 932 features. Metabolomic features were considered, as detected, only when abundance was exceeding tenfold the thresholds of noise for endometabolomic features, or, in the inoculated condition, for exometabolomic features. All time points were considered at once in this analysis (3, 5, 7, 10, and 14 dpi).

An upset plot analysis was conducted to highlight sets of metabolites shared by all seven strains, by subsets of strains, or uniquely present in individual isolates. Upset plots were constructed using the features detected in the LC-HRMS analysis for both endometabolomes and exometabolomes, presented in Fig. 5A and B, respectively (information related to these features is available in the Recherche Data Gouv repository DOI: 10.57745/SJNC38).

The upset plot built with endometabolome data revealed 131 metabolites that were common to all strains. The number of shared metabolites was significantly lower, equal to 19, when considering supernatants. The previous data support the presence of conserved intracellular metabolic pathways between the seven strains. Among the metabolites composing the intracellular “core-metabolome”, some putative features were annotated with a confidence level of identification of 2a (CLI = 2a) (Schymanski et al., 2014). These putatively annotated metabolites were mostly related to primary metabolites, including l-Phenylalanine (Myc_Var0000057), S-Methyl-5′-thioadenosine (Myc_Var0000157), 4-Aminobenzoate (Myc_Var0000337), and an oxidized flavodoxin (Myc_Var0000882).

The upset plot also highlights the similarities among subsets of strains, including those previously clustered (Fig. 4) in three main groups (cluster 1: Fsp101, Fgr149, and Fcu337; cluster 2: Fav498 and Ftr521; cluster 3: Fpo073 and Fla509). Shared metabolites between those clusters of strains, with a CLI equal to or lower than 3, are listed in Table 5. A complete list is also available in the supplementary material (Table S1). A substantial number of shared metabolites were retrieved in the metabolic profiles of cluster 1, composed of Fsp101, Fgr149, and Fcu337 belonging to three species of the FSAMSC, with 159 common features in mycelium and 430 common features in culture medium (Fig. 5). In supernatants, the features putatively annotated with a confidence level of identification lower or equal to 3, and described as being produced by the fungal kingdom, were mostly related to terpene-derived molecules (Table 5). It included putative trichothecenes mycotoxin precursors (*e.g.*, Trichodermol isomers: Med_Var0000051, Med_Var0000218, Med_Var0000258, Myc_Var0000174, Myc_Var0000499; Isotrichodiol isomers: Med_Var0000667, Med_Var0000825, Myc_Var0000166, Myc_Var0000178; Trichotriol isomers: Med_Var0000027, Med_Var0000093, Myc_Var0000326, Myc_Var0000336; 12,13-Epoxytrichothec-9-ene isomers: Med_Var0000068, Med_Var0000148; 12,13-Epoxy-9,10-trichoene-2-ol isomers: Med_Var0000318, Med_Var0000396, Myc_Var0000043, Myc_Var0000435; 2-Hydroxytrichodiene isomers: Med_Var0000224, Myc_Var0000338, Myc_Var0000564) as well as other putative terpenoids (*e.g.*, Trichodermarin F isomers: Med_Var0000311, Med_Var0000329, Myc_Var0000421, Myc_Var0000853; Curvularol isomers: Med_Var0000003, Myc_Var0000158, Myc_Var0000214; Guignardone I isomers: Med_Var0000057, Med_Var0000229, Myc_Var0000112). Some common polyketides were also putatively identified (*e.g.*, Benquoine: Med_Var0000437; Zosteropenilline analogs: Med_Var0000501, Med_Var0000821; Cytospolide Q isomers: Myc_Var0000055, Myc_Var0000621; Harzianol acid: Myc_Var0000443), as well as one putative alkaloid (Pyreudione B_iso5: Med_Var0000813) and one putative fatty acid ((1S,2R)-3-oxo-2-pentylcyclopentane-1-butyric acid methyl ester: Med_Var0000700) (Table 5). In accordance with PCA and hierarchical clustering heatmap results, Fgr149 and Fcu337 metabolomic profiles were shown to share a significant number of metabolites (105 in mycelium, 180 in culture medium), supporting their close metabolic relatedness.

**Table 5:** Shared metabolites between clusters of *Fusarium* strains, identified through Upset plot analysis with a confidence level of identification equal to or lower than 3 and putatively annotated as produced in the fungal kingdom. Cluster 1 includes Fsp101, Fgr149, and Fcu337; Cluster 2 includes Fav498 and Ftr521; Cluster 3 includes Fpo073 and Fla509. Each feature is described by: an identifier (Variable_ID) with a prefix indicating detection in the endometabolome (Myc) or exometabolome (Med); a retention time in minutes (RT_min); a mass-to-charge ratio associated with an adduct (*m/z*_of_adduct and Adduct); a putative molecular formula and name (Putative_formula and Putative_name) as assigned by Compound Discoverer software; and a confidence level of identification (Schymanski et al., 2014).

Cluster	Variable_ID	Putative_formula	Putative_annotation	RT_ min	*m/z*_of_adduct	Adduct	Confidence_level_of_ identification
(1)	Med_Var0000003	C15 H22 O4	Curvularol_iso1	6.955	267.15,918	[M+H]+1	3
(1)	Med_Var0000027	C15 H24 O4	Trichotriol_iso1	6.501	269.17,501	[M+H]+1	3
(1)	Med_Var0000051	C15 H22 O3	Trichodermol_iso3	6.5	251.16,434	[M+H]+1	3
(1)	Med_Var0000057	C17 H26 O5	Guignardone I_iso1	6.067	333.16,736	[M+Na]+1	3
(1)	Med_Var0000067	C15 H20 O3	Arecolactone_iso1	6.206	249.1487	[M+H]+1	3
(1)	Med_Var0000068	C15 H22 O2	12,13-Epoxytrichothec-9-ene_iso1	7.241	235.1695	[M+H]+1	3
(1)	Med_Var0000075	C15 H22 O4	Curvularol_iso3	7.523	267.15,928	[M+H]+1	3
(1)	Med_Var0000093	C15 H24 O4	Trichotriol_iso3	5.518	269.17,493	[M+H]+1	3
(1)	Med_Var0000132	C15 H22 O4	Curvularol_iso5	5.889	267.15,928	[M+H]+1	3
(1)	Med_Var0000138	C15 H20 O3	Arecolactone_iso2	6.108	249.14,871	[M+H]+1	3
(1)	Med_Var0000148	C15 H22 O2	12,13-Epoxytrichothec-9-ene_iso2	7.471	235.16,948	[M+H]+1	3
(1)	Med_Var0000151	C15 H20 O3	Chaetothyrin C	7.522	249.14,873	[M+H]+1	3
(1)	Med_Var0000153	C15 H22 O5	Stereumin U	6.026	265.14,364	[M+H-H2O]+1	3
(1)	Med_Var0000218	C15 H22 O3	Trichodermol_iso4	7.468	251.1644	[M+H]+1	3
(1)	Med_Var0000224	C15 H24 O	2-Hydroxytrichodiene_iso2	8.323	203.17,968	[M+H-H2O]+1	3
(1)	Med_Var0000229	C17 H26 O5	Guignardone I_iso2	6.169	333.16,744	[M+Na]+1	3
(1)	Med_Var0000239	C15 H18 O3	9a-Hydroxy-3,8a-dimethyl-5-methylene4,4a,5,6,9,9a-hexahydronaphtho[2,3-b]furan-2(8aH)-one_iso1	8.279	247.13,302	[M+H]+1	3
(1)	Med_Var0000258	C15 H22 O3	Trichodermol_iso5	7.649	251.16,443	[M+H]+1	3
(1)	Med_Var0000273	C15 H22 O3	Trichodermol_iso6	7.834	251.16,439	[M+H]+1	3
(1)	Med_Var0000275	C15 H22 O3	Trichodermol_iso7	6.768	273.14,628	[M+Na]+1	3
(1)	Med_Var0000311	C17 H26 O5	Trichodermarin F_iso1	7.446	333.16,752	[M+Na]+1	3
(1)	Med_Var0000318	C15 H24 O2	12,13-Epoxy-9,10-trichoene-2-ol_iso3	5.36	237.18,519	[M+H]+1	3
(1)	Med_Var0000321	C15 H20 O3	Arecolactone_iso3	7.108	271.13,068	[M+Na]+1	3
(1)	Med_Var0000326	C15 H24 O4	Trichotriol_iso5	6.672	269.17,496	[M+H]+1	3
(1)	Med_Var0000329	C17 H26 O5	Trichodermarin F_iso2	6.636	333.16,737	[M+Na]+1	3
(1)	Med_Var0000337	C15 H22 O3	Trichodermol_iso8	8.692	251.16,439	[M+H]+1	3
(1)	Med_Var0000375	C15 H20 O3	2-oxoeremophila-1(10),11(13)‑dien-12-oic acid	7.044	249.14,877	[M+H]+1	3
(1)	Med_Var0000383	C17 H26 O5	Trichodermarin F_iso3	8.124	333.16,751	[M+Na]+1	3
(1)	Med_Var0000389	C15 H20 O3	Arecolactone_iso4	8.751	249.14,875	[M+H]+1	3
(1)	Med_Var0000396	C15 H24 O2	12,13-Epoxy-9,10-trichoene-2-ol_iso4	5.46	237.18,514	[M+H]+1	3
(1)	Med_Var0000399	C17 H26 O5	Penisporolide B	7.17	311.18,559	[M+H]+1	3
(1)	Med_Var0000402	C15 H20 O4	Trichodermaloid C	5.003	265.14,372	[M+H]+1	3
(1)	Med_Var0000409	C15 H24 O2	12,13-Epoxy-9,10-trichoene-2-ol_iso5	5.823	237.18,516	[M+H]+1	3
(1)	Med_Var0000437	C14 H20 O2	Benquoine	6.863	221.15,387	[M+H]+1	3
(1)	Med_Var0000453	C15 H22 O5	Piltunine A_iso1	5.65	283.15,428	[M+H]+1	3
(1)	Med_Var0000467	C15 H20 O3	Arecolactone_iso5	5.706	249.14,879	[M+H]+1	3
(1)	Med_Var0000481	C15 H20 O3	Arecolactone_iso6	5.772	249.14,877	[M+H]+1	3
(1)	Med_Var0000501	C15 H24 O5	Zosteropenilline L	5.63	285.16,991	[M+H]+1	3
(1)	Med_Var0000510	C15 H22 O	Anaephene A_iso5	6.597	219.17,459	[M+H]+1	3
(1)	Med_Var0000531	C17 H26 O5	Trichodermarin F_iso4	6.917	333.16,759	[M+Na]+1	3
(1)	Med_Var0000532	C15 H18 O3	9a-Hydroxy-3,8a-dimethyl-5-methylene4,4a,5,6,9,9a-hexahydronaphtho[2,3-b]furan-2(8aH)-one_iso2	6.884	247.13,316	[M+H]+1	3
(1)	Med_Var0000542	C15 H20 O3	Arecolactone_iso7	5.314	249.14,882	[M+H]+1	3
(1)	Med_Var0000567	C15 H24 O2	12,13-Epoxy-9,10-trichoene-2-ol_iso8	5.693	237.1852	[M+H]+1	3
(1)	Med_Var0000571	C15 H26 O4	12-Hydroxy-6‑epi-albrassitriol	7.353	293.1726	[M+Na]+1	3
(1)	Med_Var0000574	C15 H24 O4	Trichotriol_iso7	5.417	269.17,501	[M+H]+1	3
(1)	Med_Var0000632	C15 H20 O4	Rhinomilisin H_iso2	5.648	265.14,375	[M+H]+1	3
(1)	Med_Var0000666	C15 H26 O4	Diaporol B	6.689	253.18,013	[M+H-H2O]+1	3
(1)	Med_Var0000667	C15 H24 O3	Isotrichodiol_iso5	6.695	253.18,012	[M+H]+1	3
(1)	Med_Var0000671	C15 H24 O2	12,13-Epoxy-9,10-trichoene-2-ol_iso9	3.56	237.18,516	[M+H]+1	3
(1)	Med_Var0000695	C15 H24 O2	12,13-Epoxy-9,10-trichoene-2-ol_iso10	8.735	237.18,516	[M+H]+1	3
(1)	Med_Var0000700	C15 H26 O3	(1S,2R)-3-oxo-2-pentylcyclopentane-1-butyric acid methyl ester	6.356	255.19,578	[M+H]+1	3
(1)	Med_Var0000813	C17 H27 N O3	Pyreudione B_iso5	4.2	294.20,671	[M+H]+1	3
(1)	Med_Var0000821	C15 H24 O5	Zosteropenilline E_iso1	5.091	285.16,995	[M+H]+1	3
(1)	Med_Var0000825	C15 H24 O4	Isotrichodiol_iso12	6.012	291.15,697	[M+Na]+1	3
(1)	Med_Var0000933	C15 H22 O5	Clitocybulol E	5.176	283.15,431	[M+H]+1	3
(1)	Med_Var0000940	C15 H18 O3	9a-Hydroxy-3,8a-dimethyl-5-methylene4,4a,5,6,9,9a-hexahydronaphtho[2,3-b]furan-2(8aH)-one_iso3	4.773	247.13,321	[M+H]+1	3
(1)	Med_Var0001024	C15 H22 O5	Piltunine A_iso3	6.656	283.15,432	[M+H]+1	3
(1)	Med_Var0001048	C15 H24 O4	Isotrichodiol_iso13	6.104	269.17,505	[M+H]+1	3
(1)	Med_Var0001094	C15 H24 O5	13-hydroxypereniporin A	6.233	307.15,181	[M+Na]+1	3
(1)	Med_Var0001102	C15 H22 O5	Piltunine A_iso4	7.648	283.15,427	[M+H]+1	3
(1)	Med_Var0001129	C15 H20 O4	Trichodermaloid C	4.876	247.13,325	[M+H-H2O]+1	3
(1)	Myc_Var0000043	C15 H24 O2	12,13-Epoxy-9,10-trichoene-2-ol_iso1	8.915	237.18,491	[M+H]+1	3
(1)	Myc_Var0000055	C15 H26 O5	Cytospolide Q_iso1	6.506	309.16,713	[M+Na]+1	3
(1)	Myc_Var0000096	C15 H24 O4	Trichotriol_iso1	6.506	269.1748	[M+H]+1	3
(1)	Myc_Var0000112	C17 H26 O5	Guignardone I	6.073	333.16,714	[M+Na]+1	3
(1)	Myc_Var0000154	C17 H26 O5	Arundinol C	7.645	333.16,711	[M+Na]+1	3
(1)	Myc_Var0000158	C15 H22 O4	Curvularol_iso2	4.887	289.14,102	[M+Na]+1	3
(1)	Myc_Var0000166	C15 H24 O3	Isotrichodiol_iso2	7.253	253.17,988	[M+H]+1	3
(1)	Myc_Var0000174	C15 H22 O3	Trichodermol_iso3	6.506	251.16,421	[M+H]+1	3
(1)	Myc_Var0000178	C15 H24 O4	Isotrichodiol_iso6	4.492	291.1566	[M+Na]+1	3
(1)	Myc_Var0000214	C15 H22 O4	Curvularol_iso3	7.533	267.15,913	[M+H]+1	3
(1)	Myc_Var0000231	C15 H20 O3	Arecolactone_iso1	6.22	249.14,859	[M+H]+1	3
(1)	Myc_Var0000238	C15 H24 O4	Isotrichodiol_iso7	4.92	291.15,655	[M+Na]+1	3
(1)	Myc_Var0000248	C15 H20 O3	2-oxoeremophila-1(10),11(13)‑dien-12-oic acid	7.214	249.14,865	[M+H]+1	3
(1)	Myc_Var0000308	C15 H20 O3	Arecolactone_iso2	7.534	249.14,861	[M+H]+1	3
(1)	Myc_Var0000310	C15 H24 O3	Isotrichodiol_iso3	7.477	253.17,989	[M+H]+1	3
(1)	Myc_Var0000326	C15 H24 O4	Trichotriol_iso3	6.872	291.15,671	[M+Na]+1	3
(1)	Myc_Var0000336	C15 H24 O4	Trichotriol_iso4	5.527	269.17,481	[M+H]+1	3
(1)	Myc_Var0000338	C15 H24 O	2-Hydroxytrichodiene_iso4	8.339	203.17,956	[M+H-H2O]+1	3
(1)	Myc_Var0000355	C15 H20 O3	Arecolactone_iso3	6.118	249.14,862	[M+H]+1	3
(1)	Myc_Var0000398	C15 H22 O4	Curvularol_iso4	5.899	267.15,922	[M+H]+1	3
(1)	Myc_Var0000421	C17 H26 O5	Trichodermarin F_iso1	6.174	333.16,725	[M+Na]+1	3
(1)	Myc_Var0000435	C15 H24 O2	12,13-Epoxy-9,10-trichoene-2-ol_iso3	5.281	237.18,503	[M+H]+1	3
(1)	Myc_Var0000443	C15 H24 O5	Harzianol acid	5.636	307.15,165	[M+Na]+1	3
(1)	Myc_Var0000492	C15 H24 O4	Isotrichodiol_iso8	6.108	291.15,677	[M+Na]+1	3
(1)	Myc_Var0000499	C15 H22 O3	Trichodermol_iso4	6.774	273.14,623	[M+Na]+1	3
(1)	Myc_Var0000501	C15 H22 O3	Trichodermol_iso5	7.839	251.16,433	[M+H]+1	3
(1)	Myc_Var0000510	C15 H22 O5	Stereumin U	6.039	265.14,357	[M+H-H2O]+1	3
(1)	Myc_Var0000564	C15 H24 O	2-Hydroxytrichodiene_iso5	11.537	221.19,014	[M+H]+1	3
(1)	Myc_Var0000613	C15 H20 O3	Arecolactone_iso4	7.119	271.13,058	[M+Na]+1	3
(1)	Myc_Var0000621	C15 H26 O5	Cytospolide Q_iso2	5.771	309.1673	[M+Na]+1	3
(1)	Myc_Var0000659	C15 H22 O4	6-dehydroxy-6-oxopereniporin A_iso1	5.783	305.11,509	[M+K]+1	3
(1)	Myc_Var0000702	C15 H24 O4	Trichotriol_iso5	6.681	269.17,487	[M+H]+1	3
(1)	Myc_Var0000719	C15 H22 O4	Curvularol_iso5	7.448	267.15,923	[M+H]+1	3
(1)	Myc_Var0000757	C15 H22 O4	Curvularol_iso6	5.715	267.15,927	[M+H]+1	3
(1)	Myc_Var0000847	C15 H24 O2	12,13-Epoxy-9,10-trichoene-2-ol_iso5	5.463	237.18,505	[M+H]+1	3
(1)	Myc_Var0000853	C17 H26 O5	Trichodermarin F_iso2	7.455	333.16,741	[M+Na]+1	3
(1)	Myc_Var0000928	C15 H24 O3	Isotrichodiol_iso5	8.164	253.17,999	[M+H]+1	3
(2)	Med_Var0000001	C34 H59 N3 O9	Enniatin B1	14.758	671.45,885	[M+NH4]+1	1
(2)	Med_Var0000002	C35 H61 N3 O9	Enniatin A1	15.556	685.47,431	[M+NH4]+1	1
(2)	Med_Var0000004	C33 H57 N3 O9	Enniatin B	14.117	640.41,696	[M+H]+1	1
(2)	Myc_Var0000002	C34 H59 N3 O9	Enniatin B1	14.559	654.43,206	[M+H]+1	1
(2)	Myc_Var0000003	C33 H57 N3 O9	Enniatin B	13.956	640.41,628	[M+H]+1	1
(2)	Myc_Var0000005	C35 H61 N3 O9	Enniatin A1	15.391	668.44,827	[M+H]+1	1
(2)	Myc_Var0000064	C36 H63 N3 O9	Enniatin A	16.614	682.46,353	[M+H]+1	1

For cluster 3, composed of the strains Fla509 and Fpo073, two strains from two species also belonging to the FSAMSC, few cluster-specific metabolites were observed, with only five common features in mycelium and one common feature in culture medium. None of them has a confidence level of identification below or equal to 3 (Table S1).

In addition, the two strains, Fav498 and Ftr521 that belong to FTSC and compose cluster 2, displayed a notable overlap in metabolite composition, sharing 52 features in their endometabolomic profiles and 61 in exometabolomic ones. Among these features, those annotated were mostly related to putative nonribosomal peptides. In addition to common ENNs, other analogs from the J and P families (*e.g.*, Enniatin J1: Med_Var0000123, Myc_Var0000008; Enniatin J2: Myc_Var0000032, Myc_Var0000032; Enniatin P1: Med_Var0000645, Med_Var0000828) were putatively annotated but with a low CLI (CLI = 4, Table S1).

The upset plot analysis also led to evidence strain-specific metabolites, referred to in this study as biomarkers, *i.e.*, metabolites that were only retrieved in the profiles of one specific strain. Biomarkers of each *Fusarium*, identified in this study with a CLI equal to or lower than 3, are listed in Table 6, while tables gathering all the biomarkers are also available in the supplementary material (Table S2 for intracellular biomarkers, Table S3 for extracellular biomarkers). Fsp101, Fcu337, Fgr149, and Ftr521 exhibited the largest numbers of biomarkers both at the intracellular (66, 33, 28, and 44 features, respectively) and extracellular (75, 46, 41, and 14 features, respectively) levels. In contrast, Fav498, Fpo073, and Fla509 displayed very few biomarkers, both at the intracellular (9, 4, and 5 features, respectively) and extracellular (3, 3, and 2 features, respectively) levels. Strain biomarkers that were annotated with a CLI, equal to or lower than 3, and with a putative annotation described in the literature as being produced in the fungal kingdom are gathered in Table 6 and discussed in the following.

**Table 6:** Strain biomarkers identified through Upset plot analysis with a confidence level of identification equal to or lower than 3 and putatively annotated as produced in the fungal kingdom. Each feature is described by the producing strain (Strain producer); an identifier (Variable_ID) with a prefix indicating detection in the endometabolome (Myc) or exometabolome (Med); a retention time in minutes (RT_min); a mass-to-charge ratio associated with an adduct (*m/z*_of_adduct and Adduct); a putative molecular formula and name (Putative_formula and Putative_name) as assigned by Compound Discoverer software; and a confidence level of identification (Schymanski et al., 2014).

Strain producer	Variable_ID	Putative_formula	Putative_annotation	RT_ min	*m/z*_of_adduct	Adduct	Confidence_level_of_ identification
Fcu337	Med_Var0000146	C17 H22 O8	Fusarenon-X	4.523	355.13,896	[M+H]+1	1
Fcu337	Med_Var0000470	C19 H24 O8	3,15-Acetyldeoxynivalenol_iso1	6.775	403.1367	[M+Na]+1	3
Fcu337	Med_Var0000535	C17 H24 O7	Toxin NT-2_iso1	5.153	341.15,989	[M+H]+1	2a
Fcu337	Myc_Var0000317	C19 H26 O8	7,8-Dihydroxycalonectrin	6.613	383.17,018	[M+H]+1	3
Fcu337	Myc_Var0000401	C17 H22 O8	Fusarenon-X	4.53	355.13,887	[M+H]+1	1
Fcu337	Myc_Var0000571	C17 H20 O5	PF1092B_iso3	6.613	305.13,849	[M+H]+1	3
Fcu337	Myc_Var0000720	C19 H24 O8	3,15-Acetyldeoxynivalenol	6.789	403.13,646	[M+Na]+1	3
Fgr149	Med_Var0000087	C15 H20 O6	Deoxynivalenol	3.054	297.13,346	[M+H]+1	1
Fgr149	Med_Var0000310	C17 H22 O7	15-Acetyldeoxynivalenol	5.554	339.14,424	[M+H]+1	1
Fgr149	Med_Var0000728	C15 H18 O5	Hirsutenol D	5.554	279.12,302	[M+H]+1	3
Fgr149	Med_Var0000903	C21 H34 N4 O4	Sclerotiotide B	5.498	204.13,656	[M + 2H]+2	3
Fgr149	Med_Var0001097	C14 H16 O4	Illinitone B	5.555	249.11,246	[M+H]+1	3
Fgr149	Myc_Var0000253	C15 H18 O5	Hirsutenol D	5.565	261.11,216	[M+H-H2O]+1	3
Fgr149	Myc_Var0000430	C17 H22 O7	15-Acetyldeoxynivalenol	5.565	361.12,582	[M+Na]+1	1
Fgr149	Myc_Var0000668	C15 H24 O2	12,13-Epoxy-9,10-trichoene-2-ol_iso4	10.365	237.18,508	[M+H]+1	3
Fgr149	Myc_Var0000715	C15 H22 O2	12,13-Epoxytrichothec-9-ene_iso5	7.929	235.16,937	[M+H]+1	3
Fgr149	Myc_Var0000791	C15 H18 O4	Heterocornol O_iso2	5.687	263.12,792	[M+H]+1	3
Fgr149	Myc_Var0000877	C17 H26 O5	Trichodermarin F_iso3	7.186	311.18,535	[M+H]+1	3
Fpo073	Myc_Var0000256	C18 H18 O8	Isosecosterigmatocystin	8.583	363.1074	[M+H]+1	3
Fsp101	Med_Var0000376	C22 H32 O8	HT-2 Toxin	8.045	425.2174	[M+H]+1	1
Fsp101	Med_Var0000520	C24 H34 O9	T-2 Toxin_iso1	9.33	467.22,799	[M+H]+1	3
Fsp101	Myc_Var0000628	C20 H30 O4	Aphidicolin A54	11.731	357.20,385	[M+Na]+1	3
Ftr521	Myc_Var0000471	C20 H32 O3	Decatamariic acid_iso1	15.103	321.24,258	[M+H]+1	3
Ftr521	Myc_Var0000812	C20 H32 O3	Decatamariic acid_iso2	14.222	321.24,259	[M+H]+1	3

Regarding the strain Fsp101, in addition to the HT-2 toxin, metabolic biomarkers include other putatively annotated terpenoids such as an isomer of T-2 toxin (the T-2 Toxin_iso1 - Med_Var0000520), and Aphidicolin A54 (Myc_Var0000628), a diterpenoid previously identified in the fungus *Botryotinia fuckeliana* MCCC 3A00494 (Niu et al., 2019).

Biomarkers of Fcu337 were shown to comprise, in addition to FX, other putatively annotated TCTB precursors (*e.g.*, 3,15-Acetyldeoxynivalenol: Med_Var0000146, Myc_Var0000720; 7,8-Dihydroxycalonectrin: Myc_Var0000317). In addition, 4,15-Diacetylnivalenol (Med_Var0000395, Myc_Var0000786), a putative NIV precursor, was also annotated but with a low CLI (CLI = 4; Table S2, Table S3). Another trichothecene corresponding to the Toxin NT-2_iso1 (Med_Var0000535), which was previously reported as produced by *F. sporotrichioides* (Ishii and Ueno, 1981; Svoboda et al., 2024), was also putatively annotated with a high confidence level.

When looking at Fgr149 biomarkers, in addition to DON and 15-ADON, various TCTB precursors (*e.g.*, 12,13-Epoxy-9,10-trichoene-2-ol_iso4: Myc_Var0000668; 12,13-Epoxytrichothec-9-ene_iso5: Myc_Var0000715) were putatively annotated. Fgr149 biomarkers also encompass other terpenoids, previously reported as produced by other fungi such as Hirsutenol D (Med_Var0000728, Myc_Var0000253), Illinitone B (Med_Var0001097), and Trichodermarin F_iso3 (Myc_Var0000877) (Gruhn et al., 2007; Klaiklay et al., 2019; Yoo et al., 2006). Besides, one putative polyketide, the Heterocornol O_iso2 (Myc_Var0000791) and one putative nonribosomal peptide, the Sclerotiotide B (Med_Var0000903) (Lei et al., 2019; Zheng et al., 2010), were also identified as Fgr149 biomarkers.

Regarding the strain Ftr521, two diterpenoids, corresponding to two isomers of Decatamariic acid (Myc_Var0000471, Myc_Var0000812), were only retrieved in the mycelia of Ftr521. In the literature, Decatamariic acid was associated with one *Aspergillus* strain, *Aspergillus tamarii* FKI-6817 (Watanabe et al., 2017).

For Fpo073, only one feature was putatively annotated with a high level of confidence. This biomarker could be a putative polyketide Isosecosterigmatocystin (Myc_Var0000256), previously reported as produced by *Aspergillus nidulans* (Yang et al., 2018).

Concerning strain biomarkers of Fav498 and Fla509, no putative annotation with a level of confidence lower than 3 was obtained.

### 3.3 Biomarker dynamics in the co-cultures of several *Fusarium* strains

#### 3.3.1 Strain composition of the co-cultures over time

**Fig. 6:**
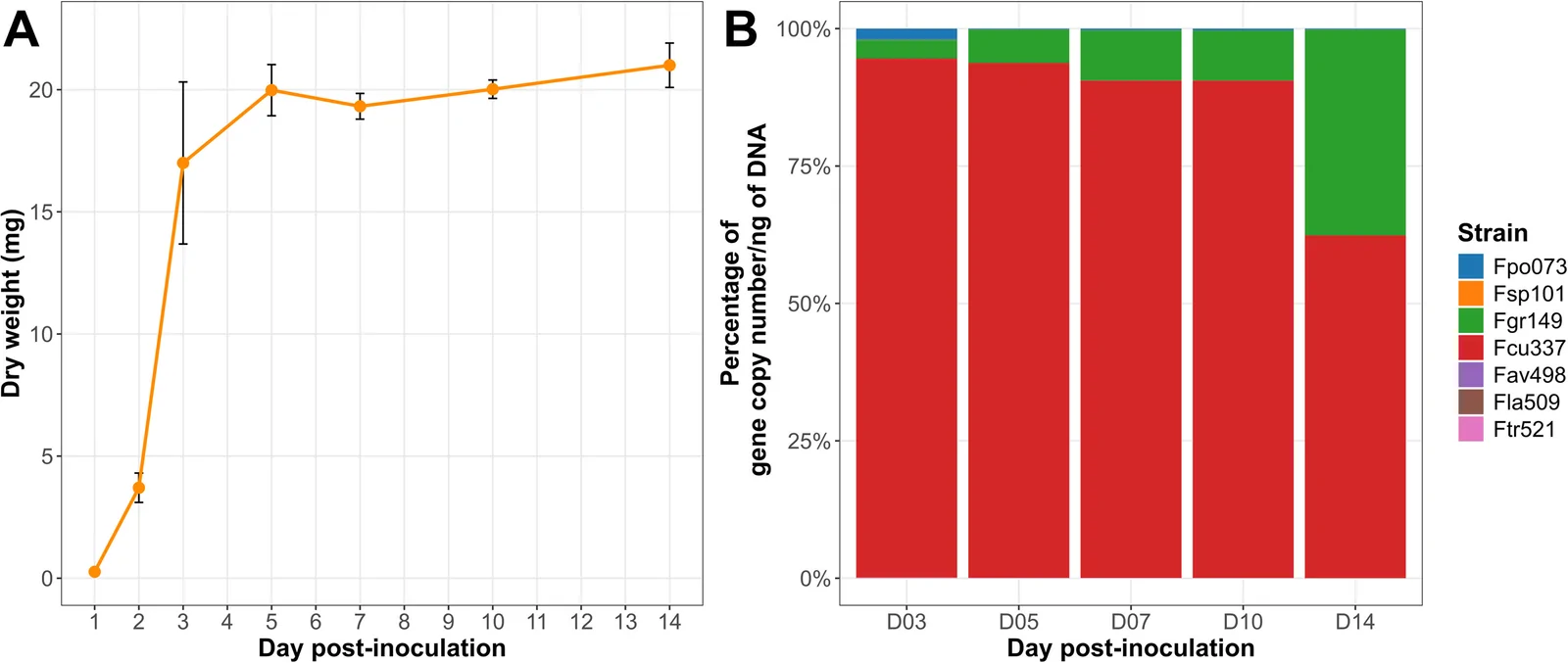
Growth and relative abundance of seven *Fusarium* strains co-inoculated in MS medium at 25 °C for 14 days. (A) Fungal growth was assessed as mycelial dry weight after freeze-drying. Each bar represents the mean of 5 biological replicates. (B) Strain composition was determined by quantifying *TEF1* gene copy number per ng of DNA. Colors indicate the relative contribution of each strain to the total DNA based on five biological replicates.

The kinetics of fungal growth of the *Fusarium* co-cultures, reported in Fig. 6A, showed strong similarities with that of the Fgr149 and Fcu337 strains, *i.e.*, a lag phase inferior to 2 days followed by a fast growth rate and a plateau starting from 3–5 dpi. As shown in Fig. 6B, describing the relative DNA abundance of each strain over the incubation period, Fcu337 was the dominant strain, regardless of the considered culture time. However, the strain composition of the *Fusarium* co-cultures displayed some shifts over time after inoculation. At 3 dpi, 94.44 % of the *TEF1* gene copy number.ng^-1^ of DNA was associated with Fcu337, and only 3.33 % with Fgr149, and 2.04 % with Fpo073. Only traces (< 1 %) of the four other strains (Fsp101, Fav498, Fla509, and Ftr521) were quantified. From 3 dpi to 14 dpi, the relative abundance of Fcu337 was shown to slightly decrease, whereas the percentage of Fgr149 within the co-cultures gradually increased. At 14 dpi, the percentage of Fgr149 *TEF1* gene copy number.ng^-1^ of DNA was close to 37 %, and that related to Fcu337 accounted for around 62 %. These results clearly indicate that Fcu337, followed by Fgr149 and to a lesser extent Fpo073, are the predominant strains under the experimental conditions tested.

#### 3.3.2 Monitoring of strain biomarkers in the *Fusarium* co-cultures

Metabolomics applied to the co-cultures experiment (both endo- and exometabolome) was conducted using a similar procedure as with the single-strain cultures experiment. The metabolomics features tables obtained are publicly available in the Recherche Data Gouv repository (DOI: 10.57745/SJNC38). 421 features were detected in the endometabolome and 364 in the exometabolome. Among the features detected, the mycotoxin FX is the only mycotoxin identified in the co-culture experiments, both in mycelium and culture medium. In parallel, strain biomarkers were manually searched using a tolerance threshold of 0.005 Da for calculated molecular weight and 0.2 min for retention time, combined with MS² spectral comparison in cases of multiple matches. The list of biomarkers retrieved within the *Fusarium* co-cultures experiment are available in the supplementary material Table S4 and Table S5.

**Fig. 7:**
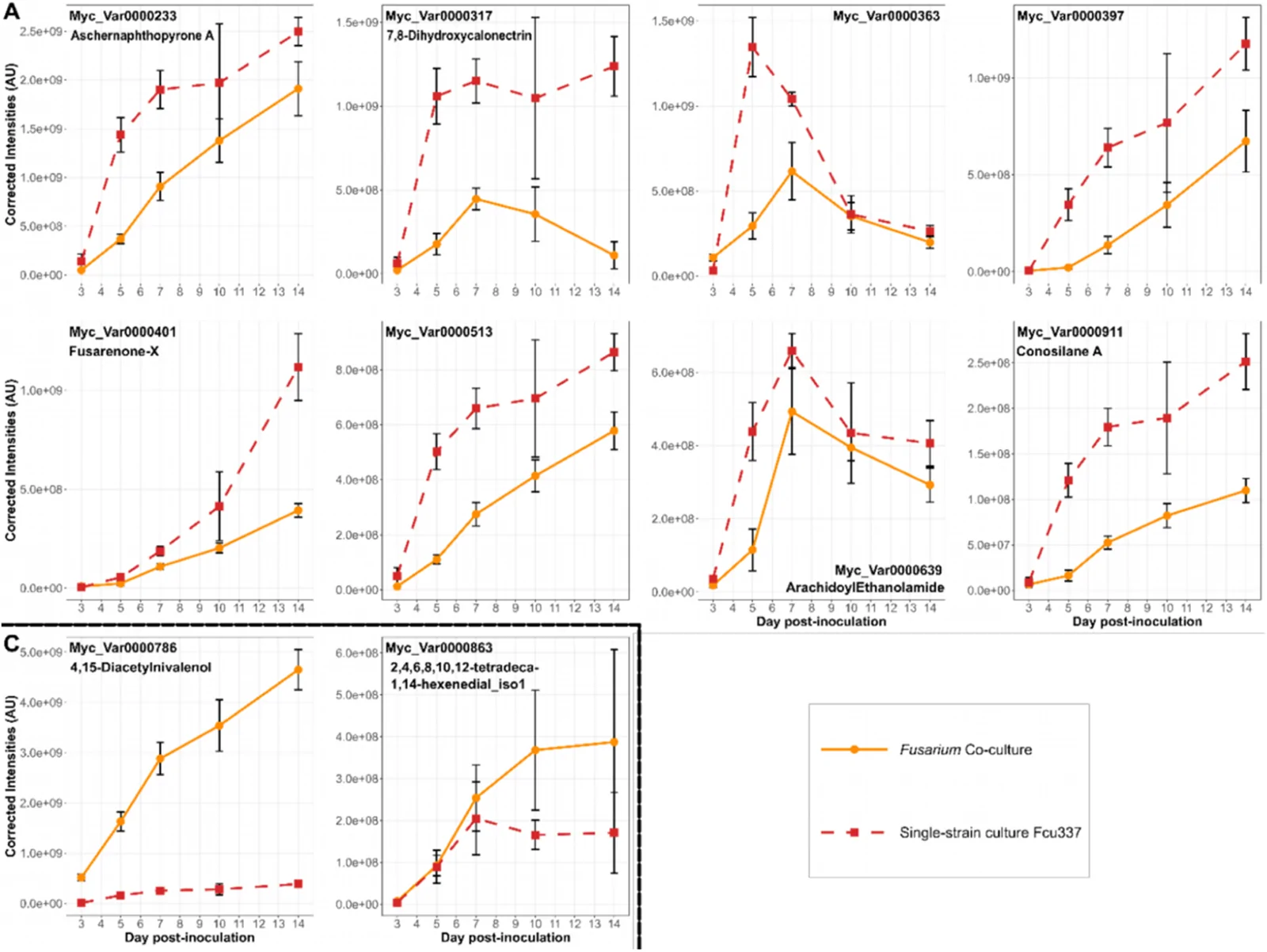
Comparative accumulation dynamics of endometabolic biomarkers of the strain Fcu337 between single-strain and *Fusarium* co-cultures. Biomarker levels were normalized to the average intensity of the internal standard (Methyl Vanillate). Each compound was classified according to its accumulation pattern and differences between both cultures: Group A, similar accumulation pattern with higher intensities in single-strain cultures; Group C, different accumulation patterns in both cultures.

**Fig. 8:**
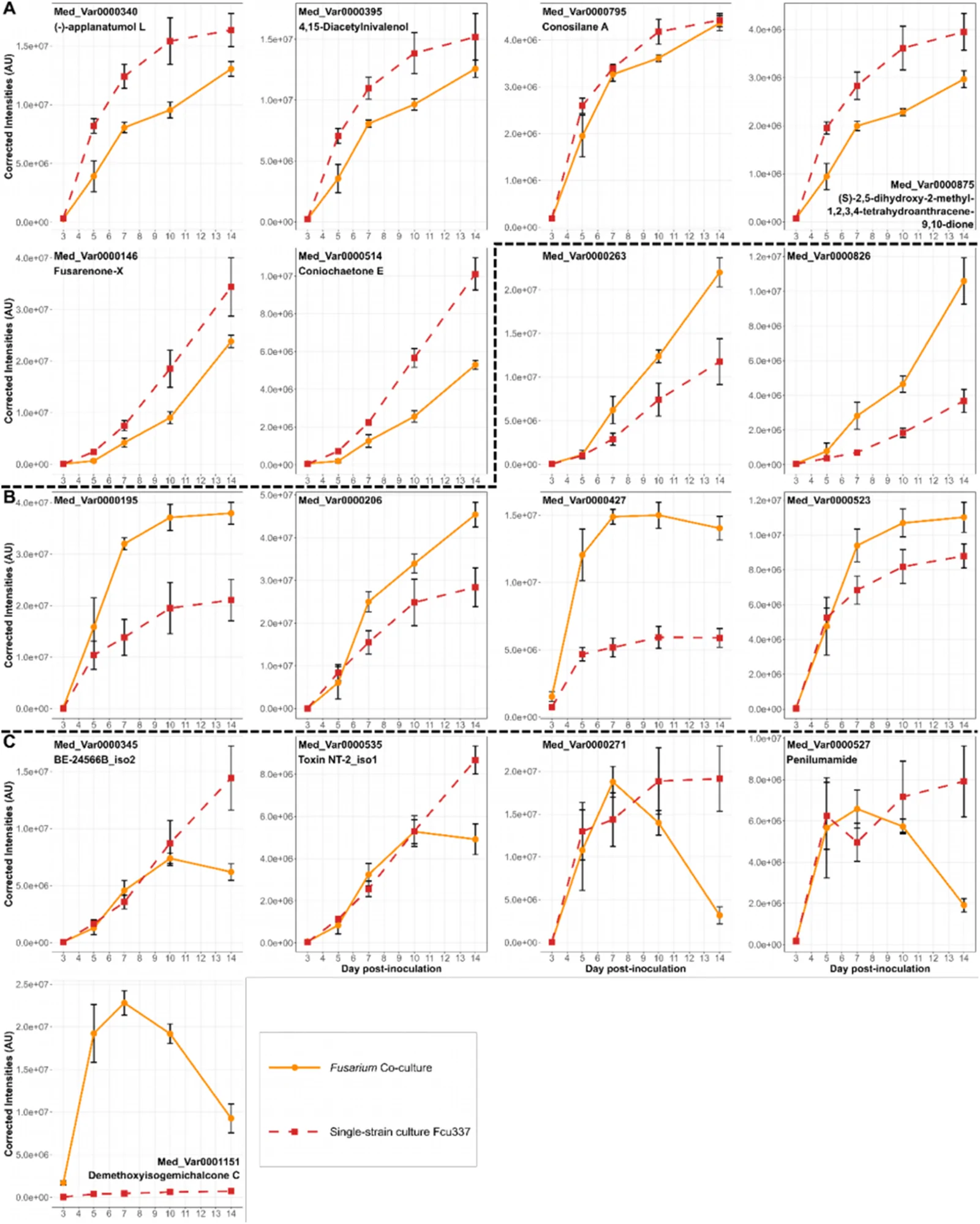
Comparative accumulation dynamics of exometabolomic biomarkers of strain Fcu337 between single-strain cultures and *Fusarium* co-cultures, over the culture period. Biomarker levels were normalized to the average intensity of the internal standard (Methyl Vanillate). Each compound was classified according to its accumulation pattern and differences between both cultures: Group A, similar accumulation pattern with higher intensities in single-strain cultures; Group B, similar accumulation pattern with higher or equivalent intensities in co-cultures; Group C, different accumulation patterns in both cultures.

**Fig. 9:**
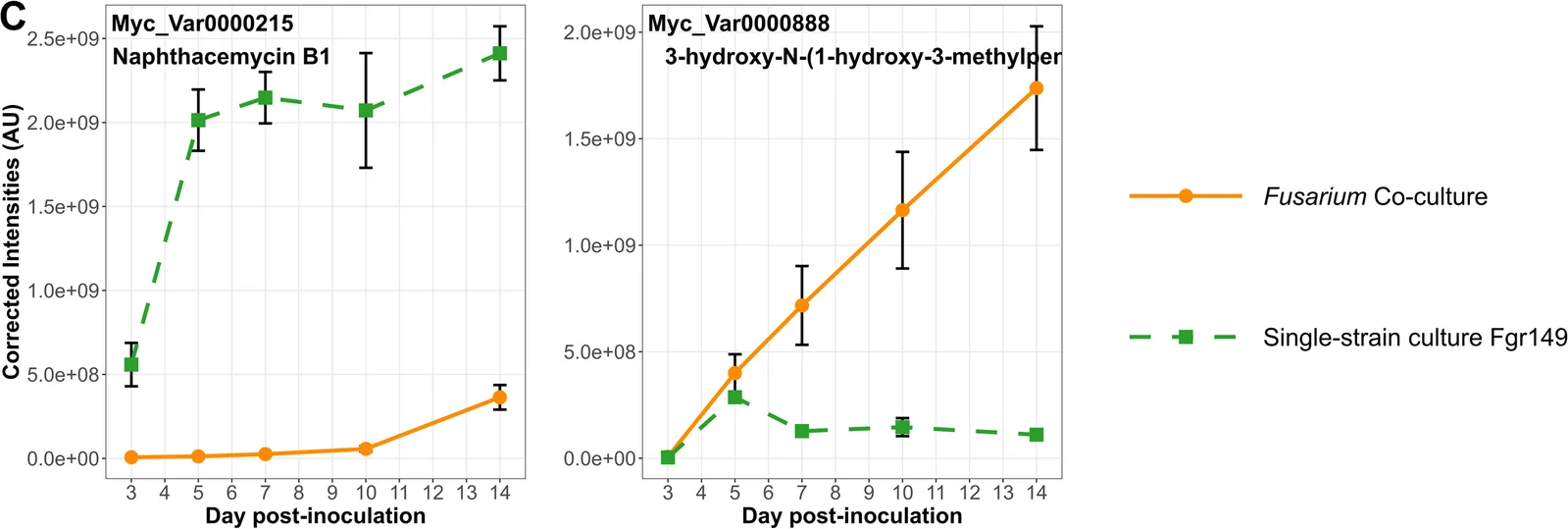
Comparative accumulation dynamics of endometabolomic biomarkers of strain Fgr149 between single-strain cultures and *Fusarium* co-cultures, over the culture period. Biomarker levels were normalized to the average intensity of the internal standard (Methyl Vanillate). Each compound was classified according to its accumulation pattern and differences between both cultures: Group C, different accumulation patterns in both cultures.

**Fig. 10:**
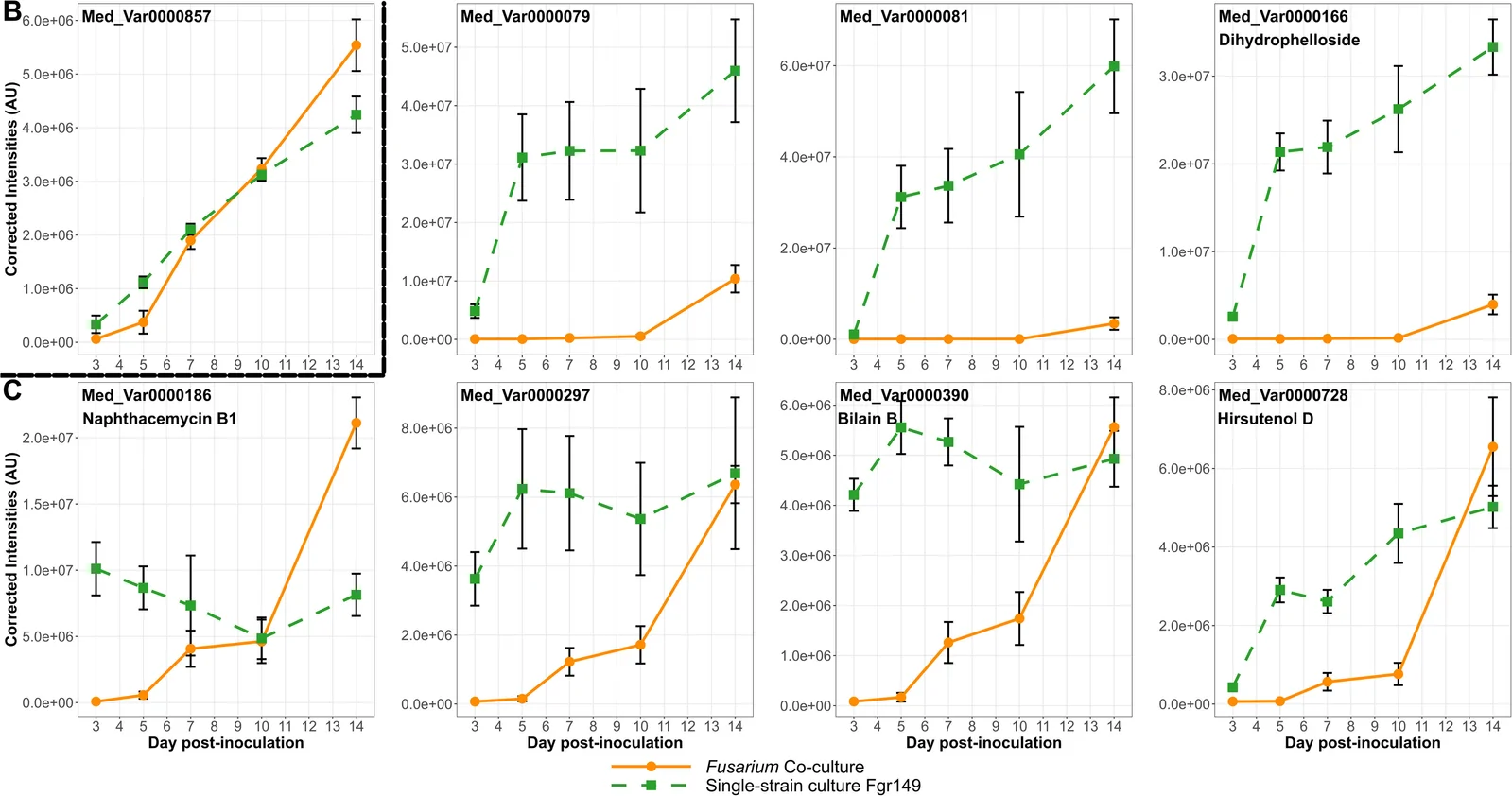
Comparative accumulation dynamics of exometabolomic biomarkers of strain Fgr149 between single-strain cultures and *Fusarium* co-cultures, over the culture period. Biomarker levels were normalized to the average intensity of the internal standard (Methyl Vanillate). Each compound was classified according to its accumulation pattern and differences between both cultures: Group B, similar accumulation pattern with higher or equivalent intensities in co-cultures; Group C, different accumulation patterns in both cultures.

In both mycelium and supernatant extracts, only biomarkers associated with the Fcu337 and Fgr149 strains, previously shown as the two most abundant strains in the *Fusarium* co-cultures (Fig. 6), were detected (Table S4, Table S5). Ten and two biomarkers related to Fcu337 and Fgr149, respectively, were retrieved in the endometabolome, whereas 17 Fcu337 biomarkers and eight Fgr149 biomarkers were detected in the exometabolome of the co-cultures. The previously mentioned 37 biomarkers were monitored over the fungal cultures and compared between single-strain and co-culture experiments. Biomarker peak intensities were corrected using the mean signal of the internal standard (methyl vanillate). The 37 biomarkers were gathered into different groups according to the pattern of their accumulation dynamics: Group A, similar accumulation pattern in both culture types with higher intensities in single-strain cultures; Group B, similar accumulation pattern with higher or equivalent intensities in co-cultures; Group C, different accumulation patterns and intensities in both culture types (Figs. 7, 8, 9, and 10).

##### 3.3.2.1 Comparative accumulation dynamics of biomarkers of the strain Fcu337 in single-strain cultures and co-cultures

Several patterns of accumulation were differentiated for Fcu337 biomarkers retrieved in mycelia. As shown in Fig. 7, eight Fcu337 biomarkers had a similar pattern of accumulation but were generally more abundant in single-strain cultures and were, therefore, gathered in Group A. In this group, 4 compounds (Myc_Var0000233, Myc_Var0000397, Myc_Var0000513, and Myc_Var0000911) showed an increasing accumulation between 3 and 7 dpi, and then slowed down from 7 to 14 dpi. Although their intensities were in the same range, these four biomarkers were generally more abundant in single-strain cultures. A similar trend was observed for the FX mycotoxin (Myc_Var0000401), which showed an exponential accumulation with higher abundance in single-strain cultures (Fig. 7). Two other features (Myc_Var0000363 and Myc_Var0000639) also displayed similar trends between single-strain cultures and the *Fusarium* co-cultures experiment, peaking at 5–7 dpi before declining, with higher intensities in single-strain cultures (Fig. 7). The last feature (Myc_Var0000317) classified in Group A, putatively annotated as a trichothecene precursor, 7,8-Dihydroxycalonectrin (CLI = 3), was shown to accumulate between 3 and 7 dpi before stabilizing, with still higher intensities in single-strain cultures. In contrast, two features exhibited divergent patterns between the two types of cultures and were gathered in Group C (Fig. 7). For both, a higher accumulation was observed in the co-cultures. The first feature (Myc_Var000786) was putatively annotated as a nivalenol precursor, 4,15-Diacetylnivalenol (CLI = 4), with an accumulation characterized by a ∼10-fold higher intensity in the *Fusarium* co-cultures endometabolome (Fig. 7). For the second one (Myc_Var000863), the accumulation continuously increased up to 14 dpi in the co-cultures, reaching an intensity that was twice that observed in Fcu337 single-strain cultures, while it stabilized from 7 dpi in Fcu337 single-strain cultures, although variability was high (Fig. 7).

For exometabolomic biomarkers of Fcu337 (Fig. 8), both similar and contrasting patterns were observed between experiments. Six compounds were classified in Group A (Fig. 8). Four of them (Med_Var0000340, Med_Var0000395, Med_Var0000795, and Med_Var0000875) were characterized by an increasing accumulation between 3 and 7 dpi, that further slowed down from 7 to 14 dpi (Fig. 8). One of these features (Med_Var000395) was also putatively annotated as the nivalenol precursor, 4,15-Diacetylnivalenol (CLI = 4), also previously described as an endometabolite biomarker. Two other features (Med_Var0000146 and Med_Var0000514) from Group A were characterized by a steady and linear accumulation over the growth kinetics (Fig. 8). Among these metabolites, one can mention the FX mycotoxin. Six biomarkers (Med_Var0000263, Med_Var0000826, Med_Var0000195, Med_Var0000206, Med_Var0000427, and Med_Var0000523) were classified in Group B, with similar patterns of accumulation but with higher intensities observed in the *Fusarium* co-cultures (Fig. 8). Lastly, five biomarkers were classified in Group C, with different patterns between both experiments (Fig. 8). For two Fcu337 biomarkers (Med_Var0000345 and Med_Var0000535), with one being putatively annotated as the Toxin NT-2_iso1 (CLI = 2a), their accumulation appeared to stop in the *Fusarium* co-cultures, from 10 to 14 dpi, which was not the case in single-strain cultures (Fig. 8). For two other Fcu337 biomarkers (Med_Var0000271 and Med_Var0000527) classified in Group C, a significant decrease in their abundance was observed starting from 7 dpi in the *Fusarium* co-cultures, while these metabolites were continuously accumulating in single-strain cultures (Fig. 8). A specific pattern of accumulation was also observed for one biomarker (Med_Var0001151), which was 50 times more abundant in the co-cultures and showed a decreasing abundance after 7 days of incubation (Fig. 8).

##### 3.3.2.2 Comparative accumulation dynamics of biomarkers of the strain Fgr149 in single-strain cultures and co-cultures

Only two Fgr149 metabolic biomarkers were identified in the *Fusarium* co-cultures endometabolome. Their dynamics of accumulation were shown to significantly differ from those observed in single-strain cultures and were therefore classified in Group C (Fig. 9). The abundance of one of these biomarkers (Myc_Var0000215) was significantly reduced in the *Fusarium* co-cultures modality, while an inverse trend was observed for the second (Myc_Var0000888), whose abundance at 14 dpi was almost 70 times higher when Fgr149 was co-cultivated with the six other strains compared to single-strain cultures (Fig. 9).

Eight Fgr149 biomarkers were retrieved in the exometabolome of the co-cultures (Fig. 9). According to their accumulation pattern, these metabolites were classified into 2 groups. One Fgr149 biomarker (Med_Var0000857) was classified in Group B, with very close intensities and kinetics of accumulation between the two types of cultures (Fig. 10). The seven others were classified in Group C, their dynamics of accumulation being significantly different between single-strain and co-cultures (Fig. 10). Regarding three features (Med_Var0000079, Med_Var0000081, and Med_Var0000166) from Group C their accumulation displayed a significant increase from 3 to 5 dpi in Fgr149 single-strain cultures before stabilizing, whereas in the *Fusarium* co-cultures*,* this accumulation was delayed and weaker, starting only after 10 dpi. Four biomarkers (Med_Var000186, Med_Var0000297, Med_Var0000390, and Med_Var0000728) that were roughly at a constant level over incubation in single-strain cultures were steadily accumulated in the *Fusarium* co-cultures modality (Fig. 10). One of these four biomarkers, putatively annotated as Naphthacemycin B1 (CLI = 4), was significantly more abundant (2 to 3 fold) at 14 dpi in the *Fusarium* co-cultures supernatant compared to Fgr149 single-strain cultures (Fig. 10).

Altogether, according to the previous data, the kinetics of some biomarkers related to Fcu337 and Fgr149, classified in Group A, were consistent with the strain composition of the *Fusarium* co-cultures, *i.e.*, a reduced amount that could be associated with a reduced relative abundance of the producing strain. For other biomarkers, classified in Group B, their concentration in the *Fusarium* co-cultures was significantly higher than that observed in single-strain cultures, which could reflect an enhancement of their biosynthesis induced by co-occurrence of other *Fusarium* strains. Lastly, for some biomarkers classified in Group C, a significant decrease in their concentration (starting generally from 7 dpi) was observed, which could potentially be ascribed to metabolization, although this has yet to be demonstrated.

## 4 Discussion

FHB is caused by multiple *Fusarium* species that frequently co-infect cereals and produce a wide diversity of secondary metabolites, including mycotoxins (Alisaac and Mahlein, 2023; Ferrigo et al., 2016; Karlsson et al., 2021; Xu and Nicholson, 2009). *Fusarium* co-occurrence during crop infection is acknowledged to significantly modulate disease outcomes and particularly mycotoxin contamination (Karlsson et al., 2021; Wang et al., 2023; Xu and Nicholson, 2009). However, the mechanisms underlying *Fusarium* interactions are still poorly understood, particularly with regard to the contribution of chemical signals and specialized metabolites. As demonstrated by comparative genomic analyses and BGCs investigation, *Fusarium* species causing FHB have a great potential to produce various specialized metabolites (Hoogendoorn et al., 2018; Lin et al., 2023). Studies addressing the issue of specialized metabolites in *Fusarium* species mainly focused on mycotoxins or on virulence factors (Beccari et al., 2020, 2018; Crippin et al., 2019; Thrane et al., 2004; Witte et al., 2024). The present study provides the first comparative metabolomic analysis of seven FHB-associated *Fusarium* strains from distinct species, leading to the identification of a core-metabolome and strain biomarkers. Using *Fusarium* co-cultures characterized for their composition and metabolomic dynamics, this study also provides a first comprehensive methodological framework for future investigation of the chemical dialogue in *Fusarium* interactions. The main findings of our work are (i) a comprehensive database of metabolic features associated with different *Fusarium* strains from distinct species, (ii) the characterization of a core-metabolome and strain biomarkers, and (iii) the identification of strain biomarkers whose accumulation appeared to be modulated by the co-occurrence of multiple *Fusarium* strains.

### 4.1 Differential metabolic activity related to fungal development

The comparative untargeted metabolomics approach used within this study allowed revealing pronounced differences in the metabolic activity of the studied strains. In our cultural conditions, three strains (*F. sporotrichioides* (Fsp101), *F. graminearum* (Fgr149), and *F. culmorum* (Fcu337)) were shown to produce a much broader diversity of intra- and extracellular features than the others (*F. avenaceum* (Fav498), *F. tricinctum* (Ftr521), *F. langsethiae* (Fla509), and *F. poae* (Fpo073)). This ranking is not in accordance with the predicted capacity of production of specialized metabolites of the different *Fusarium* species. Indeed, according to Lin et al. (2023), the genomes of *F. avenaceum* and *F. tricinctum* contain a higher number of predicted BGCs (∼60) compared to the genomes of *F. culmorum, F. graminearum,* and *F. poae* (BGCs number ranging from 41 to 48) (Lin et al., 2023). Considering the reported intraspecific variability of FHB-related species, this discrepancy may stem from the use of only one strain per *Fusarium* species (Garcia et al., 2025). Besides, it is well known that the expression of BGCs is significantly modulated by environmental conditions (Zhgun, 2023; Eagan and Keller, 2025; Mao et al., 2018; Atanasoff-Kardjalieff and Studt, 2022) and we could hypothesize that the MS medium used in the present study was more favorable to the production of specialized metabolites by the strains *F. graminearum, F. culmorum,* and *F. sporotrichioides* compared to the others. In addition, our study also indicated that the strains associated with the highest numbers of metabolites (concentration above the defined thresholds) were also the fast-growing strains producing more biomass. The production of specialized metabolites is known to depend on fungal developmental stage and environmental stimuli: during the trophophase (low metabolic activity, reduced growth, limited or no reproduction), specialized metabolism production is low, whereas during the idiophase (higher metabolic activity, growth, and reproduction), the production of specialized metabolites is fostered (Calvo et al., 2002; Demain, 1986; Gerke et al., 2020; Zhgun, 2023). Accordingly, the link between cellular development and mycotoxin synthesis in *F. graminearum* was documented by the study of Menke et al. (2013) (Menke et al., 2013). More recently, Garcia et al. (2025) confirmed a strong correlation between growth and final mycotoxin production across several FHB-associated *Fusarium* species under diverse environmental conditions, corroborating previous findings (Garcia et al., 2025; Hope et al., 2005; Verheecke-Vaessen et al., 2019).

### 4.2 A core-metabolome and strain biomarkers were characterized

Multivariate analyses showed that endometabolomic and exometabolomic profiles were significantly different across strains. Clustering analysis revealed 3 main groups: (i) Fsp101, Fgr149, and Fcu337, with Fgr149 and Fcu337 closely related, (ii) Fav498 and Ftr521, (iii) Fpo073 and Fla509, which were closer to the second cluster than the first one. The first cluster of Fsp101, Fgr149, and Fcu337 shared the largest proportion of cluster-specific metabolites, with 159 intracellular and 430 extracellular shared features. When annotation was possible, the largest part of these metabolites was putatively annotated as terpenes. Fav498 and Ftr521 also shared a substantial number of features (52 and 61, respectively), most of them being putatively annotated as non-ribosomal peptides, which corroborated previous genomic analyses, *i.e.*, the occurrence of a high number of NRPS in their genome (Lin et al., 2023). In contrast, Fpo073 and Fla509 produced very few cluster-specific metabolites (< 1 %). Interestingly, the previously observed clustering only partially matched with phylogenetic relationships. While data were consistent for the strains of *F. avenaceum* and *F. tricinctum,* which both belong to the FTSC, and for the strains of *F. sporotrichioides, F. graminearum,* and *F. culmorum*, the three former species being included in the FSAMSC (Geiser et al., 2021), the position of strains of *F. langsethiae* and *F. poae* was different. Indeed, the metabolic profiles of the two previous strains, which are members of the FSAMSC, were closer to those of Fav498 and Ftr521 than to those of strains belonging to the same *Fusarium* species complex. In our experimental conditions, strains of *F. langsethiae, F. poae, F. avenaceum,* and *F. tricinctum* were characterized by the lowest metabolic richness, which could also contribute to their metabolic closeness. Nevertheless, many additional rationales could explain the metabolic differences that were observed between strains known to be phylogenetically related. Among these rationales, one can mention independent gene losses, horizontal gene transfer, and formation of superclusters through chromosomal rearrangements, often facilitated by transposable elements (Davière et al., 2001; Gao et al., 2019; Henry et al., 2021; Ma et al., 2010; Tralamazza et al., 2019; Villani et al., 2019; L. Wang et al., 2022). Some of the previous mechanisms were suggested to contribute to the significant variation in BGC repertoires observed within *Fusarium* species complexes (*e.g., Fusarium incarnatum-equiseti* species complex, FGSC) and across species lineages (Hoogendoorn et al., 2018; Lin et al., 2023; Tralamazza et al., 2019; Villani et al., 2019). Metabolic differences can also be ascribed to the fact that many BGCs remain silent under laboratory conditions and require specific stimuli for their expression, mediated through transcriptional and epigenetic regulation (Atanasoff-Kardjalieff and Studt, 2022; Eagan and Keller, 2025; Mao et al., 2018; Xue et al., 2023; Zhgun, 2023).

The present study also allowed the identification of a core-endo- and exometabolome across the seven strains examined, with the core-endometabolome containing the highest number of features. Components of the core-endometabolome were mainly putatively annotated as primary metabolites, corroborating the statement that intracellular metabolites are mostly associated with primary functions, whereas extracellular metabolites are more a reflection of the specialized metabolism in interaction with the environment (Kluger et al., 2015). Search for metabolic biomarkers emphasized some strain differences: Fsp101, Fcu337, Fgr149, and Ftr521, which were previously characterized by the highest metabolomic richness excepted Ftr521, exhibited the largest numbers of biomarkers both at the intracellular and extracellular levels. In contrast, other strains, Fav498, Fpo073, and Fla509 displayed very few biomarkers, both at the intracellular (9, 4, and 5 features, respectively) and extracellular (3, 3, and 2 features, respectively) levels. Among these biomarkers, only a few of them (27) were annotated with a CLI equal to or below 3, meaning at least a partial match of MS spectra. These annotated metabolites included mycotoxins such as DON/15-ADON for Fgr149, FX for Fcu337, and HT2 for Fsp101. Such difficulties in annotation were directly related to the incompleteness of the currently available database dedicated to fungal metabolites.

### 4.3 Biomarker detection linked to strain predominance

In the *Fusarium* co-cultures, Fcu337 was shown to dominate, followed by Fgr149, while the other strains remained at very low DNA relative abundance. Such a result slightly differs from previous studies, where *F. graminearum* was often reported as the most competitive species, followed by *F. culmorum* (Khanal et al., 2024; Siou et al., 2015b, 2015a; Xu et al., 2007). This discrepancy may reflect strain-level variability, since only one isolate per species was used in our study, while intraspecific variability within *Fusarium* species was largely documented (Garcia et al., 2025). The predominance of Fcu337 and Fgr149 may be linked to their faster growth and more active metabolite secretion. Besides, macroconidia production, which is a characteristic of several *Fusarium* species, including *F. graminearum, F. culmorum, F. avenaceum*, and *F. sporotrichioides*, was associated with faster germination and hyphal growth, conferring competitive advantages during early infection stages (Wagacha et al., 2012). In addition, extracellular metabolites can mediate competitive interactions by acting as signaling molecules that modulate fungal metabolism and BGC expression (Eagan and Keller, 2025; Zhgun, 2023).

Only biomarkers from the two dominant strains (Fgr149 and Fcu337) were retrieved in the *Fusarium* co-cultures, which was consistent with the very low relative abundance (< 1 %) of other strains. Similar correlations between DNA abundance and species-specific metabolites were reported by Khanal et al. (2024), where only *F. graminearum* metabolites were detected in co-inoculation experiments involving *F. poae* (Khanal et al., 2024). Overall, a lower number of detectable features, both for endo and exometabolomes, was observed in the *Fusarium* strain co-cultures compared to the total number of features detected in single-strain cultures. By contrast, the report of Chatterjee et al. (2016) indicated a higher metabolite diversity in co-cultures of *Aspergillus niger, Fusarium verticillioides*, and *Clonostachys rosea*, compared to single cultures (Chatterjee et al., 2016) which was hypothetically ascribed to the activation of silent BGCs and induction of the biosynthesis of novel metabolites by strains co-cultivation (Mao et al., 2018; Netzker et al., 2015; Pillay et al., 2022; Wang et al., 2019). In the present study, the temporally separate acquisition and processing of single-strain cultures and the *Fusarium* co-cultures data do not allow the search for novel metabolites induced by co-cultivation.

### 4.4 *Fusarium* interactions induced modifications in strain-specific metabolism

When investigating the dynamics of biomarker accumulation in the *Fusarium* co-cultures, several trends were observed. Firstly, the accumulation of some biomarkers was in accordance with the shifts in the strain composition over time, *i.e.*, a large dominance of Fcu337 in the first stage, followed by an increasing abundance of Fgr149. This was verified for some Fcu337 intracellular biomarkers (including FX) and for extracellular ones, and also for some of the few biomarkers related to Fgr149.

Secondly, the accumulation of some metabolic biomarkers was shown to be influenced by the presence of other *Fusarium* strains. Actually, the concentration of some biomarkers was significantly enhanced in the *Fusarium* co-cultures (sometimes up to 50–70 times more) compared to single-strain cultures. Such an induction could suggest an activation of some biosynthetic pathways induced by the other *Fusarium* strains, as it was widely documented in various microbial communities (Mao et al., 2018; Netzker et al., 2015; Pillay et al., 2022; Wang et al., 2019). However, this metabolic regulation is not directly proven in our study, and it would merit further validation from a functional point of view. Regarding studies that specifically addressed *Fusarium* interactions, one can mention the observed increase in some mycotoxin accumulation induced by co-cultivation of different species (Velluti et al., 2001, 2000; Xu et al., 2007), though results were shown as context-dependent (*e.g.*, isolates, culture media, abiotic and biotic stress) (Cooney et al., 2001; Siou et al., 2015b, 2015a; Wang et al., 2023). Among the putatively annotated features associated with Fcu337 and retrieved in the *Fusarium* co-cultures, the dynamics of FX and its 4,15-diacetylnivalenol (DiANIV) precursor, both detected in mycelium and culture medium, warrant particular attention. Indeed, while FX in the *Fusarium* co-cultures displayed a similar accumulation pattern as observed in single-strain cultures, for both mycelia and culture media, this was not the case for putative DiANIV. The extracellular levels of putative DiANIV were similar between single-strain and co-cultures, but its intracellular levels were approximately 10-fold higher in the *Fusarium* co-cultures compared to single-strain cultures of Fcu337. This elevated intracellular DiANIV that did not appear to affect its extracellular levels or the accumulation of FX could be hypothetically related to an upregulation of the biosynthetic step leading to DiANIV, a metabolic bottleneck, or a dysfunction of metabolite export. This hypothesis requires, however, further validation that must first involve confirmation of the identity of DiANIV and then could benefit from transcriptional approaches.

Thirdly, for some exometabolomic biomarkers of Fcu337, a significant decline in their accumulation pattern occurred at the later stages of the co-cultures, which was not observed in the single-strain cultures, suggesting the potential occurrence of degradation, biotransformation, and/or detoxification events. This explanation is merely a hypothesis that warrants subsequent verification, which could, for instance, use biodegradation tests. One can also not exclude the possible trapping of these molecules within the co-cultures' mycelium. However, no similar compounds (calculated molecular weight and retention time) were retrieved in the endometabolome of the co-cultures. Fungi are known to recycle or detoxify secondary metabolites (Deshmukh et al., 2016; Kumar et al., 2024; Shin et al., 2018; Torres-Farradá et al., 2024), which was also demonstrated for mycotoxins (Kim and Vujanovic, 2017; Liu et al., 2024; Ruiz, 2025). In *Fusarium*, several auto-protective mechanisms against mycotoxins were described, including enzymatic modification (acetylation, glycosylation) (Alexander, 2008; Kimura et al., 1998; McCormick et al., 1999) and active export (Alexander et al., 1999; Wang et al., 2018). Because the dominant strains, measured with DNA relative abundance, in the *Fusarium* co-cultures are closely related, both genetically and metabolically speaking, the occurrence of such mechanisms within the *Fusarium* co-cultures cannot be excluded.

Increasing evidence suggests that the outcome of FHB is not determined solely by the virulence potential of individual *Fusarium* strains but also by chemically mediated interactions among co-occurring fungi, bacteria, and the host plant (Karlsson et al., 2021). In this context, strain-specific metabolite profiles likely reflect ecological traits that enable individual strains to exploit distinct metabolic niches, compete for limited nutrients during spike colonization, and adapt to the dynamic physicochemical conditions encountered throughout infection. The strain-specific biomarkers identified in the present study may therefore provide valuable insights into the ecological mechanisms underlying pathogen establishment, persistence, and disease progression within the wheat spike microbiome. For instance, secondary metabolites produced by *Fusarium* species, including polyketides, non-ribosomal peptides, terpenoids, and siderophores, are known to have putative ecological functions in resource acquisition, oxidative stress tolerance, microbial antagonism, and host manipulation (Amuzu et al., 2024; Huang et al., 2024). Moreover, recent advances in community metabolomics further support the concept that extracellular metabolite profiles represent functional readouts of microbial interactions and can reveal ecological processes that are not captured by genomic analyses alone (Go et al., 2024; Holbrook-Smith et al., 2024). Characterizing the ecological functions of these metabolites through integrated metabolomics, co-culture assays, microbiome analyses, and functional genetics could therefore substantially improve our understanding of how chemical interactions shape FHB pathogenesis and may ultimately facilitate the development of microbiome-informed disease management strategies.

### 4.5 Limitations and future investigation

The present study has allowed evidencing the promising use of comparative metabolomics to uncover metabolic signatures associated with FHB-related *Fusarium* strains and to reveal interaction-driven modulation of specialized metabolism. Actually, insights provided by the present study illustrate how comparative metabolomics could enable the formulation of hypotheses regarding possible metabolic crosstalk between FHB-associated species, in addition to describing metabolomic diversity between strains from different species.

While these results pave the way for promising future research, several aspects deserve further exploration. Firstly, only one isolate per species was included despite the well-known intraspecific variability characterizing FHB-related *Fusarium* species (Garcia et al., 2025). Consequently, all the hypotheses formulated within this study pertain to the strain level and cannot be extrapolated to the species level. Secondly, experiments were conducted under standardized *in vitro* conditions that only partially reflect the ecological complexity of wheat ear environments and may not fully capture the biosynthetic potential of these fungi (Zhgun, 2023). Actually, the complex regulation of specialized metabolites, including those associated with *Fusarium* species, by environmental factors such as the multi-layered network underpinning this regulation has been widely documented (for a recent review, see Amuzu et al., 2024). Similarly, environmental factors have also been shown as driving fungal interactions among *Fusarium* species and significantly modulating their outcomes (Xu et al., 2007).

Lastly, metabolite annotation confidence remained limited for most of the detected features (CLI > 3), as a result of the lack of sufficiently comprehensive and curated databases for fungal metabolites, known to be highly complex and variables between organisms (Adejor et al., 2025; Alves et al., 2025). This limited annotation, using untargeted metabolomics, hampers the possibility of discussing the roles of strain-specific metabolites showing an interesting pattern of accumulation.

Future studies should therefore expand the *Fusarium* strain panel and include various culture media (different nutrient sources, for instance) and culture conditions (such as different temperature, water activity, pH). Future studies should combine multiple metabolomic approaches (*e.g.*, LC–MS, GC–MS, and NMR) with transcriptomic analyses, both to improve the identification of detected features and to determine whether the observed metabolic changes arise from the regulation of biosynthetic pathways or from post-transcriptional mechanisms. Use of isotopic studies, enzymatic analyses and molecular validation experiments could also be useful to functionally validate the hypotheses generated throughout the present study.

## 5 Conclusion

FHB is a devastating fungal disease affecting cereal crops, caused by multiple *Fusarium* species that frequently co-occur and interact during infection. These fungal interactions, acknowledged to significantly impact the disease outcomes, are currently sparsely understood. In the present study, an original experimental approach based on untargeted metabolomics was implemented to compare the metabolic capacity of strains from seven FHB-associated species and demonstrate how co-cultures could modulate metabolic pathways in *Fusarium* strains.

By combining intra- and extracellular metabolomic profiling, we identified a core-metabolome shared across strains from different FHB-related species as well as strain biomarkers. We also evidenced changes in metabolite accumulation patterns within the *Fusarium* co-cultures, compared to single-strain cultures, which allowed to raise several hypotheses (potential down- or upregulation of some biosynthetic pathways, potential occurrence of detoxification or cross-feeding mechanisms) that require, however, to be validated by additional investigations.

Altogether, this work establishes a conceptual and methodological framework to study interactions between *Fusarium* strains through metabolomic profiling of *Fusarium* co-cultures. Completing this approach with genomic and transcriptomic approaches is required to link metabolite-level changes to biosynthetic regulatory networks. Ultimately, deciphering the metabolic interplay within the complex of fungal species causing FHB will advance our understanding of the FHB disease process and contribute to the development of novel strategies to predict or mitigate mycotoxin contamination in cereal crops.

## Data linking

The raw LC–HRMS/MS data, Compound Discoverer processing workflows, feature tables, and associated metadata generated during this study are publicly available in the Research Data Gouv repository (DOI: 10.57745/SJNC38). The repository includes raw mass spectrometry files, processed metabolomic datasets, Compound Discoverer workflows, and R scripts used for statistical analysis and visualization.

## CRediT authorship contribution statement

**Valentin Fiévet:** Conceptualization, Methodology, Formal analysis, Investigation, Data curation, Writing – original draft, Writing – review & editing, Visualization. **Laëtitia Pinson-Gadais:** Conceptualization, Methodology, Resources, Writing – review & editing, Visualization. **Marie-Noëlle Verdal-Bonnin:** Formal analysis. **Maxime Maurin:** Formal analysis. **Stéphane Bernillon:** Conceptualization, Methodology, Resources, Data curation, Writing – review & editing, Visualization, Supervision. **Louis Carles:** Conceptualization, Resources, Data curation, Writing – review & editing, Visualization, Supervision. **Florence Richard-Forget:** Conceptualization, Resources, Writing – review & editing, Visualization, Supervision, Project administration, Funding acquisition.

## Declaration of competing interest

The authors declare the following financial interests/personal relationships which may be considered as potential competing interests:

Florence RICHARD-FORGET reports financial support was provided by French National Research Agency. If there are other authors, they declare that they have no known competing financial interests or personal relationships that could have appeared to influence the work reported in this paper.

## Appendix Supplementary materials

Image, application 1
