# Supplementary material for: Comparative metabolomics of single-strain and co-cultures of seven Fusarium strains causing Fusarium head blight reveals metabolic modifications induced by strain co-occurrence
Source: Curr Res Microb Sci. 2026 Aug 4;11:100657. doi: 10.1016/j.crmicr.2026.100657 (PMC13486378; doi:10.1016/j.crmicr.2026.100657)
Supplement: Supplementary file 1 [file mmc1.docx]

Supplementary material

Additional data and information, including raw MS data, features table returned by Compound Discoverer v3.3, data processing workflows and scripts (R and excel macro), and metadata on samples and variables are available in the Recherche Data Gouv repository (DOI: [10.57745/SJNC38](https://doi.org/10.57745/SJNC38)).

Table S1: Shared metabolites between cluster of Fusarium strains, identified through Upset plot. **Cluster 1** includes features shared by *Fsp101*, *Fgr149*, and *Fcu337*; **Cluster 2** includes *Fav498* and *Ftr521*; **Cluster 3** includes *Fpo073* and *Fla509*. Each feature is described by: an identifier (**Variable_ID**) with a prefix indicating detection in the endometabolome (Myc) or exometabolome (Med); a retention time in minutes (**RT_min**); a mass-to-charge ratio associated with an adduct (**m/z_of_adduct** and **Adduct**); a putative molecular formula and name (**Putative_formula** and **Putative_name**) as assigned by Compound Discoverer software; and a confidence level of identification (Schymanski et al., 2014).

| Cluster | Variable_ID | Type | Putative_formula | Putative_annotation | RT_min | m/z_of_adduct | Adduct | Confidence_level_of_ identification |
| --- | --- | --- | --- | --- | --- | --- | --- | --- |
| (1) | Med_Var0000003 | Exo | C15 H22 O4 | Curvularol_iso1 | 6.955 | 267.15918 | [M+H]+1 | 3 |
| (1) | Med_Var0000027 | Exo | C15 H24 O4 | Trichotriol_iso1 | 6.501 | 269.17501 | [M+H]+1 | 3 |
| (1) | Med_Var0000043 | Exo | C13 H16 N6 O2 | NA | 6.818 | 289.14119 | [M+H]+1 | 4 |
| (1) | Med_Var0000047 | Exo | C19 H28 O6 | Geumsanol C_iso2 | 7.304 | 375.17788 | [M+Na]+1 | 4 |
| (1) | Med_Var0000051 | Exo | C15 H22 O3 | Trichodermol_iso3 | 6.5 | 251.16434 | [M+H]+1 | 3 |
| (1) | Med_Var0000055 | Exo | C21 H24 O8 | Pinophilin E | 5.535 | 405.15244 | [M+H]+1 | 4 |
| (1) | Med_Var0000057 | Exo | C17 H26 O5 | Guignardone I_iso1 | 6.067 | 333.16736 | [M+Na]+1 | 3 |
| (1) | Med_Var0000058 | Exo | C14 H18 O2 | Ochracene F_iso1 | 6.208 | 219.13815 | [M+H]+1 | 4 |
| (1) | Med_Var0000067 | Exo | C15 H20 O3 | Arecolactone_iso1 | 6.206 | 249.1487 | [M+H]+1 | 3 |
| (1) | Med_Var0000068 | Exo | C15 H22 O2 | 12,13-Epoxytrichothec-9-ene_iso1 | 7.241 | 235.1695 | [M+H]+1 | 3 |
| (1) | Med_Var0000069 | Exo | C19 H28 O5 | Alterchothecene B_iso1 | 10.575 | 359.18303 | [M+Na]+1 | 4 |
| (1) | Med_Var0000070 | Exo | C17 H26 O5 | Arundinol C | 7.635 | 333.16743 | [M+Na]+1 | 4 |
| (1) | Med_Var0000075 | Exo | C15 H22 O4 | Curvularol_iso3 | 7.523 | 267.15928 | [M+H]+1 | 3 |
| (1) | Med_Var0000076 | Exo | C19 H28 O6 | Geumsanol C_iso3 | 9.282 | 375.17795 | [M+Na]+1 | 4 |
| (1) | Med_Var0000086 | Exo | C21 H35 N O6 | NA | 7.304 | 398.25382 | [M+H]+1 | 4 |
| (1) | Med_Var0000088 | Exo | C14 H29 P3 | NA | 4.468 | 291.15691 | [M+H]+1 | 4 |
| (1) | Med_Var0000093 | Exo | C15 H24 O4 | Trichotriol_iso3 | 5.518 | 269.17493 | [M+H]+1 | 3 |
| (1) | Med_Var0000105 | Exo | C15 H18 O2 | Meruliolactone_iso1 | 6.21 | 231.13826 | [M+H]+1 | 4 |
| (1) | Med_Var0000107 | Exo | C15 H18 O | 1-Hydroxymethyl-4-methyl-7-isopropenylaz_iso1 | 8.373 | 215.14325 | [M+H]+1 | 4 |
| (1) | Med_Var0000115 | Exo | C15 H28 O4 | Agripilol A_iso1 | 6.355 | 295.18806 | [M+Na]+1 | 4 |
| (1) | Med_Var0000131 | Exo | C15 H28 O4 | Agripilol A_iso2 | 6.183 | 295.18808 | [M+Na]+1 | 4 |
| (1) | Med_Var0000132 | Exo | C15 H22 O4 | Curvularol_iso5 | 5.889 | 267.15928 | [M+H]+1 | 3 |
| (1) | Med_Var0000138 | Exo | C15 H20 O3 | Arecolactone_iso2 | 6.108 | 249.14871 | [M+H]+1 | 3 |
| (1) | Med_Var0000139 | Exo | C9 H10 | NA | 9.123 | 119.0858 | [M+H]+1 | 4 |
| (1) | Med_Var0000143 | Exo | C14 H31 O P3 | NA | 6.953 | 291.15678 | [M+H-H2O]+1 | 4 |
| (1) | Med_Var0000148 | Exo | C15 H22 O2 | 12,13-Epoxytrichothec-9-ene_iso2 | 7.471 | 235.16948 | [M+H]+1 | 3 |
| (1) | Med_Var0000149 | Exo | C15 H25 N O4 | NA | 6.822 | 284.18589 | [M+H]+1 | 4 |
| (1) | Med_Var0000151 | Exo | C15 H20 O3 | Chaetothyrin C | 7.522 | 249.14873 | [M+H]+1 | 3 |
| (1) | Med_Var0000153 | Exo | C15 H22 O5 | Stereumin U | 6.026 | 265.14364 | [M+H-H2O]+1 | 3 |
| (1) | Med_Var0000165 | Exo | C13 H22 N6 O | NA | 7.745 | 279.19324 | [M+H]+1 | 4 |
| (1) | Med_Var0000169 | Exo | C15 H20 O4 | Rhinomilisin H_iso1 | 7.658 | 265.14366 | [M+H]+1 | 4 |
| (1) | Med_Var0000173 | Exo | NA | NA | 7.172 | 413.21075 | [M+H]+1 | 5 |
| (1) | Med_Var0000177 | Exo | C11 H23 N4 O3 P | NA | 7.043 | 291.157 | [M+H]+1 | 4 |
| (1) | Med_Var0000178 | Exo | C15 H22 O | Anaephene A_iso2 | 6.362 | 219.17455 | [M+H]+1 | 3 |
| (1) | Med_Var0000182 | Exo | C47 H68 O5 P4 | NA | 6.959 | 419.20888 | [M+2H]+2 | 4 |
| (1) | Med_Var0000192 | Exo | C11 H14 | NA | 9.124 | 147.11701 | [M+H]+1 | 4 |
| (1) | Med_Var0000199 | Exo | C25 H22 O4 | 5-Hydroxy-8,8-dimethyl-6- [ (1E) -3-methyl-1,3-butadienyl ] -2-phenyl-4H,8H-benzo [ 1,2-b:3,4-b' ] dipyran-4-one_iso1 | 6.955 | 194.08349 | [M+2H]+2 | 4 |
| (1) | Med_Var0000204 | Exo | C21 H35 N O6 | NA | 8.439 | 398.25393 | [M+H]+1 | 4 |
| (1) | Med_Var0000205 | Exo | C13 H22 N6 O | NA | 8.451 | 279.19323 | [M+H]+1 | 4 |
| (1) | Med_Var0000214 | Exo | C25 H26 O5 | Flemiwallichin D | 6.5 | 204.09657 | [M+2H]+2 | 4 |
| (1) | Med_Var0000215 | Exo | C15 H22 O4 | 3?,6?-dihydroxycinnamolide_iso2 | 5.706 | 267.15932 | [M+H]+1 | 3 |
| (1) | Med_Var0000216 | Exo | C24 H28 O10 | Grecoketide A | 8.39 | 239.09046 | [M+2H]+2 | 4 |
| (1) | Med_Var0000218 | Exo | C15 H22 O3 | Trichodermol_iso4 | 7.468 | 251.1644 | [M+H]+1 | 3 |
| (1) | Med_Var0000220 | Exo | C12 H23 N O4 | 3-hydroxy-N-(1-hydroxy-3-methylpentan-2-yl)-5-oxohexanamide_iso3 | 4.377 | 246.17017 | [M+H]+1 | 4 |
| (1) | Med_Var0000221 | Exo | C10 H12 | NA | 9.124 | 133.1014 | [M+H]+1 | 4 |
| (1) | Med_Var0000222 | Exo | C17 H29 N O4 | YM-47515_iso2 | 4.875 | 312.21723 | [M+H]+1 | 4 |
| (1) | Med_Var0000224 | Exo | C15 H24 O | 2-Hydroxytrichodiene_iso2 | 8.323 | 203.17968 | [M+H-H2O]+1 | 3 |
| (1) | Med_Var0000225 | Exo | C20 H31 N O6 S | NA | 7.361 | 414.19489 | [M+H]+1 | 4 |
| (1) | Med_Var0000226 | Exo | C10 H12 O3 | N-Propyl 4-hydroxybenzoate | 5.733 | 181.08618 | [M+H]+1 | 4 |
| (1) | Med_Var0000229 | Exo | C17 H26 O5 | Guignardone I_iso2 | 6.169 | 333.16744 | [M+Na]+1 | 3 |
| (1) | Med_Var0000239 | Exo | C15 H18 O3 | 9a-Hydroxy-3,8a-dimethyl-5-methylene4,4a,5,6,9,9a-hexahydronaphtho[2,3-b]furan-2(8aH)-one_iso1 | 8.279 | 247.13302 | [M+H]+1 | 3 |
| (1) | Med_Var0000243 | Exo | C12 H19 N9 O2 | NA | 4.17 | 322.17419 | [M+H]+1 | 4 |
| (1) | Med_Var0000244 | Exo | C15 H25 N O4 | NA | 5.771 | 284.1859 | [M+H]+1 | 4 |
| (1) | Med_Var0000245 | Exo | C7 H8 O2 | (-)-hepta-4,6-diyne-2,3-diol_iso1 | 6.103 | 125.05995 | [M+H]+1 | 4 |
| (1) | Med_Var0000249 | Exo | C17 H33 N O5 | NA | 6.5 | 332.24344 | [M+H]+1 | 4 |
| (1) | Med_Var0000252 | Exo | C11 H27 N4 O3 P | NA | 6.703 | 295.18816 | [M+H]+1 | 4 |
| (1) | Med_Var0000258 | Exo | C15 H22 O3 | Trichodermol_iso5 | 7.649 | 251.16443 | [M+H]+1 | 3 |
| (1) | Med_Var0000259 | Exo | C8 H8 | Styrene_iso1 | 9.125 | 105.07024 | [M+H]+1 | 4 |
| (1) | Med_Var0000261 | Exo | C17 H26 O4 | Tricholopardin B_iso2 | 8.103 | 295.19057 | [M+H]+1 | 4 |
| (1) | Med_Var0000265 | Exo | NA | NA | 6.691 | 293.17247 | [M+2H]+2 | 5 |
| (1) | Med_Var0000267 | Exo | C15 H18 O | 1-Hydroxymethyl-4-methyl-7-isopropenylaz_iso2 | 7.307 | 215.14329 | [M+H]+1 | 4 |
| (1) | Med_Var0000273 | Exo | C15 H22 O3 | Trichodermol_iso6 | 7.834 | 251.16439 | [M+H]+1 | 3 |
| (1) | Med_Var0000275 | Exo | C15 H22 O3 | Trichodermol_iso7 | 6.768 | 273.14628 | [M+Na]+1 | 3 |
| (1) | Med_Var0000277 | Exo | C15 H24 O | 2-Hydroxytrichodiene_iso3 | 7.762 | 221.19022 | [M+H]+1 | 4 |
| (1) | Med_Var0000280 | Exo | C25 H24 O4 | Paratocarpin A_iso1 | 8.373 | 195.09137 | [M+2H]+2 | 4 |
| (1) | Med_Var0000286 | Exo | C19 H33 N O5 | NA | 6.067 | 356.24344 | [M+H]+1 | 4 |
| (1) | Med_Var0000287 | Exo | C14 H20 O | Fusidione_iso1 | 8.375 | 205.15896 | [M+H]+1 | 4 |
| (1) | Med_Var0000298 | Exo | C13 H20 N6 O3 | NA | 5.542 | 309.16752 | [M+H]+1 | 4 |
| (1) | Med_Var0000305 | Exo | C13 H22 N6 O2 | NA | 5.999 | 295.18817 | [M+H]+1 | 4 |
| (1) | Med_Var0000309 | Exo | C11 H21 N4 O3 P | NA | 5.063 | 289.14127 | [M+H]+1 | 4 |
| (1) | Med_Var0000311 | Exo | C17 H26 O5 | Trichodermarin F_iso1 | 7.446 | 333.16752 | [M+Na]+1 | 3 |
| (1) | Med_Var0000313 | Exo | C11 H27 N4 O2 P | NA | 7.512 | 279.19331 | [M+H]+1 | 4 |
| (1) | Med_Var0000314 | Exo | C15 H24 O | 2-Hydroxytrichodiene_iso4 | 8.454 | 221.19021 | [M+H]+1 | 4 |
| (1) | Med_Var0000317 | Exo | C14 H20 O | Fusidione_iso2 | 5.36 | 237.1851 | [M+H+MeOH]+1 | 4 |
| (1) | Med_Var0000318 | Exo | C15 H24 O2 | 12,13-Epoxy-9,10-trichoene-2-ol_iso3 | 5.36 | 237.18519 | [M+H]+1 | 3 |
| (1) | Med_Var0000320 | Exo | C19 H31 N3 O3 P2 | NA | 8.371 | 206.5993 | [M+2H]+2 | 4 |
| (1) | Med_Var0000321 | Exo | C15 H20 O3 | Arecolactone_iso3 | 7.108 | 271.13068 | [M+Na]+1 | 3 |
| (1) | Med_Var0000325 | Exo | C21 H28 N5 O3 P | NA | 6.861 | 215.60455 | [M+2H]+2 | 4 |
| (1) | Med_Var0000326 | Exo | C15 H24 O4 | Trichotriol_iso5 | 6.672 | 269.17496 | [M+H]+1 | 3 |
| (1) | Med_Var0000329 | Exo | C17 H26 O5 | Trichodermarin F_iso2 | 6.636 | 333.16737 | [M+Na]+1 | 3 |
| (1) | Med_Var0000334 | Exo | C8 H12 O | NA | 5.888 | 125.09634 | [M+H]+1 | 4 |
| (1) | Med_Var0000337 | Exo | C15 H22 O3 | Trichodermol_iso8 | 8.692 | 251.16439 | [M+H]+1 | 3 |
| (1) | Med_Var0000341 | Exo | C25 H22 O4 | 5-Hydroxy-8,8-dimethyl-6- [ (1E) -3-methyl-1,3-butadienyl ] -2-phenyl-4H,8H-benzo [ 1,2-b:3,4-b' ] dipyran-4-one_iso2 | 5.887 | 194.08351 | [M+2H]+2 | 4 |
| (1) | Med_Var0000344 | Exo | C13 H14 N6 O | NA | 7.652 | 271.13068 | [M+H]+1 | 4 |
| (1) | Med_Var0000354 | Exo | C14 H27 P3 | NA | 4.963 | 289.14126 | [M+H]+1 | 4 |
| (1) | Med_Var0000362 | Exo | C15 H22 O3 | Trichodermol_iso9 | 5.583 | 251.16444 | [M+H]+1 | 4 |
| (1) | Med_Var0000366 | Exo | C13 H20 N6 O | NA | 8.207 | 277.17765 | [M+H]+1 | 4 |
| (1) | Med_Var0000371 | Exo | C15 H22 | Rulepidadiene B_iso2 | 7.762 | 203.17971 | [M+H]+1 | 4 |
| (1) | Med_Var0000375 | Exo | C15 H20 O3 | 2-oxoeremophila-1(10),11(13)-dien-12-oic acid | 7.044 | 249.14877 | [M+H]+1 | 3 |
| (1) | Med_Var0000379 | Exo | NA | NA | 3.996 | 371.26563 | [2M+H]+1 | 5 |
| (1) | Med_Var0000380 | Exo | C21 H35 N O5 | NA | 10.575 | 382.25918 | [M+H]+1 | 4 |
| (1) | Med_Var0000381 | Exo | C17 H24 O5 | 15-Decalonectrin_iso1 | 7.614 | 309.16995 | [M+H]+1 | 4 |
| (1) | Med_Var0000383 | Exo | C17 H26 O5 | Trichodermarin F_iso3 | 8.124 | 333.16751 | [M+Na]+1 | 3 |
| (1) | Med_Var0000387 | Exo | C17 H27 N O3 | Pyreudione B_iso1 | 6.208 | 294.20663 | [M+H]+1 | 4 |
| (1) | Med_Var0000389 | Exo | C15 H20 O3 | Arecolactone_iso4 | 8.751 | 249.14875 | [M+H]+1 | 3 |
| (1) | Med_Var0000391 | Exo | C15 H20 N6 O3 | NA | 6.244 | 333.16759 | [M+H]+1 | 4 |
| (1) | Med_Var0000392 | Exo | C15 H31 O4 P3 | NA | 6.206 | 185.07818 | [M+2H]+2 | 4 |
| (1) | Med_Var0000396 | Exo | C15 H24 O2 | 12,13-Epoxy-9,10-trichoene-2-ol_iso4 | 5.46 | 237.18514 | [M+H]+1 | 3 |
| (1) | Med_Var0000399 | Exo | C17 H26 O5 | Penisporolide B | 7.17 | 311.18559 | [M+H]+1 | 3 |
| (1) | Med_Var0000400 | Exo | NA | NA | 6.956 | 306.64612 | [M+2H]+2 | 5 |
| (1) | Med_Var0000401 | Exo | C15 H18 N4 O4 S | NA | 6.959 | 392.13849 | [M+ACN+H]+1 | 4 |
| (1) | Med_Var0000402 | Exo | C15 H20 O4 | Trichodermaloid C | 5.003 | 265.14372 | [M+H]+1 | 3 |
| (1) | Med_Var0000405 | Exo | C27 H24 O7 | BE-24566B_iso2 | 4.382 | 231.08371 | [M+2H]+2 | 4 |
| (1) | Med_Var0000407 | Exo | C15 H18 | Guaiazulene_iso1 | 7.243 | 199.14844 | [M+H]+1 | 4 |
| (1) | Med_Var0000409 | Exo | C15 H24 O2 | 12,13-Epoxy-9,10-trichoene-2-ol_iso5 | 5.823 | 237.18516 | [M+H]+1 | 3 |
| (1) | Med_Var0000410 | Exo | C15 H26 O3 | Isocadinanol A | 5.819 | 237.18525 | [M+H-H2O]+1 | 4 |
| (1) | Med_Var0000411 | Exo | C15 H24 O | 2-Hydroxytrichodiene_iso5 | 7.514 | 221.19026 | [M+H]+1 | 4 |
| (1) | Med_Var0000415 | Exo | C32 H42 O8 | 11?-hydroxycolossolactone VIII_iso1 | 5.77 | 555.29322 | [M+H]+1 | 4 |
| (1) | Med_Var0000418 | Exo | C13 H22 N6 O2 | NA | 6.558 | 295.18829 | [M+H]+1 | 4 |
| (1) | Med_Var0000419 | Exo | C6 H8 | NA | 7.242 | 161.13272 | [2M+H]+1 | 4 |
| (1) | Med_Var0000423 | Exo | C21 H26 N5 O3 P | NA | 7.522 | 214.59674 | [M+2H]+2 | 4 |
| (1) | Med_Var0000428 | Exo | NA | NA | 6.115 | 307.15182 | [M+2H]+2 | 5 |
| (1) | Med_Var0000430 | Exo | C14 H17 N4 O2 P | NA | 6.822 | 305.11525 | [M+H]+1 | 4 |
| (1) | Med_Var0000435 | Exo | C15 H25 N O4 | NA | 7.286 | 284.18596 | [M+H]+1 | 4 |
| (1) | Med_Var0000436 | Exo | C13 H18 N6 O3 | Lupinate_iso1 | 6.114 | 307.15191 | [M+H]+1 | 4 |
| (1) | Med_Var0000437 | Exo | C14 H20 O2 | Benquoine | 6.863 | 221.15387 | [M+H]+1 | 3 |
| (1) | Med_Var0000441 | Exo | C15 H18 O | 1-Hydroxymethyl-4-methyl-7-isopropenylaz_iso3 | 6.07 | 215.14328 | [M+H]+1 | 4 |
| (1) | Med_Var0000444 | Exo | C13 H24 N6 O3 | Ficellomycin_iso1 | 3.564 | 313.19878 | [M+H]+1 | 4 |
| (1) | Med_Var0000446 | Exo | C15 H18 O2 | Lorneic acid J | 5.886 | 231.13824 | [M+H]+1 | 4 |
| (1) | Med_Var0000447 | Exo | C15 H18 O | 1-Hydroxymethyl-4-methyl-7-isopropenylaz_iso4 | 6.5 | 215.14331 | [M+H]+1 | 4 |
| (1) | Med_Var0000448 | Exo | C30 H51 N O6 P2 | NA | 7.242 | 292.66691 | [M+2H]+2 | 4 |
| (1) | Med_Var0000450 | Exo | C32 H42 O8 | 11?-hydroxycolossolactone VIII_iso2 | 6.822 | 555.29327 | [M+H]+1 | 4 |
| (1) | Med_Var0000451 | Exo | C14 H14 O | NA | 7.525 | 231.13824 | [M+H+MeOH]+1 | 4 |
| (1) | Med_Var0000453 | Exo | C15 H22 O5 | Piltunine A_iso1 | 5.65 | 283.15428 | [M+H]+1 | 3 |
| (1) | Med_Var0000454 | Exo | NA | NA | 7.537 | 411.19526 | [M+H]+1 | 5 |
| (1) | Med_Var0000458 | Exo | C17 H24 O4 | Isotrichodermin_iso1 | 8.12 | 293.17498 | [M+H]+1 | 4 |
| (1) | Med_Var0000461 | Exo | C17 H27 N O3 | Pyreudione B_iso2 | 7.106 | 294.2066 | [M+H]+1 | 4 |
| (1) | Med_Var0000463 | Exo | C15 H20 O2 | Sch 725681_iso2 | 6.131 | 233.15388 | [M+H]+1 | 4 |
| (1) | Med_Var0000467 | Exo | C15 H20 O3 | Arecolactone_iso5 | 5.706 | 249.14879 | [M+H]+1 | 3 |
| (1) | Med_Var0000468 | Exo | C25 H22 O4 | 5-Hydroxy-8,8-dimethyl-6- [ (1E) -3-methyl-1,3-butadienyl ] -2-phenyl-4H,8H-benzo [ 1,2-b:3,4-b' ] dipyran-4-one_iso3 | 7.153 | 194.08352 | [M+2H]+2 | 4 |
| (1) | Med_Var0000471 | Exo | C14 H18 O | Penidienone_iso1 | 8.371 | 203.14333 | [M+H]+1 | 4 |
| (1) | Med_Var0000472 | Exo | C15 H20 O | Anaephene C_iso3 | 7.868 | 217.15894 | [M+H]+1 | 4 |
| (1) | Med_Var0000477 | Exo | C14 H26 O5 P2 | NA | 7.242 | 378.15925 | [M+ACN+H]+1 | 4 |
| (1) | Med_Var0000478 | Exo | C18 H24 N3 O4 P | NA | 7.241 | 378.15918 | [M+H]+1 | 4 |
| (1) | Med_Var0000481 | Exo | C15 H20 O3 | Arecolactone_iso6 | 5.772 | 249.14877 | [M+H]+1 | 3 |
| (1) | Med_Var0000482 | Exo | C7 H10 O2 | 5-Hydroxy-2,3-dimethylcyclopent-2-enone_iso1 | 8.373 | 127.0756 | [M+H]+1 | 4 |
| (1) | Med_Var0000487 | Exo | C15 H20 O2 | Sch 725681_iso3 | 10.348 | 233.15386 | [M+H]+1 | 4 |
| (1) | Med_Var0000491 | Exo | C13 H22 N6 O2 | NA | 5.56 | 295.18827 | [M+H]+1 | 4 |
| (1) | Med_Var0000493 | Exo | C25 H24 O5 | Glyinflanin G_iso1 | 6.965 | 203.08879 | [M+2H]+2 | 4 |
| (1) | Med_Var0000494 | Exo | C13 H16 N6 O | NA | 8.676 | 273.14638 | [M+H]+1 | 4 |
| (1) | Med_Var0000498 | Exo | C7 H8 O2 | (-)-hepta-4,6-diyne-2,3-diol_iso2 | 5.887 | 125.05995 | [M+H]+1 | 4 |
| (1) | Med_Var0000501 | Exo | C15 H24 O5 | Zosteropenilline L | 5.63 | 285.16991 | [M+H]+1 | 3 |
| (1) | Med_Var0000503 | Exo | C14 H18 N6 O2 | NA | 8.75 | 303.15714 | [M+H]+1 | 4 |
| (1) | Med_Var0000510 | Exo | C15 H22 O | Anaephene A_iso5 | 6.597 | 219.17459 | [M+H]+1 | 3 |
| (1) | Med_Var0000511 | Exo | C15 H20 O2 | Sch 725681_iso4 | 6.67 | 233.15388 | [M+H]+1 | 4 |
| (1) | Med_Var0000512 | Exo | C13 H16 N6 O | NA | 6.555 | 273.14639 | [M+H]+1 | 4 |
| (1) | Med_Var0000517 | Exo | C14 H18 O | Penidienone_iso2 | 6.555 | 203.14332 | [M+H]+1 | 4 |
| (1) | Med_Var0000522 | Exo | C27 H27 N O3 | Shearilicine_iso5 | 6.698 | 207.60715 | [M+2H]+2 | 4 |
| (1) | Med_Var0000525 | Exo | C22 H30 N6 O2 P2 | NA | 7.305 | 237.10188 | [M+2H]+2 | 4 |
| (1) | Med_Var0000531 | Exo | C17 H26 O5 | Trichodermarin F_iso4 | 6.917 | 333.16759 | [M+Na]+1 | 3 |
| (1) | Med_Var0000532 | Exo | C15 H18 O3 | 9a-Hydroxy-3,8a-dimethyl-5-methylene4,4a,5,6,9,9a-hexahydronaphtho[2,3-b]furan-2(8aH)-one_iso2 | 6.884 | 247.13316 | [M+H]+1 | 3 |
| (1) | Med_Var0000536 | Exo | C33 H36 N4 O8 | RP-66453 | 6.959 | 617.26351 | [M+H]+1 | 4 |
| (1) | Med_Var0000537 | Exo | NA | NA | 6.958 | 617.26338 | [2M+NH4]+1 | 5 |
| (1) | Med_Var0000538 | Exo | C9 H12 O | 2-Phenylpropan-2-ol | 6.209 | 137.09628 | [M+H]+1 | 4 |
| (1) | Med_Var0000542 | Exo | C15 H20 O3 | Arecolactone_iso7 | 5.314 | 249.14882 | [M+H]+1 | 3 |
| (1) | Med_Var0000545 | Exo | C15 H22 O | Anaephene A_iso6 | 6.7 | 219.17461 | [M+H]+1 | 3 |
| (1) | Med_Var0000546 | Exo | C15 H22 O5 | Piltunine A_iso2 | 5.012 | 305.13624 | [M+Na]+1 | 4 |
| (1) | Med_Var0000548 | Exo | C15 H24 O4 | Trichotriol_iso6 | 5.849 | 291.15681 | [M+Na]+1 | 4 |
| (1) | Med_Var0000551 | Exo | C13 H18 N6 O3 | Lupinate_iso2 | 6.398 | 307.15185 | [M+H]+1 | 4 |
| (1) | Med_Var0000554 | Exo | C15 H22 N6 O4 | NA | 7.449 | 351.17817 | [M+H]+1 | 4 |
| (1) | Med_Var0000563 | Exo | C15 H20 O2 | Sch 725681_iso5 | 6.069 | 233.15386 | [M+H]+1 | 4 |
| (1) | Med_Var0000567 | Exo | C15 H24 O2 | 12,13-Epoxy-9,10-trichoene-2-ol_iso8 | 5.693 | 237.1852 | [M+H]+1 | 3 |
| (1) | Med_Var0000570 | Exo | C17 H31 N3 O4 | Diprotin A_iso2 | 5.468 | 342.23906 | [M+H]+1 | 4 |
| (1) | Med_Var0000571 | Exo | C15 H26 O4 | 12-Hydroxy-6-epi-albrassitriol | 7.353 | 293.1726 | [M+Na]+1 | 3 |
| (1) | Med_Var0000574 | Exo | C15 H24 O4 | Trichotriol_iso7 | 5.417 | 269.17501 | [M+H]+1 | 3 |
| (1) | Med_Var0000578 | Exo | C15 H28 O4 | Agripilol A_iso3 | 5.872 | 295.1881 | [M+Na]+1 | 4 |
| (1) | Med_Var0000581 | Exo | C17 H24 O5 | 15-Decalonectrin_iso3 | 8.133 | 309.16994 | [M+H]+1 | 4 |
| (1) | Med_Var0000582 | Exo | C13 H24 N6 O3 | Ficellomycin_iso2 | 2.264 | 313.19881 | [M+H]+1 | 4 |
| (1) | Med_Var0000585 | Exo | C10 H14 O | (-)-Isopiperitenone_iso1 | 6.183 | 133.10141 | [M+H-H2O]+1 | 4 |
| (1) | Med_Var0000587 | Exo | C14 H20 N6 O3 | NA | 7.884 | 321.16753 | [M+H]+1 | 4 |
| (1) | Med_Var0000590 | Exo | C25 H26 O4 | Lespeol_iso1 | 7.244 | 196.09922 | [M+2H]+2 | 4 |
| (1) | Med_Var0000592 | Exo | C15 H18 O3 | Nigriterpene B | 5.651 | 247.13313 | [M+H]+1 | 4 |
| (1) | Med_Var0000596 | Exo | C16 H39 N4 O5 P | NA | 4.403 | 399.27189 | [M+H]+1 | 4 |
| (1) | Med_Var0000597 | Exo | C18 H22 N3 O4 P | NA | 8.221 | 376.14354 | [M+H]+1 | 4 |
| (1) | Med_Var0000598 | Exo | C13 H20 N6 O | NA | 8.846 | 277.17768 | [M+H]+1 | 4 |
| (1) | Med_Var0000599 | Exo | C25 H22 O4 | 5-Hydroxy-8,8-dimethyl-6- [ (1E) -3-methyl-1,3-butadienyl ] -2-phenyl-4H,8H-benzo [ 1,2-b:3,4-b' ] dipyran-4-one_iso4 | 7.522 | 194.08352 | [M+2H]+2 | 4 |
| (1) | Med_Var0000602 | Exo | C11 H14 O | Valerophenone,1-phenylpentan-1-one_iso1 | 8.367 | 145.10139 | [M+H-H2O]+1 | 4 |
| (1) | Med_Var0000605 | Exo | C13 H22 N6 O2 | NA | 6.267 | 295.18837 | [M+H]+1 | 4 |
| (1) | Med_Var0000607 | Exo | C13 H20 N6 O | NA | 7.697 | 277.17774 | [M+H]+1 | 4 |
| (1) | Med_Var0000610 | Exo | C15 H20 O2 | Sch 725681_iso6 | 6.248 | 233.15388 | [M+H]+1 | 4 |
| (1) | Med_Var0000612 | Exo | C17 H27 N O5 | NA | 9.493 | 326.19655 | [M+H]+1 | 4 |
| (1) | Med_Var0000613 | Exo | C15 H20 O | Anaephene C_iso4 | 6.694 | 217.15897 | [M+H]+1 | 4 |
| (1) | Med_Var0000619 | Exo | C13 H20 N6 O2 | NA | 6.824 | 293.17258 | [M+H]+1 | 4 |
| (1) | Med_Var0000621 | Exo | C14 H18 O | Penidienone_iso3 | 7.639 | 203.14337 | [M+H]+1 | 4 |
| (1) | Med_Var0000622 | Exo | C12 H16 | NA | 7.465 | 161.1327 | [M+H]+1 | 4 |
| (1) | Med_Var0000631 | Exo | C13 H22 N6 O | NA | 8.055 | 279.19334 | [M+H]+1 | 4 |
| (1) | Med_Var0000632 | Exo | C15 H20 O4 | Rhinomilisin H_iso2 | 5.648 | 265.14375 | [M+H]+1 | 3 |
| (1) | Med_Var0000633 | Exo | C17 H22 N6 O4 | NA | 7.535 | 375.1782 | [M+H]+1 | 4 |
| (1) | Med_Var0000636 | Exo | C25 H22 O3 | Maculalactone L_iso2 | 8.371 | 186.08596 | [M+2H]+2 | 4 |
| (1) | Med_Var0000638 | Exo | C15 H16 O | Zafrin_iso1 | 8.232 | 213.12769 | [M+H]+1 | 4 |
| (1) | Med_Var0000640 | Exo | C21 H26 N5 O2 P | NA | 9.646 | 206.5993 | [M+2H]+2 | 4 |
| (1) | Med_Var0000641 | Exo | C13 H16 | NA | 8.372 | 173.13273 | [M+H]+1 | 4 |
| (1) | Med_Var0000642 | Exo | C15 H18 O2 | 10-Oxodehydrodihydrobotrydial | 6.962 | 231.13823 | [M+H]+1 | 4 |
| (1) | Med_Var0000651 | Exo | C15 H24 O4 | Trichotriol_iso8 | 6.206 | 269.17505 | [M+H]+1 | 4 |
| (1) | Med_Var0000657 | Exo | C15 H18 | Guaiazulene_iso2 | 7.467 | 199.14844 | [M+H]+1 | 4 |
| (1) | Med_Var0000659 | Exo | C25 H24 O4 | Paratocarpin A_iso2 | 6.118 | 195.09136 | [M+2H]+2 | 4 |
| (1) | Med_Var0000663 | Exo | C17 H26 O4 | Tricholopardin B_iso3 | 7.869 | 295.1907 | [M+H]+1 | 4 |
| (1) | Med_Var0000666 | Exo | C15 H26 O4 | Diaporol B | 6.689 | 253.18013 | [M+H-H2O]+1 | 3 |
| (1) | Med_Var0000667 | Exo | C15 H24 O3 | Isotrichodiol_iso5 | 6.695 | 253.18012 | [M+H]+1 | 3 |
| (1) | Med_Var0000669 | Exo | C8 H10 | NA | 8.373 | 107.08587 | [M+H]+1 | 4 |
| (1) | Med_Var0000670 | Exo | C13 H18 N6 O2 | NA | 5.583 | 291.15698 | [M+H]+1 | 4 |
| (1) | Med_Var0000671 | Exo | C15 H24 O2 | 12,13-Epoxy-9,10-trichoene-2-ol_iso9 | 3.56 | 237.18516 | [M+H]+1 | 3 |
| (1) | Med_Var0000672 | Exo | C16 H21 N2 O4 P | NA | 7.464 | 378.15921 | [M+ACN+H]+1 | 4 |
| (1) | Med_Var0000674 | Exo | C13 H16 N6 O2 | NA | 5.314 | 289.14135 | [M+H]+1 | 4 |
| (1) | Med_Var0000680 | Exo | C17 H24 O4 | Isotrichodermin_iso2 | 7.304 | 293.17502 | [M+H]+1 | 4 |
| (1) | Med_Var0000682 | Exo | C8 H12 O | NA | 6.103 | 125.09634 | [M+H]+1 | 4 |
| (1) | Med_Var0000686 | Exo | C25 H24 O4 | Paratocarpin A_iso3 | 5.514 | 195.09134 | [M+2H]+2 | 4 |
| (1) | Med_Var0000687 | Exo | C11 H14 O | Valerophenone,1-phenylpentan-1-one_iso2 | 6.362 | 163.11198 | [M+H]+1 | 4 |
| (1) | Med_Var0000689 | Exo | C19 H33 N3 O4 P2 | NA | 6.672 | 215.60456 | [M+2H]+2 | 4 |
| (1) | Med_Var0000692 | Exo | C15 H20 O | Anaephene C_iso5 | 10.056 | 217.15897 | [M+H]+1 | 4 |
| (1) | Med_Var0000694 | Exo | C13 H22 N6 O2 | NA | 5.818 | 295.18829 | [M+H]+1 | 4 |
| (1) | Med_Var0000695 | Exo | C15 H24 O2 | 12,13-Epoxy-9,10-trichoene-2-ol_iso10 | 8.735 | 237.18516 | [M+H]+1 | 3 |
| (1) | Med_Var0000697 | Exo | C13 H22 N6 O2 | NA | 5.276 | 295.1883 | [M+H]+1 | 4 |
| (1) | Med_Var0000700 | Exo | C15 H26 O3 | (1S,2R)-3-oxo-2-pentylcyclopentane-1-butyric acid methyl ester | 6.356 | 255.19578 | [M+H]+1 | 3 |
| (1) | Med_Var0000701 | Exo | C17 H29 N O4 | YM-47515_iso4 | 4.964 | 312.2172 | [M+H]+1 | 4 |
| (1) | Med_Var0000702 | Exo | C21 H26 N5 O2 P | NA | 7.171 | 206.59934 | [M+2H]+2 | 4 |
| (1) | Med_Var0000708 | Exo | C17 H24 O4 | Isotrichodermin_iso3 | 7.866 | 293.17508 | [M+H]+1 | 4 |
| (1) | Med_Var0000711 | Exo | C25 H24 O4 | Paratocarpin A_iso4 | 4.985 | 195.09136 | [M+2H]+2 | 4 |
| (1) | Med_Var0000712 | Exo | C21 H28 O9 | 3-Acetylneosolaniol | 5.655 | 425.17871 | [M+H]+1 | 4 |
| (1) | Med_Var0000720 | Exo | C25 H24 O5 | Glyinflanin G_iso2 | 5.891 | 203.08878 | [M+2H]+2 | 4 |
| (1) | Med_Var0000729 | Exo | C15 H22 O5 | Dihydrophaseicacid | 4.287 | 305.1362 | [M+Na]+1 | 3 |
| (1) | Med_Var0000730 | Exo | C15 H18 O | 1-Hydroxymethyl-4-methyl-7-isopropenylaz_iso5 | 6.86 | 215.14333 | [M+H]+1 | 4 |
| (1) | Med_Var0000731 | Exo | C10 H18 O4 | Sebacicacid | 4.68 | 427.23207 | [2M+Na]+1 | 4 |
| (1) | Med_Var0000733 | Exo | C15 H20 O2 | Sch 725681_iso7 | 4.992 | 233.15392 | [M+H]+1 | 4 |
| (1) | Med_Var0000734 | Exo | C18 H33 O2 P3 | NA | 8.736 | 375.17819 | [M+H]+1 | 4 |
| (1) | Med_Var0000737 | Exo | C20 H29 N O6 S | NA | 6.124 | 394.16844 | [M+H-H2O]+1 | 4 |
| (1) | Med_Var0000740 | Exo | C15 H20 O | Anaephene C_iso6 | 9.735 | 217.15896 | [M+H]+1 | 4 |
| (1) | Med_Var0000745 | Exo | C17 H24 O5 | 15-Decalonectrin_iso4 | 6.683 | 309.16997 | [M+H]+1 | 4 |
| (1) | Med_Var0000748 | Exo | C15 H20 O | Anaephene C_iso7 | 6.137 | 217.15896 | [M+H]+1 | 4 |
| (1) | Med_Var0000749 | Exo | C23 H20 N2 O2 | Epurpurin B_iso1 | 5.276 | 357.15885 | [M+H]+1 | 4 |
| (1) | Med_Var0000750 | Exo | C15 H18 O3 | alpha-Santonin | 7.498 | 247.13321 | [M+H]+1 | 4 |
| (1) | Med_Var0000752 | Exo | C15 H20 N6 O3 | NA | 7.981 | 333.16756 | [M+H]+1 | 4 |
| (1) | Med_Var0000753 | Exo | C18 H20 N6 O S | NA | 6.96 | 185.07815 | [M+2H]+2 | 4 |
| (1) | Med_Var0000754 | Exo | C17 H27 N O3 | Pyreudione B_iso3 | 7.655 | 294.2067 | [M+H]+1 | 4 |
| (1) | Med_Var0000758 | Exo | C12 H14 | NA | 7.242 | 159.11706 | [M+H]+1 | 4 |
| (1) | Med_Var0000759 | Exo | C14 H20 N6 O2 | Discadenine | 8.184 | 305.17262 | [M+H]+1 | 4 |
| (1) | Med_Var0000760 | Exo | C14 H20 O | Fusidione_iso3 | 6.5 | 205.15897 | [M+H]+1 | 4 |
| (1) | Med_Var0000761 | Exo | C7 H10 O | 2,3,5-Trimethylfuran_iso1 | 7.252 | 111.08077 | [M+H]+1 | 4 |
| (1) | Med_Var0000762 | Exo | C47 H67 Cl N2 O4 P2 | NA | 6.959 | 411.22063 | [M+2H]+2 | 4 |
| (1) | Med_Var0000764 | Exo | C17 H26 O6 | (+)-6-O-demethylpestalotiopsin A | 4.144 | 327.18067 | [M+H]+1 | 4 |
| (1) | Med_Var0000765 | Exo | C16 H28 N4 O5 | NA | 5.107 | 357.21368 | [M+H]+1 | 4 |
| (1) | Med_Var0000773 | Exo | C15 H20 O2 | Sch 725681_iso8 | 4.472 | 233.15389 | [M+H]+1 | 4 |
| (1) | Med_Var0000774 | Exo | C15 H20 O | Anaephene C_iso8 | 10.576 | 217.15897 | [M+H]+1 | 4 |
| (1) | Med_Var0000779 | Exo | NA | NA | 6.501 | 412.16455 | [2M+Na]+1 | 5 |
| (1) | Med_Var0000781 | Exo | C11 H14 O3 | (3S)-6-hydroxy-8-methoxy-3-methylisochroman | 8.373 | 195.10179 | [M+H]+1 | 4 |
| (1) | Med_Var0000784 | Exo | C25 H26 O5 | Paratocarpin F | 6.862 | 204.09664 | [M+2H]+2 | 4 |
| (1) | Med_Var0000786 | Exo | C13 H16 N6 O2 | NA | 5.41 | 289.14135 | [M+H]+1 | 4 |
| (1) | Med_Var0000789 | Exo | C17 H30 N2 O3 P2 | NA | 7.464 | 187.09381 | [M+2H]+2 | 4 |
| (1) | Med_Var0000790 | Exo | C15 H27 N O3 | NA | 8.131 | 270.20658 | [M+H]+1 | 4 |
| (1) | Med_Var0000791 | Exo | C17 H30 N2 O2 P2 | NA | 5.277 | 179.09638 | [M+2H]+2 | 4 |
| (1) | Med_Var0000792 | Exo | C14 H18 | NA | 6.048 | 219.17464 | [M+H+MeOH]+1 | 4 |
| (1) | Med_Var0000794 | Exo | C17 H25 N O2 | NA | 8.556 | 276.19614 | [M+H]+1 | 4 |
| (1) | Med_Var0000803 | Exo | C15 H25 N O4 | NA | 4.965 | 284.18595 | [M+H]+1 | 4 |
| (1) | Med_Var0000805 | Exo | C7 H10 | NA | 7.24 | 189.16409 | [2M+H]+1 | 4 |
| (1) | Med_Var0000806 | Exo | C20 H33 Cl P2 | NA | 6.961 | 186.09471 | [M+2H]+2 | 4 |
| (1) | Med_Var0000807 | Exo | C17 H27 N O3 | Pyreudione B_iso4 | 6.103 | 294.20668 | [M+H]+1 | 4 |
| (1) | Med_Var0000808 | Exo | C11 H14 O | Valerophenone,1-phenylpentan-1-one_iso3 | 7.241 | 145.10134 | [M+H-H2O]+1 | 4 |
| (1) | Med_Var0000812 | Exo | C17 H29 N3 O6 | Propioxatin A_iso1 | 5.33 | 372.21331 | [M+H]+1 | 4 |
| (1) | Med_Var0000813 | Exo | C17 H27 N O3 | Pyreudione B_iso5 | 4.2 | 294.20671 | [M+H]+1 | 3 |
| (1) | Med_Var0000816 | Exo | C12 H18 O | (6RS,10RS)-6,10-dimethylbicyclo[4.4.0]dec-1-en-3-one_iso1 | 6.862 | 179.14333 | [M+H]+1 | 4 |
| (1) | Med_Var0000817 | Exo | C11 H14 | NA | 6.863 | 179.14328 | [M+H+MeOH]+1 | 4 |
| (1) | Med_Var0000818 | Exo | C7 H10 O | 2,3,5-Trimethylfuran_iso2 | 6.672 | 221.15387 | [2M+H]+1 | 4 |
| (1) | Med_Var0000819 | Exo | C27 H27 N O3 | Shearilicine_iso7 | 6.006 | 207.60717 | [M+2H]+2 | 4 |
| (1) | Med_Var0000821 | Exo | C15 H24 O5 | Zosteropenilline E_iso1 | 5.091 | 285.16995 | [M+H]+1 | 3 |
| (1) | Med_Var0000823 | Exo | C17 H31 N O4 | NA | 6.085 | 314.23288 | [M+H]+1 | 4 |
| (1) | Med_Var0000825 | Exo | C15 H24 O4 | Isotrichodiol_iso12 | 6.012 | 291.15697 | [M+Na]+1 | 3 |
| (1) | Med_Var0000827 | Exo | C14 H18 O | Penidienone_iso4 | 6.121 | 203.14338 | [M+H]+1 | 4 |
| (1) | Med_Var0000830 | Exo | C17 H31 N O4 | NA | 4.808 | 314.23289 | [M+H]+1 | 4 |
| (1) | Med_Var0000831 | Exo | C13 H30 N2 P2 S | NA | 5.133 | 309.16761 | [M+H]+1 | 4 |
| (1) | Med_Var0000832 | Exo | C17 H29 N O4 | Penicillenol D | 6.191 | 312.21725 | [M+H]+1 | 4 |
| (1) | Med_Var0000833 | Exo | C13 H20 N6 O2 | NA | 6.148 | 293.17265 | [M+H]+1 | 4 |
| (1) | Med_Var0000835 | Exo | C14 H18 O | Penidienone_iso5 | 5.758 | 203.14336 | [M+H]+1 | 4 |
| (1) | Med_Var0000840 | Exo | C19 H27 N3 O5 | NA | 5.834 | 400.18476 | [M+Na]+1 | 4 |
| (1) | Med_Var0000841 | Exo | C19 H27 N4 O P | NA | 7.769 | 180.10421 | [M+2H]+2 | 4 |
| (1) | Med_Var0000847 | Exo | C15 H24 O3 | Isotrichodiol_iso6 | 6.361 | 253.18012 | [M+H]+1 | 4 |
| (1) | Med_Var0000853 | Exo | C25 H26 O3 | Spinochalcone C_iso1 | 5.277 | 188.10169 | [M+2H]+2 | 4 |
| (1) | Med_Var0000855 | Exo | C27 H25 N O3 | NA | 8.844 | 206.59931 | [M+2H]+2 | 4 |
| (1) | Med_Var0000856 | Exo | C14 H20 O | Fusidione_iso4 | 7.631 | 205.15898 | [M+H]+1 | 4 |
| (1) | Med_Var0000859 | Exo | C17 H25 N O4 | Cordypyridone D | 6.638 | 308.18597 | [M+H]+1 | 4 |
| (1) | Med_Var0000860 | Exo | NA | NA | 7.243 | 284.67819 | [M+2H]+2 | 5 |
| (1) | Med_Var0000861 | Exo | C16 H24 O4 | Stereumin R | 8.752 | 281.17504 | [M+H]+1 | 4 |
| (1) | Med_Var0000863 | Exo | C15 H20 O | Anaephene C_iso9 | 6.993 | 217.15896 | [M+H]+1 | 4 |
| (1) | Med_Var0000865 | Exo | C15 H18 O | 1-Hydroxymethyl-4-methyl-7-isopropenylaz_iso6 | 4.992 | 215.1433 | [M+H]+1 | 4 |
| (1) | Med_Var0000866 | Exo | C8 H10 | NA | 7.243 | 107.08589 | [M+H]+1 | 4 |
| (1) | Med_Var0000868 | Exo | C27 H29 N O4 | Campyridone B | 7.245 | 216.61245 | [M+2H]+2 | 4 |
| (1) | Med_Var0000869 | Exo | C8 H8 | Styrene_iso2 | 7.242 | 105.07026 | [M+H]+1 | 4 |
| (1) | Med_Var0000870 | Exo | C12 H23 N O4 | 3-hydroxy-N-(1-hydroxy-3-methylpentan-2-yl)-5-oxohexanamide_iso6 | 4.247 | 246.17028 | [M+H]+1 | 4 |
| (1) | Med_Var0000871 | Exo | C14 H18 O2 | Ochracene F_iso2 | 6.952 | 219.13818 | [M+H]+1 | 4 |
| (1) | Med_Var0000876 | Exo | C21 H31 N3 O4 | NA | 7.101 | 390.23914 | [M+H]+1 | 4 |
| (1) | Med_Var0000879 | Exo | C16 H19 N2 O5 P | NA | 5.887 | 351.11182 | [M+H]+1 | 4 |
| (1) | Med_Var0000881 | Exo | C14 H23 N9 O3 | NA | 4.621 | 366.20052 | [M+H]+1 | 4 |
| (1) | Med_Var0000882 | Exo | C15 H18 O | 1-Hydroxymethyl-4-methyl-7-isopropenylaz_iso7 | 7.171 | 215.14336 | [M+H]+1 | 4 |
| (1) | Med_Var0000885 | Exo | C15 H26 O2 | 2b-hydroxytrichoacorenol | 7.754 | 239.20084 | [M+H]+1 | 4 |
| (1) | Med_Var0000886 | Exo | C6 H11 N O2 P2 | NA | 8.374 | 233.06004 | [M+ACN+H]+1 | 4 |
| (1) | Med_Var0000890 | Exo | C14 H18 O2 | Ochracene F_iso3 | 7.202 | 219.13822 | [M+H]+1 | 4 |
| (1) | Med_Var0000893 | Exo | C16 H34 N2 O6 S2 | NA | 9.733 | 208.09917 | [M+2H]+2 | 4 |
| (1) | Med_Var0000895 | Exo | C15 H16 O | 7-Isopropenyl-4-methyl-6,7-dihydro-1-azu | 6.211 | 213.1277 | [M+H]+1 | 4 |
| (1) | Med_Var0000898 | Exo | C17 H26 O4 | Cytosporone T_iso1 | 9.941 | 295.1907 | [M+H]+1 | 4 |
| (1) | Med_Var0000900 | Exo | C11 H21 N4 O2 P | NA | 7.143 | 273.14641 | [M+H]+1 | 4 |
| (1) | Med_Var0000902 | Exo | C15 H20 O | Anaephene C_iso10 | 7.12 | 217.15896 | [M+H]+1 | 4 |
| (1) | Med_Var0000904 | Exo | C15 H18 | Guaiazulene_iso3 | 6.697 | 199.14846 | [M+H]+1 | 4 |
| (1) | Med_Var0000906 | Exo | C20 H37 Cl O P2 | NA | 6.501 | 196.10785 | [M+2H]+2 | 4 |
| (1) | Med_Var0000909 | Exo | C35 H81 N7 O15 P2 | NA | 4.91 | 451.76945 | [M+2H]+2 | 4 |
| (1) | Med_Var0000910 | Exo | C12 H14 | NA | 8.367 | 159.11703 | [M+H]+1 | 4 |
| (1) | Med_Var0000911 | Exo | C25 H22 O3 | Maculalactone L_iso3 | 9.646 | 186.08598 | [M+2H]+2 | 4 |
| (1) | Med_Var0000917 | Exo | C25 H24 O4 | Paratocarpin A_iso5 | 7.172 | 195.09137 | [M+2H]+2 | 4 |
| (1) | Med_Var0000919 | Exo | C5 H16 N4 P2 | NA | 8.221 | 195.09139 | [M+H]+1 | 4 |
| (1) | Med_Var0000923 | Exo | C15 H28 N4 O3 P2 | NA | 4.114 | 397.15209 | [M+Na]+1 | 4 |
| (1) | Med_Var0000925 | Exo | C11 H26 N5 O P | NA | 7.461 | 276.19615 | [M+H]+1 | 4 |
| (1) | Med_Var0000931 | Exo | C25 H24 O4 | Spinoflavanone A | 6.861 | 195.0913 | [M+2H]+2 | 4 |
| (1) | Med_Var0000932 | Exo | C12 H18 O | (6RS,10RS)-6,10-dimethylbicyclo[4.4.0]dec-1-en-3-one_iso2 | 6.679 | 161.13272 | [M+H-H2O]+1 | 4 |
| (1) | Med_Var0000933 | Exo | C15 H22 O5 | Clitocybulol E | 5.176 | 283.15431 | [M+H]+1 | 3 |
| (1) | Med_Var0000936 | Exo | C25 H24 O4 | Paratocarpin A_iso6 | 8.225 | 195.09139 | [M+2H]+2 | 4 |
| (1) | Med_Var0000937 | Exo | C9 H10 | NA | 5.277 | 151.11197 | [M+H+MeOH]+1 | 4 |
| (1) | Med_Var0000939 | Exo | C18 H30 N5 O3 P S | NA | 7.343 | 214.5967 | [M+2H]+2 | 4 |
| (1) | Med_Var0000940 | Exo | C15 H18 O3 | 9a-Hydroxy-3,8a-dimethyl-5-methylene4,4a,5,6,9,9a-hexahydronaphtho[2,3-b]furan-2(8aH)-one_iso3 | 4.773 | 247.13321 | [M+H]+1 | 3 |
| (1) | Med_Var0000941 | Exo | C18 H36 Cl N O6 | NA | 7.465 | 199.6184 | [M+2H]+2 | 4 |
| (1) | Med_Var0000942 | Exo | C20 H35 N3 O S2 | NA | 7.244 | 199.61839 | [M+2H]+2 | 4 |
| (1) | Med_Var0000946 | Exo | C15 H18 O2 | Meruliolactone_iso2 | 5.314 | 231.13827 | [M+H]+1 | 4 |
| (1) | Med_Var0000947 | Exo | C25 H26 O4 | Lespeol_iso3 | 7.466 | 196.09923 | [M+2H]+2 | 4 |
| (1) | Med_Var0000948 | Exo | C10 H12 | NA | 6.355 | 133.10143 | [M+H]+1 | 4 |
| (1) | Med_Var0000949 | Exo | C21 H31 N3 O4 | NA | 7.221 | 390.2392 | [M+H]+1 | 4 |
| (1) | Med_Var0000952 | Exo | C15 H20 O | Anaephene C_iso11 | 6.571 | 217.15895 | [M+H]+1 | 4 |
| (1) | Med_Var0000953 | Exo | C12 H16 O2 | 6-[(E)-Hept-1-enyl]-alpha-pyrone | 5.954 | 193.12265 | [M+H]+1 | 4 |
| (1) | Med_Var0000955 | Exo | C17 H29 N3 O6 | Propioxatin A_iso2 | 4.62 | 372.21338 | [M+H]+1 | 4 |
| (1) | Med_Var0000956 | Exo | C8 H12 O | NA | 6.496 | 125.09632 | [M+H]+1 | 4 |
| (1) | Med_Var0000957 | Exo | C17 H31 N O3 | NA | 8.15 | 298.23795 | [M+H]+1 | 4 |
| (1) | Med_Var0000959 | Exo | C15 H16 O2 | Lactarioline A | 5.47 | 229.12261 | [M+H]+1 | 4 |
| (1) | Med_Var0000961 | Exo | C15 H24 O2 | 12,13-Epoxy-9,10-trichoene-2-ol_iso11 | 3.448 | 237.18537 | [M+H]+1 | 4 |
| (1) | Med_Var0000962 | Exo | C15 H24 O3 | Isotrichodiol_iso7 | 6.815 | 253.18013 | [M+H]+1 | 4 |
| (1) | Med_Var0000963 | Exo | C15 H24 O3 | Isotrichodiol_iso8 | 8.334 | 253.18014 | [M+H]+1 | 4 |
| (1) | Med_Var0000964 | Exo | C13 H18 N6 O3 | Lupinate_iso3 | 7.247 | 307.15193 | [M+H]+1 | 4 |
| (1) | Med_Var0000965 | Exo | C15 H20 O2 | Sch 725681_iso9 | 4.911 | 233.15393 | [M+H]+1 | 4 |
| (1) | Med_Var0000966 | Exo | C15 H20 O2 | Sch 725681_iso10 | 4.804 | 233.1539 | [M+H]+1 | 4 |
| (1) | Med_Var0000967 | Exo | C17 H35 N O4 | NA | 6.181 | 318.26422 | [M+H]+1 | 4 |
| (1) | Med_Var0000968 | Exo | C15 H20 O | Anaephene C_iso12 | 7.397 | 217.15897 | [M+H]+1 | 4 |
| (1) | Med_Var0000971 | Exo | C15 H20 N4 O3 S | NA | 6.727 | 378.15922 | [M+ACN+H]+1 | 4 |
| (1) | Med_Var0000972 | Exo | C17 H23 N5 O3 S | NA | 6.698 | 378.15919 | [M+H]+1 | 4 |
| (1) | Med_Var0000975 | Exo | C17 H25 N O3 | (+/-)-5-[(tert-Butylamino)-2'-hydroxypropoxy]-3_4-dihydro-1(2H)-naphthalenone | 6.337 | 292.19108 | [M+H]+1 | 4 |
| (1) | Med_Var0000978 | Exo | C14 H20 O | Fusidione_iso5 | 6.144 | 205.15902 | [M+H]+1 | 4 |
| (1) | Med_Var0000979 | Exo | C10 H14 | NA | 6.862 | 135.11707 | [M+H]+1 | 4 |
| (1) | Med_Var0000980 | Exo | C17 H33 N O4 | Decanoylcarnitine | 6.689 | 316.24859 | [M+H]+1 | 4 |
| (1) | Med_Var0000987 | Exo | C13 H24 N6 O3 | Ficellomycin_iso3 | 3.814 | 313.19879 | [M+H]+1 | 4 |
| (1) | Med_Var0000988 | Exo | C15 H20 O | Anaephene C_iso13 | 5.566 | 217.15895 | [M+H]+1 | 4 |
| (1) | Med_Var0000989 | Exo | C19 H25 N4 O2 P | NA | 6.697 | 187.09381 | [M+2H]+2 | 4 |
| (1) | Med_Var0000990 | Exo | C13 H24 N6 O3 | Ficellomycin_iso4 | 4.665 | 313.19882 | [M+H]+1 | 4 |
| (1) | Med_Var0000991 | Exo | C14 H19 N4 O2 P | NA | 6.087 | 307.13099 | [M+H]+1 | 4 |
| (1) | Med_Var0000994 | Exo | C20 H35 N3 O2 S2 | NA | 6.862 | 207.61586 | [M+2H]+2 | 4 |
| (1) | Med_Var0000998 | Exo | C11 H16 O3 | Tubingenoic anhydride A | 8.498 | 197.11754 | [M+H]+1 | 4 |
| (1) | Med_Var0000999 | Exo | C15 H18 N4 O4 S | NA | 7.967 | 392.13848 | [M+ACN+H]+1 | 4 |
| (1) | Med_Var0001003 | Exo | C7 H8 | NA | 8.36 | 93.07035 | [M+H]+1 | 4 |
| (1) | Med_Var0001004 | Exo | C15 H20 O | Anaephene C_iso14 | 5.356 | 217.15902 | [M+H]+1 | 4 |
| (1) | Med_Var0001006 | Exo | C14 H18 O | Penidienone_iso6 | 8.812 | 203.14338 | [M+H]+1 | 4 |
| (1) | Med_Var0001007 | Exo | C14 H23 N4 O2 P | NA | 5.998 | 311.16215 | [M+H]+1 | 4 |
| (1) | Med_Var0001008 | Exo | C8 H12 O | NA | 7.469 | 107.08589 | [M+H-H2O]+1 | 4 |
| (1) | Med_Var0001012 | Exo | C13 H14 N6 O | NA | 7.263 | 271.13075 | [M+H]+1 | 4 |
| (1) | Med_Var0001015 | Exo | C16 H29 N3 O5 | NA | 4.624 | 344.21829 | [M+H]+1 | 4 |
| (1) | Med_Var0001016 | Exo | C14 H20 | NA | 7.932 | 189.16408 | [M+H]+1 | 4 |
| (1) | Med_Var0001019 | Exo | C18 H30 N2 O2 S2 | NA | 5.888 | 186.09473 | [M+2H]+2 | 4 |
| (1) | Med_Var0001021 | Exo | C15 H18 O2 | 3-methyl-5-(3-methylbut-2-en-1-yl)-1H-isochromen-6-ol | 8.839 | 231.13826 | [M+H]+1 | 4 |
| (1) | Med_Var0001023 | Exo | C17 H23 N O2 | 2-n-octyl-4-hydroxyquinoline N-oxide | 8.296 | 274.18048 | [M+H]+1 | 4 |
| (1) | Med_Var0001024 | Exo | C15 H22 O5 | Piltunine A_iso3 | 6.656 | 283.15432 | [M+H]+1 | 3 |
| (1) | Med_Var0001025 | Exo | C15 H21 N3 O5 | Streptcytosine G | 4.432 | 346.13775 | [M+Na]+1 | 4 |
| (1) | Med_Var0001027 | Exo | C15 H33 O5 P S | NA | 6.18 | 179.09636 | [M+2H]+2 | 4 |
| (1) | Med_Var0001029 | Exo | C16 H29 N3 O5 | NA | 4.727 | 344.21842 | [M+H]+1 | 4 |
| (1) | Med_Var0001031 | Exo | NA | NA | 6.181 | 179.09638 | [M+H-H2O]+1 | 5 |
| (1) | Med_Var0001032 | Exo | C25 H22 O3 | Maculalactone L_iso4 | 6.764 | 186.08596 | [M+2H]+2 | 4 |
| (1) | Med_Var0001033 | Exo | C15 H23 N3 O3 S | NA | 6.96 | 163.58084 | [M+2H]+2 | 4 |
| (1) | Med_Var0001035 | Exo | C23 H20 N2 O2 | Epurpurin B_iso2 | 5.458 | 357.15879 | [M+H]+1 | 4 |
| (1) | Med_Var0001038 | Exo | C25 H24 O5 | Glyinflanin G_iso3 | 5.628 | 203.08874 | [M+2H]+2 | 4 |
| (1) | Med_Var0001039 | Exo | C15 H24 O2 | 12,13-Epoxy-9,10-trichoene-2-ol_iso12 | 3.453 | 237.18518 | [M+H]+1 | 4 |
| (1) | Med_Var0001040 | Exo | C7 H10 O | 2,3,5-Trimethylfuran_iso3 | 7.475 | 111.08079 | [M+H]+1 | 4 |
| (1) | Med_Var0001042 | Exo | C15 H24 O4 | Trichotriol_iso10 | 5.788 | 291.15693 | [M+Na]+1 | 4 |
| (1) | Med_Var0001044 | Exo | C15 H18 O | 1-Hydroxymethyl-4-methyl-7-isopropenylaz_iso8 | 9.064 | 215.14332 | [M+H]+1 | 4 |
| (1) | Med_Var0001046 | Exo | C17 H24 O5 | 15-Decalonectrin_iso5 | 9.494 | 331.15188 | [M+Na]+1 | 4 |
| (1) | Med_Var0001048 | Exo | C15 H24 O4 | Isotrichodiol_iso13 | 6.104 | 269.17505 | [M+H]+1 | 3 |
| (1) | Med_Var0001049 | Exo | C13 H14 | NA | 6.502 | 203.14334 | [M+H+MeOH]+1 | 4 |
| (1) | Med_Var0001053 | Exo | C18 H40 N O2 P S2 | NA | 8.34 | 199.61841 | [M+2H]+2 | 4 |
| (1) | Med_Var0001054 | Exo | C14 H18 N6 O3 | NA | 6.649 | 319.15197 | [M+H]+1 | 4 |
| (1) | Med_Var0001055 | Exo | C15 H24 O5 | Zosteropenilline E_iso2 | 4.798 | 307.15189 | [M+Na]+1 | 4 |
| (1) | Med_Var0001057 | Exo | C12 H12 | NA | 7.244 | 157.10145 | [M+H]+1 | 4 |
| (1) | Med_Var0001060 | Exo | C19 H25 N4 O P | NA | 5.36 | 179.09638 | [M+2H]+2 | 4 |
| (1) | Med_Var0001061 | Exo | NA | NA | 5.361 | 179.09641 | [M+H-H2O]+1 | 5 |
| (1) | Med_Var0001063 | Exo | C14 H22 O | NA | 9.981 | 189.16411 | [M+H-H2O]+1 | 4 |
| (1) | Med_Var0001064 | Exo | C30 H36 O6 | 2',4',6',3-Tetrahydroxy-3'-geranyl-6",6"-dimethylpyrano [ 2",3":4,5 ] dihydrochalcone | 5.746 | 247.13318 | [M+2H]+2 | 4 |
| (1) | Med_Var0001069 | Exo | C7 H10 O2 | 5-Hydroxy-2,3-dimethylcyclopent-2-enone_iso2 | 6.085 | 127.07563 | [M+H]+1 | 4 |
| (1) | Med_Var0001071 | Exo | C13 H25 N O9 | NA | 5.458 | 179.09634 | [M+H+NH4]+2 | 4 |
| (1) | Med_Var0001074 | Exo | NA | NA | 5.458 | 179.09638 | [M+H-H2O]+1 | 5 |
| (1) | Med_Var0001077 | Exo | C15 H31 N3 O9 | Sorbistin A1_iso4 | 5.276 | 199.60969 | [M+2H]+2 | 4 |
| (1) | Med_Var0001078 | Exo | C15 H20 O | Anaephene C_iso15 | 5.276 | 217.15898 | [M+H]+1 | 4 |
| (1) | Med_Var0001080 | Exo | C25 H26 O3 | Spinochalcone C_iso2 | 5.459 | 188.10167 | [M+2H]+2 | 4 |
| (1) | Med_Var0001081 | Exo | NA | NA | 5.459 | 188.10176 | [M+H]+1 | 5 |
| (1) | Med_Var0001083 | Exo | C6 H8 O | Cyclohex-2-enone | 9.496 | 97.06523 | [M+H]+1 | 4 |
| (1) | Med_Var0001085 | Exo | C15 H18 O | 1-Hydroxymethyl-4-methyl-7-isopropenylaz_iso9 | 4.912 | 215.14334 | [M+H]+1 | 4 |
| (1) | Med_Var0001088 | Exo | C14 H25 N3 O4 | NA | 4.298 | 300.19207 | [M+H]+1 | 4 |
| (1) | Med_Var0001089 | Exo | C19 H27 N3 O5 | NA | 5.332 | 378.20282 | [M+H]+1 | 4 |
| (1) | Med_Var0001093 | Exo | C18 H25 N3 O4 | NA | 5.39 | 370.17419 | [M+Na]+1 | 4 |
| (1) | Med_Var0001094 | Exo | C15 H24 O5 | 13-hydroxypereniporin A | 6.233 | 307.15181 | [M+Na]+1 | 3 |
| (1) | Med_Var0001095 | Exo | C16 H12 N6 O | NA | 4.96 | 305.11524 | [M+H]+1 | 4 |
| (1) | Med_Var0001098 | Exo | C14 H18 O | Penidienone_iso7 | 5.552 | 203.14339 | [M+H]+1 | 4 |
| (1) | Med_Var0001102 | Exo | C15 H22 O5 | Piltunine A_iso4 | 7.648 | 283.15427 | [M+H]+1 | 3 |
| (1) | Med_Var0001105 | Exo | C11 H14 | NA | 7.466 | 179.14332 | [M+H+MeOH]+1 | 4 |
| (1) | Med_Var0001109 | Exo | C18 H34 N2 P2 S | NA | 8.372 | 187.10256 | [M+2H]+2 | 4 |
| (1) | Med_Var0001112 | Exo | NA | NA | 5.363 | 188.10176 | [M+H]+1 | 5 |
| (1) | Med_Var0001114 | Exo | NA | NA | 5.36 | 197.10702 | [M+2H]+2 | 5 |
| (1) | Med_Var0001115 | Exo | C25 H26 O3 | Spinochalcone C_iso3 | 5.361 | 188.10169 | [M+2H]+2 | 4 |
| (1) | Med_Var0001116 | Exo | C11 H14 O | Valerophenone,1-phenylpentan-1-one_iso4 | 6.598 | 163.11198 | [M+H]+1 | 4 |
| (1) | Med_Var0001118 | Exo | C17 H24 N2 O5 | Daryamide E | 7.171 | 169.09194 | [M+2H]+2 | 4 |
| (1) | Med_Var0001119 | Exo | C15 H16 O | Zafrin_iso2 | 8.84 | 213.12771 | [M+H]+1 | 4 |
| (1) | Med_Var0001120 | Exo | C15 H24 O3 | Isotrichodiol_iso9 | 5.274 | 253.18014 | [M+H]+1 | 4 |
| (1) | Med_Var0001122 | Exo | C10 H14 | NA | 6.361 | 135.11707 | [M+H]+1 | 4 |
| (1) | Med_Var0001124 | Exo | C11 H10 | NA | 8.811 | 143.08574 | [M+H]+1 | 4 |
| (1) | Med_Var0001125 | Exo | C22 H34 Cl N O2 S | NA | 7.523 | 206.60793 | [M+2H]+2 | 4 |
| (1) | Med_Var0001126 | Exo | C10 H14 O | (-)-Isopiperitenone_iso2 | 5.359 | 151.11196 | [M+H]+1 | 4 |
| (1) | Med_Var0001127 | Exo | C14 H20 O2 | 2_6-Di-tert-butylbenzoquinone | 5.545 | 221.15393 | [M+H]+1 | 4 |
| (1) | Med_Var0001129 | Exo | C15 H20 O4 | Trichodermaloid C | 4.876 | 247.13325 | [M+H-H2O]+1 | 3 |
| (1) | Med_Var0001137 | Exo | C15 H18 O | 1-Hydroxymethyl-4-methyl-7-isopropenylaz_iso10 | 4.807 | 215.14329 | [M+H]+1 | 4 |
| (1) | Med_Var0001138 | Exo | C18 H31 Cl N O P S | NA | 7.52 | 376.16106 | [M+H]+1 | 4 |
| (1) | Med_Var0001140 | Exo | NA | NA | 6.501 | 191.14333 | [M+H+MeOH]+1 | 5 |
| (1) | Med_Var0001141 | Exo | C13 H20 N6 O | NA | 8.311 | 277.1778 | [M+H]+1 | 4 |
| (1) | Med_Var0001143 | Exo | C15 H18 O | 1-Hydroxymethyl-4-methyl-7-isopropenylaz_iso11 | 10.349 | 215.14331 | [M+H]+1 | 4 |
| (1) | Med_Var0001145 | Exo | C17 H25 N O2 | NA | 7.86 | 276.19613 | [M+H]+1 | 4 |
| (1) | Med_Var0001146 | Exo | NA | NA | 8.239 | 157.58389 | [M+H]+1 | 5 |
| (1) | Med_Var0001147 | Exo | C21 H27 N5 O4 | NA | 4.936 | 414.21412 | [M+H]+1 | 4 |
| (1) | Med_Var0001149 | Exo | C25 H24 O4 | Paratocarpin A_iso9 | 6.671 | 195.09127 | [M+2H]+2 | 4 |
| (1) | Med_Var0001150 | Exo | C15 H25 N O3 P2 | NA | 7.169 | 165.57265 | [M+2H]+2 | 4 |
| (1) | Med_Var0001152 | Exo | C15 H26 N3 O4 P S | NA | 7.899 | 376.14359 | [M+H]+1 | 4 |
| (1) | Med_Var0001155 | Exo | C15 H20 N4 O4 S | NA | 7.171 | 353.12749 | [M+H]+1 | 4 |
| (1) | Med_Var0001158 | Exo | C16 H27 N5 | NA | 5.277 | 290.23294 | [M+H]+1 | 4 |
| (1) | Med_Var0001164 | Exo | C20 H20 O9 | Peniciphenalenin B | 7.465 | 405.12001 | [M+H]+1 | 4 |
| (1) | Myc_Var0000043 | Endo | C15 H24 O2 | 12,13-Epoxy-9,10-trichoene-2-ol_iso1 | 8.915 | 237.18491 | [M+H]+1 | 3 |
| (1) | Myc_Var0000055 | Endo | C15 H26 O5 | Cytospolide Q_iso1 | 6.506 | 309.16713 | [M+Na]+1 | 3 |
| (1) | Myc_Var0000056 | Endo | C12 H24 N2 O7 | Fructoselysine | 6.506 | 309.16715 | [M+H]+1 | 4 |
| (1) | Myc_Var0000093 | Endo | C15 H24 O | 2-Hydroxytrichodiene_iso3 | 10.845 | 203.1795 | [M+H-H2O]+1 | 4 |
| (1) | Myc_Var0000096 | Endo | C15 H24 O4 | Trichotriol_iso1 | 6.506 | 269.1748 | [M+H]+1 | 3 |
| (1) | Myc_Var0000112 | Endo | C17 H26 O5 | Guignardone I | 6.073 | 333.16714 | [M+Na]+1 | 3 |
| (1) | Myc_Var0000127 | Endo | C30 H44 O | NA | 14.68 | 421.34648 | [M+H]+1 | 4 |
| (1) | Myc_Var0000133 | Endo | C19 H28 O6 | Geumsanol C_iso1 | 8.455 | 375.1777 | [M+Na]+1 | 4 |
| (1) | Myc_Var0000143 | Endo | C14 H27 P3 | NA | 6.831 | 289.14104 | [M+H]+1 | 4 |
| (1) | Myc_Var0000149 | Endo | C19 H28 O6 | Geumsanol C_iso2 | 7.309 | 375.17774 | [M+Na]+1 | 4 |
| (1) | Myc_Var0000154 | Endo | C17 H26 O5 | Arundinol C | 7.645 | 333.16711 | [M+Na]+1 | 3 |
| (1) | Myc_Var0000158 | Endo | C15 H22 O4 | Curvularol_iso2 | 4.887 | 289.14102 | [M+Na]+1 | 3 |
| (1) | Myc_Var0000166 | Endo | C15 H24 O3 | Isotrichodiol_iso2 | 7.253 | 253.17988 | [M+H]+1 | 3 |
| (1) | Myc_Var0000174 | Endo | C15 H22 O3 | Trichodermol_iso3 | 6.506 | 251.16421 | [M+H]+1 | 3 |
| (1) | Myc_Var0000178 | Endo | C15 H24 O4 | Isotrichodiol_iso6 | 4.492 | 291.1566 | [M+Na]+1 | 3 |
| (1) | Myc_Var0000179 | Endo | C14 H29 P3 | NA | 4.487 | 291.15679 | [M+H]+1 | 4 |
| (1) | Myc_Var0000181 | Endo | C9 H10 | NA | 9.144 | 119.08572 | [M+H]+1 | 4 |
| (1) | Myc_Var0000184 | Endo | C15 H22 O | Ent-?-Cyperone | 8.914 | 219.17439 | [M+H]+1 | 3 |
| (1) | Myc_Var0000195 | Endo | C13 H16 N6 O2 | NA | 7.215 | 289.14114 | [M+H]+1 | 4 |
| (1) | Myc_Var0000198 | Endo | C14 H18 O2 | Ochracene F | 6.221 | 219.138 | [M+H]+1 | 4 |
| (1) | Myc_Var0000209 | Endo | C19 H28 O6 | Geumsanol C_iso3 | 9.296 | 375.17778 | [M+Na]+1 | 4 |
| (1) | Myc_Var0000212 | Endo | C30 H54 O4 | NA | 13.049 | 501.39132 | [M+Na]+1 | 4 |
| (1) | Myc_Var0000214 | Endo | C15 H22 O4 | Curvularol_iso3 | 7.533 | 267.15913 | [M+H]+1 | 3 |
| (1) | Myc_Var0000225 | Endo | C17 H29 N O3 | Cyclizidine G | 8.393 | 296.22206 | [M+H]+1 | 4 |
| (1) | Myc_Var0000231 | Endo | C15 H20 O3 | Arecolactone_iso1 | 6.22 | 249.14859 | [M+H]+1 | 3 |
| (1) | Myc_Var0000234 | Endo | C11 H14 | NA | 9.144 | 147.11692 | [M+H]+1 | 4 |
| (1) | Myc_Var0000237 | Endo | C15 H18 O | 1-Hydroxymethyl-4-methyl-7-isopropenylaz_iso1 | 8.395 | 215.14315 | [M+H]+1 | 4 |
| (1) | Myc_Var0000238 | Endo | C15 H24 O4 | Isotrichodiol_iso7 | 4.92 | 291.15655 | [M+Na]+1 | 3 |
| (1) | Myc_Var0000239 | Endo | C14 H29 P3 | NA | 4.92 | 291.15663 | [M+H]+1 | 4 |
| (1) | Myc_Var0000248 | Endo | C15 H20 O3 | 2-oxoeremophila-1(10),11(13)-dien-12-oic acid | 7.214 | 249.14865 | [M+H]+1 | 3 |
| (1) | Myc_Var0000254 | Endo | C11 H21 N4 O3 P | NA | 7.299 | 289.14115 | [M+H]+1 | 4 |
| (1) | Myc_Var0000265 | Endo | C10 H12 | NA | 9.144 | 133.10131 | [M+H]+1 | 4 |
| (1) | Myc_Var0000266 | Endo | C14 H27 P3 | NA | 5.784 | 289.14108 | [M+H]+1 | 4 |
| (1) | Myc_Var0000267 | Endo | C13 H18 N6 O3 | Lupinate_iso1 | 5.785 | 289.14121 | [M+H-H2O]+1 | 4 |
| (1) | Myc_Var0000297 | Endo | NA | NA | 6.183 | 295.18798 | [M+H]+1 | 5 |
| (1) | Myc_Var0000300 | Endo | C15 H20 N6 O3 | NA | 7.145 | 333.16745 | [M+H]+1 | 4 |
| (1) | Myc_Var0000307 | Endo | C8 H8 | Styrene_iso1 | 9.144 | 105.07018 | [M+H]+1 | 4 |
| (1) | Myc_Var0000308 | Endo | C15 H20 O3 | Arecolactone_iso2 | 7.534 | 249.14861 | [M+H]+1 | 3 |
| (1) | Myc_Var0000310 | Endo | C15 H24 O3 | Isotrichodiol_iso3 | 7.477 | 253.17989 | [M+H]+1 | 3 |
| (1) | Myc_Var0000314 | Endo | C13 H22 N6 O | NA | 7.754 | 279.19311 | [M+H]+1 | 4 |
| (1) | Myc_Var0000319 | Endo | C21 H35 N O5 | NA | 10.594 | 382.25894 | [M+H]+1 | 4 |
| (1) | Myc_Var0000322 | Endo | C21 H35 N O6 | NA | 7.309 | 398.25381 | [M+H]+1 | 4 |
| (1) | Myc_Var0000326 | Endo | C15 H24 O4 | Trichotriol_iso3 | 6.872 | 291.15671 | [M+Na]+1 | 3 |
| (1) | Myc_Var0000327 | Endo | C15 H20 O2 | Sch 725681_iso1 | 8.85 | 233.15371 | [M+H]+1 | 4 |
| (1) | Myc_Var0000329 | Endo | C15 H18 O3 | Nigriterpene B | 8.278 | 247.13305 | [M+H]+1 | 4 |
| (1) | Myc_Var0000330 | Endo | C15 H16 | NA | 10.554 | 197.13267 | [M+H]+1 | 4 |
| (1) | Myc_Var0000336 | Endo | C15 H24 O4 | Trichotriol_iso4 | 5.527 | 269.17481 | [M+H]+1 | 3 |
| (1) | Myc_Var0000338 | Endo | C15 H24 O | 2-Hydroxytrichodiene_iso4 | 8.339 | 203.17956 | [M+H-H2O]+1 | 3 |
| (1) | Myc_Var0000349 | Endo | C14 H33 P3 | NA | 6.358 | 295.18801 | [M+H]+1 | 4 |
| (1) | Myc_Var0000355 | Endo | C15 H20 O3 | Arecolactone_iso3 | 6.118 | 249.14862 | [M+H]+1 | 3 |
| (1) | Myc_Var0000356 | Endo | C15 H22 | Rulepidadiene B_iso2 | 12.552 | 203.17955 | [M+H]+1 | 4 |
| (1) | Myc_Var0000357 | Endo | C17 H24 N6 O2 | NA | 12.551 | 345.20363 | [M+H]+1 | 4 |
| (1) | Myc_Var0000376 | Endo | C24 H28 O10 | Daldinin C | 8.402 | 239.0903 | [M+2H]+2 | 4 |
| (1) | Myc_Var0000378 | Endo | C13 H16 N6 O | NA | 8.16 | 273.14616 | [M+H]+1 | 4 |
| (1) | Myc_Var0000380 | Endo | C25 H22 O4 | 5-Hydroxy-8,8-dimethyl-6- [ (1E) -3-methyl-1,3-butadienyl ] -2-phenyl-4H,8H-benzo [ 1,2-b:3,4-b' ] dipyran-4-one | 6.968 | 194.08341 | [M+2H]+2 | 4 |
| (1) | Myc_Var0000398 | Endo | C15 H22 O4 | Curvularol_iso4 | 5.899 | 267.15922 | [M+H]+1 | 3 |
| (1) | Myc_Var0000418 | Endo | C9 H10 O | 1-Phenylpropan-2-one | 8.914 | 135.08056 | [M+H]+1 | 3 |
| (1) | Myc_Var0000421 | Endo | C17 H26 O5 | Trichodermarin F_iso1 | 6.174 | 333.16725 | [M+Na]+1 | 3 |
| (1) | Myc_Var0000425 | Endo | C15 H20 O | Anaephene C_iso2 | 7.252 | 217.15878 | [M+H]+1 | 4 |
| (1) | Myc_Var0000435 | Endo | C15 H24 O2 | 12,13-Epoxy-9,10-trichoene-2-ol_iso3 | 5.281 | 237.18503 | [M+H]+1 | 3 |
| (1) | Myc_Var0000439 | Endo | C10 H12 O3 | 6-dimethyl-4-hydrxy-2-methoxybenzaldehyde (_iso2 | 5.751 | 181.08606 | [M+H]+1 | 4 |
| (1) | Myc_Var0000443 | Endo | C15 H24 O5 | Harzianol acid | 5.636 | 307.15165 | [M+Na]+1 | 3 |
| (1) | Myc_Var0000462 | Endo | C15 H22 O | Anaephene A_iso3 | 6.369 | 219.17442 | [M+H]+1 | 3 |
| (1) | Myc_Var0000466 | Endo | C17 H29 N O4 | YM-47515_iso1 | 5.9 | 312.21706 | [M+H]+1 | 4 |
| (1) | Myc_Var0000468 | Endo | C15 H20 O4 | Rhinomilisin H_iso3 | 7.68 | 265.14359 | [M+H]+1 | 4 |
| (1) | Myc_Var0000480 | Endo | C15 H22 | Rulepidadiene B_iso3 | 13.286 | 203.1796 | [M+H]+1 | 4 |
| (1) | Myc_Var0000483 | Endo | C17 H33 N O5 | NA | 6.505 | 332.24327 | [M+H]+1 | 4 |
| (1) | Myc_Var0000486 | Endo | C17 H26 O4 | Cytosporone T_iso1 | 9.57 | 317.1724 | [M+Na]+1 | 4 |
| (1) | Myc_Var0000492 | Endo | C15 H24 O4 | Isotrichodiol_iso8 | 6.108 | 291.15677 | [M+Na]+1 | 3 |
| (1) | Myc_Var0000499 | Endo | C15 H22 O3 | Trichodermol_iso4 | 6.774 | 273.14623 | [M+Na]+1 | 3 |
| (1) | Myc_Var0000500 | Endo | C2 H7 N O2 P2 | NA | 11.217 | 181.02856 | [M+ACN+H]+1 | 4 |
| (1) | Myc_Var0000501 | Endo | C15 H22 O3 | Trichodermol_iso5 | 7.839 | 251.16433 | [M+H]+1 | 3 |
| (1) | Myc_Var0000508 | Endo | C15 H20 O2 | Sch 725681_iso3 | 8.198 | 233.15378 | [M+H]+1 | 4 |
| (1) | Myc_Var0000510 | Endo | C15 H22 O5 | Stereumin U | 6.039 | 265.14357 | [M+H-H2O]+1 | 3 |
| (1) | Myc_Var0000515 | Endo | C15 H20 O | Anaephene C_iso3 | 10.598 | 217.1588 | [M+H]+1 | 4 |
| (1) | Myc_Var0000517 | Endo | C19 H33 N O5 | NA | 6.073 | 356.24325 | [M+H]+1 | 4 |
| (1) | Myc_Var0000521 | Endo | C25 H26 O5 | Flemiwallichin D | 6.506 | 204.09647 | [M+2H]+2 | 4 |
| (1) | Myc_Var0000523 | Endo | C19 H31 N3 O3 P2 | NA | 9.662 | 206.59921 | [M+2H]+2 | 4 |
| (1) | Myc_Var0000525 | Endo | C15 H22 O4 | 3?,6?-dihydroxycinnamolide | 6.537 | 267.15923 | [M+H]+1 | 3 |
| (1) | Myc_Var0000530 | Endo | C25 H24 O4 | Paratocarpin A | 8.394 | 195.09127 | [M+2H]+2 | 4 |
| (1) | Myc_Var0000535 | Endo | C14 H18 | NA | 8.394 | 187.14828 | [M+H]+1 | 4 |
| (1) | Myc_Var0000543 | Endo | C17 H27 N O3 | Pyreudione B_iso1 | 6.221 | 294.20646 | [M+H]+1 | 4 |
| (1) | Myc_Var0000544 | Endo | C15 H20 O2 | Sch 725681_iso4 | 7.289 | 233.15376 | [M+H]+1 | 4 |
| (1) | Myc_Var0000552 | Endo | C27 H27 N O3 | Shearilicine_iso1 | 7.251 | 207.60706 | [M+2H]+2 | 4 |
| (1) | Myc_Var0000564 | Endo | C15 H24 O | 2-Hydroxytrichodiene_iso5 | 11.537 | 221.19014 | [M+H]+1 | 3 |
| (1) | Myc_Var0000570 | Endo | C11 H27 N4 O2 P | NA | 7.515 | 279.1932 | [M+H]+1 | 4 |
| (1) | Myc_Var0000576 | Endo | C14 H27 P3 | NA | 4.972 | 289.14112 | [M+H]+1 | 4 |
| (1) | Myc_Var0000589 | Endo | C11 H21 N4 O3 P | NA | 5.077 | 289.14114 | [M+H]+1 | 4 |
| (1) | Myc_Var0000590 | Endo | C14 H16 O3 | Sorbicillin | 12.888 | 233.11737 | [M+H]+1 | 4 |
| (1) | Myc_Var0000594 | Endo | C13 H20 N6 O2 | NA | 7.538 | 293.17243 | [M+H]+1 | 4 |
| (1) | Myc_Var0000601 | Endo | C13 H22 N6 O2 | NA | 6.005 | 295.18809 | [M+H]+1 | 4 |
| (1) | Myc_Var0000605 | Endo | C17 H26 O6 | 3?-hydroxy-6-O-acetylpereniporin A | 5.175 | 349.16223 | [M+Na]+1 | 4 |
| (1) | Myc_Var0000613 | Endo | C15 H20 O3 | Arecolactone_iso4 | 7.119 | 271.13058 | [M+Na]+1 | 3 |
| (1) | Myc_Var0000617 | Endo | C10 H12 | NA | 11.217 | 133.10131 | [M+H]+1 | 4 |
| (1) | Myc_Var0000621 | Endo | C15 H26 O5 | Cytospolide Q_iso2 | 5.771 | 309.1673 | [M+Na]+1 | 3 |
| (1) | Myc_Var0000624 | Endo | C14 H16 O | NA | 12.888 | 201.12756 | [M+H]+1 | 4 |
| (1) | Myc_Var0000633 | Endo | C9 H19 N8 O3 P S | NA | 6.97 | 392.13824 | [M+ACN+H]+1 | 4 |
| (1) | Myc_Var0000634 | Endo | C17 H21 N5 O4 S | NA | 6.967 | 392.13824 | [M+H]+1 | 4 |
| (1) | Myc_Var0000635 | Endo | C15 H20 O2 | Sch 725681_iso5 | 7.182 | 233.15377 | [M+H]+1 | 4 |
| (1) | Myc_Var0000638 | Endo | C25 H26 O4 | Lespeol | 8.517 | 196.09912 | [M+2H]+2 | 4 |
| (1) | Myc_Var0000645 | Endo | C17 H26 O2 | PI-200_iso2 | 10.664 | 263.2007 | [M+H]+1 | 4 |
| (1) | Myc_Var0000646 | Endo | C25 H22 O3 | Maculalactone L_iso1 | 9.662 | 186.08592 | [M+2H]+2 | 4 |
| (1) | Myc_Var0000654 | Endo | C2 H7 N O2 P2 | NA | 10.844 | 181.02854 | [M+ACN+H]+1 | 4 |
| (1) | Myc_Var0000657 | Endo | C11 H21 N4 O2 P | NA | 7.323 | 273.1463 | [M+H]+1 | 4 |
| (1) | Myc_Var0000659 | Endo | C15 H22 O4 | 6-dehydroxy-6-oxopereniporin A_iso1 | 5.783 | 305.11509 | [M+K]+1 | 3 |
| (1) | Myc_Var0000662 | Endo | C17 H24 O3 | Lorneic acid B | 10.591 | 277.17998 | [M+H]+1 | 4 |
| (1) | Myc_Var0000663 | Endo | C15 H20 O | Anaephene C_iso4 | 10.055 | 217.1588 | [M+H]+1 | 4 |
| (1) | Myc_Var0000672 | Endo | C15 H22 O4 | 6-dehydroxy-6-oxopereniporin A_iso2 | 6.187 | 267.15926 | [M+H]+1 | 4 |
| (1) | Myc_Var0000680 | Endo | C27 H27 N O3 | Shearilicine_iso3 | 7.476 | 207.60706 | [M+2H]+2 | 4 |
| (1) | Myc_Var0000683 | Endo | C8 H8 | Styrene_iso2 | 11.217 | 105.07018 | [M+H]+1 | 4 |
| (1) | Myc_Var0000686 | Endo | C14 H24 N2 O7 | Actinospectacin | 6.642 | 333.16727 | [M+H]+1 | 2a |
| (1) | Myc_Var0000693 | Endo | C15 H22 O | Anaephene A_iso9 | 9.348 | 219.17445 | [M+H]+1 | 3 |
| (1) | Myc_Var0000701 | Endo | C15 H22 | Rulepidadiene B_iso5 | 10.664 | 203.1796 | [M+H]+1 | 4 |
| (1) | Myc_Var0000702 | Endo | C15 H24 O4 | Trichotriol_iso5 | 6.681 | 269.17487 | [M+H]+1 | 3 |
| (1) | Myc_Var0000707 | Endo | C12 H16 | NA | 11.218 | 161.13259 | [M+H]+1 | 4 |
| (1) | Myc_Var0000712 | Endo | C11 H23 N4 O3 P | NA | 4.817 | 291.15681 | [M+H]+1 | 4 |
| (1) | Myc_Var0000719 | Endo | C15 H22 O4 | Curvularol_iso5 | 7.448 | 267.15923 | [M+H]+1 | 3 |
| (1) | Myc_Var0000726 | Endo | C30 H50 O2 | 4,4-Dimethyl-14?-formyl-5?-cholesta-8-en-3?-ol | 13.049 | 443.38871 | [M+H]+1 | 3 |
| (1) | Myc_Var0000733 | Endo | C15 H25 N O4 | NA | 5.784 | 284.18579 | [M+H]+1 | 4 |
| (1) | Myc_Var0000737 | Endo | C15 H20 O3 | 1_2-Dihydrosantonin | 7.674 | 249.14867 | [M+H]+1 | 4 |
| (1) | Myc_Var0000750 | Endo | C17 H26 O4 | Cytosporone T_iso2 | 9.962 | 317.17249 | [M+Na]+1 | 4 |
| (1) | Myc_Var0000756 | Endo | C15 H16 O | Zafrin_iso2 | 8.252 | 213.12759 | [M+H]+1 | 4 |
| (1) | Myc_Var0000757 | Endo | C15 H22 O4 | Curvularol_iso6 | 5.715 | 267.15927 | [M+H]+1 | 3 |
| (1) | Myc_Var0000768 | Endo | C17 H24 O5 | 15-Decalonectrin_iso6 | 9.508 | 326.19624 | [M+NH4]+1 | 4 |
| (1) | Myc_Var0000769 | Endo | C17 H27 N O5 | Phyllostictine A | 9.507 | 326.19637 | [M+H]+1 | 4 |
| (1) | Myc_Var0000785 | Endo | C13 H16 N6 O2 | NA | 5.324 | 289.14115 | [M+H]+1 | 4 |
| (1) | Myc_Var0000787 | Endo | C25 H22 O3 | Maculalactone L_iso2 | 8.24 | 186.08595 | [M+2H]+2 | 4 |
| (1) | Myc_Var0000788 | Endo | C26 H51 N6 O3 P | NA | 11.298 | 527.38451 | [M+H]+1 | 4 |
| (1) | Myc_Var0000790 | Endo | C15 H20 O2 | Sch 725681_iso6 | 10.37 | 233.15376 | [M+H]+1 | 4 |
| (1) | Myc_Var0000794 | Endo | NA | NA | 7.549 | 411.19501 | [M+H]+1 | 5 |
| (1) | Myc_Var0000796 | Endo | C15 H20 O3 | Arecolactone_iso5 | 8.768 | 249.1487 | [M+H]+1 | 4 |
| (1) | Myc_Var0000799 | Endo | C17 H27 N O3 | Pyreudione B_iso2 | 7.118 | 294.20651 | [M+H]+1 | 4 |
| (1) | Myc_Var0000801 | Endo | C25 H45 N O7 | NA | 10.756 | 472.32711 | [M+H]+1 | 4 |
| (1) | Myc_Var0000805 | Endo | C17 H26 N2 O6 | NA | 9.662 | 178.09716 | [M+2H]+2 | 4 |
| (1) | Myc_Var0000807 | Endo | C19 H35 N3 O13 | A-396-I | 10.872 | 257.61582 | [M+2H]+2 | 4 |
| (1) | Myc_Var0000811 | Endo | C30 H45 N O10 S | 8-demethyl epothilone A 3-?-D-arabinofuranoside | 6.967 | 306.64598 | [M+2H]+2 | 4 |
| (1) | Myc_Var0000815 | Endo | C21 H34 O3 | Brassicicene I | 10.672 | 335.25821 | [M+H]+1 | 4 |
| (1) | Myc_Var0000816 | Endo | C15 H24 O | 2-Hydroxytrichodiene_iso7 | 10.115 | 221.19012 | [M+H]+1 | 4 |
| (1) | Myc_Var0000837 | Endo | C15 H16 O2 | Variabilone_iso2 | 13.182 | 229.12244 | [M+H]+1 | 4 |
| (1) | Myc_Var0000840 | Endo | C13 H22 N6 O2 | NA | 5.28 | 295.18814 | [M+H]+1 | 4 |
| (1) | Myc_Var0000847 | Endo | C15 H24 O2 | 12,13-Epoxy-9,10-trichoene-2-ol_iso5 | 5.463 | 237.18505 | [M+H]+1 | 3 |
| (1) | Myc_Var0000851 | Endo | C13 H18 N6 O2 | NA | 5.859 | 291.15676 | [M+H]+1 | 4 |
| (1) | Myc_Var0000853 | Endo | C17 H26 O5 | Trichodermarin F_iso2 | 7.455 | 333.16741 | [M+Na]+1 | 3 |
| (1) | Myc_Var0000854 | Endo | C15 H18 O | 1-Hydroxymethyl-4-methyl-7-isopropenylaz_iso4 | 9.085 | 215.14318 | [M+H]+1 | 4 |
| (1) | Myc_Var0000860 | Endo | C32 H50 O3 | Methyl 24-methylene-3-oxolanost-8-en-26-oic ester | 13.766 | 483.3813 | [M+H]+1 | 4 |
| (1) | Myc_Var0000866 | Endo | C16 H24 O4 | 4-epi-8?-hydroxy-15-epi-brefeldin C | 8.768 | 303.15681 | [M+Na]+1 | 4 |
| (1) | Myc_Var0000871 | Endo | C15 H20 O | Anaephene C_iso5 | 8.846 | 217.15882 | [M+H]+1 | 4 |
| (1) | Myc_Var0000883 | Endo | C16 H31 O P3 | NA | 6.247 | 333.16742 | [M+H]+1 | 4 |
| (1) | Myc_Var0000892 | Endo | C17 H23 N3 O4 | Blastmycetin C | 5.708 | 356.15824 | [M+Na]+1 | 4 |
| (1) | Myc_Var0000901 | Endo | C14 H20 N6 O3 | NA | 7.891 | 321.16739 | [M+H]+1 | 4 |
| (1) | Myc_Var0000902 | Endo | C13 H18 N6 O3 | Lupinate_iso2 | 7.251 | 307.15175 | [M+H]+1 | 4 |
| (1) | Myc_Var0000903 | Endo | C15 H18 N4 O3 S | NA | 8.239 | 376.14339 | [M+ACN+H]+1 | 4 |
| (1) | Myc_Var0000905 | Endo | C15 H22 | Rulepidadiene B_iso6 | 12.471 | 203.1796 | [M+H]+1 | 4 |
| (1) | Myc_Var0000913 | Endo | C11 H14 O3 | Stagonosporyne G | 8.395 | 195.10166 | [M+H]+1 | 4 |
| (1) | Myc_Var0000919 | Endo | C15 H20 | NA | 12.986 | 201.16397 | [M+H]+1 | 4 |
| (1) | Myc_Var0000921 | Endo | C32 H57 N7 O4 | NA | 17.13 | 604.45738 | [M+H]+1 | 4 |
| (1) | Myc_Var0000924 | Endo | C14 H18 | NA | 10.602 | 187.14828 | [M+H]+1 | 4 |
| (1) | Myc_Var0000928 | Endo | C15 H24 O3 | Isotrichodiol_iso5 | 8.164 | 253.17999 | [M+H]+1 | 3 |
| (1) | Myc_Var0000930 | Endo | C14 H18 O | Penidienone | 8.837 | 203.14326 | [M+H]+1 | 4 |
| (2) | Med_Var0000001 | Exo | C34 H59 N3 O9 | Enniatin B1 | 14.758 | 671.45885 | [M+NH4]+1 | 1 |
| (2) | Med_Var0000002 | Exo | C35 H61 N3 O9 | Enniatin A1 | 15.556 | 685.47431 | [M+NH4]+1 | 1 |
| (2) | Med_Var0000004 | Exo | C33 H57 N3 O9 | Enniatin B | 14.117 | 640.41696 | [M+H]+1 | 1 |
| (2) | Med_Var0000005 | Exo | C38 H81 N2 O7 P3 | NA | 8.034 | 771.53285 | [M+H]+1 | 4 |
| (2) | Med_Var0000006 | Exo | C34 H73 N8 O10 P | NA | 8.081 | 785.52314 | [M+H]+1 | 4 |
| (2) | Med_Var0000010 | Exo | C32 H55 N3 O9 | Enniatin J2_iso1 | 13.636 | 643.42753 | [M+NH4]+1 | 4 |
| (2) | Med_Var0000015 | Exo | C35 H61 N3 O9 | Enniatin A1_iso1 | 15.245 | 685.47447 | [M+NH4]+1 | 4 |
| (2) | Med_Var0000025 | Exo | C32 H55 N3 O9 | Enniatin J2_iso2 | 13.99 | 643.42764 | [M+NH4]+1 | 4 |
| (2) | Med_Var0000028 | Exo | C32 H62 N10 O7 | NA | 14.815 | 699.49022 | [M+H]+1 | 4 |
| (2) | Med_Var0000030 | Exo | C38 H81 N2 O6 P3 | NA | 8.196 | 755.53778 | [M+H]+1 | 4 |
| (2) | Med_Var0000060 | Exo | C40 H76 N4 O6 P2 | NA | 7.927 | 771.53298 | [M+H]+1 | 4 |
| (2) | Med_Var0000090 | Exo | C36 H66 N4 O9 | NA | 14.609 | 699.49026 | [M+H]+1 | 4 |
| (2) | Med_Var0000096 | Exo | C35 H58 N O3 P3 | NA | 13.324 | 634.36727 | [M+H]+1 | 4 |
| (2) | Med_Var0000112 | Exo | C38 H79 N6 O2 P5 | NA | 8.082 | 807.50566 | [M+H]+1 | 4 |
| (2) | Med_Var0000119 | Exo | C34 H74 N4 O13 P2 | NA | 8.037 | 270.49871 | [M+3H]+3 | 4 |
| (2) | Med_Var0000122 | Exo | C38 H78 N2 O10 P2 | NA | 8.186 | 785.52376 | [M+H]+1 | 4 |
| (2) | Med_Var0000123 | Exo | C31 H53 N3 O9 | Enniatin J1 | 13.356 | 612.3859 | [M+H]+1 | 4 |
| (2) | Med_Var0000140 | Exo | C38 H81 N2 O7 P3 | NA | 8.147 | 771.53344 | [M+H]+1 | 4 |
| (2) | Med_Var0000144 | Exo | C30 H58 N10 O7 | NA | 13.637 | 671.45913 | [M+H]+1 | 4 |
| (2) | Med_Var0000175 | Exo | C37 H79 N4 O7 P3 | NA | 7.516 | 393.26599 | [M+2H]+2 | 4 |
| (2) | Med_Var0000185 | Exo | C33 H75 N6 O11 P3 | NA | 7.823 | 275.83037 | [M+3H]+3 | 4 |
| (2) | Med_Var0000209 | Exo | C35 H69 N10 O6 P | NA | 7.814 | 757.51814 | [M+H]+1 | 4 |
| (2) | Med_Var0000217 | Exo | C34 H74 N4 O12 P2 | NA | 8.197 | 265.16709 | [M+3H]+3 | 4 |
| (2) | Med_Var0000223 | Exo | C31 H65 N2 O10 P | NA | 13.355 | 657.44349 | [M+H]+1 | 4 |
| (2) | Med_Var0000247 | Exo | C33 H72 N6 O12 P2 | NA | 8.271 | 269.83055 | [M+3H]+3 | 4 |
| (2) | Med_Var0000289 | Exo | C33 H78 N10 O2 P4 | NA | 7.512 | 386.27105 | [M+2H]+2 | 4 |
| (2) | Med_Var0000307 | Exo | C34 H71 N10 O6 P3 | NA | 7.928 | 270.49873 | [M+3H]+3 | 4 |
| (2) | Med_Var0000312 | Exo | C21 H42 N4 O4 | NA | 7.041 | 415.32812 | [M+H]+1 | 4 |
| (2) | Med_Var0000357 | Exo | C40 H70 N4 O10 | NA | 8.084 | 384.26103 | [M+2H]+2 | 4 |
| (2) | Med_Var0000364 | Exo | C36 H74 N4 O12 | NA | 8.309 | 777.5205 | [M+Na]+1 | 4 |
| (2) | Med_Var0000368 | Exo | C34 H74 N6 O11 S2 | NA | 8.09 | 269.83645 | [M+3H]+3 | 4 |
| (2) | Med_Var0000429 | Exo | C34 H70 N4 O12 | NA | 7.513 | 727.50769 | [M+H]+1 | 4 |
| (2) | Med_Var0000449 | Exo | C33 H76 N10 O P4 | NA | 8.034 | 377.26586 | [M+2H]+2 | 4 |
| (2) | Med_Var0000475 | Exo | C12 H26 N2 O2 | NA | 4.745 | 231.207 | [M+H]+1 | 4 |
| (2) | Med_Var0000476 | Exo | C43 H72 N2 O9 S | NA | 8.036 | 265.17292 | [M+3H]+3 | 4 |
| (2) | Med_Var0000495 | Exo | C34 H66 N10 O13 | NA | 7.879 | 275.1681 | [M+3H]+3 | 4 |
| (2) | Med_Var0000497 | Exo | C35 H69 N10 O5 P | NA | 7.969 | 763.50505 | [M+Na]+1 | 4 |
| (2) | Med_Var0000506 | Exo | C36 H78 N O7 P5 | NA | 8.036 | 264.82323 | [M+3H]+3 | 4 |
| (2) | Med_Var0000547 | Exo | C33 H75 N6 O10 P3 | NA | 8.147 | 270.49875 | [M+3H]+3 | 4 |
| (2) | Med_Var0000645 | Exo | C32 H55 N3 O10 | Enniatin P1 | 12.023 | 659.42283 | [M+NH4]+1 | 4 |
| (2) | Med_Var0000646 | Exo | C36 H78 N O6 P5 | NA | 8.197 | 259.49159 | [M+3H]+3 | 4 |
| (2) | Med_Var0000653 | Exo | C29 H49 N3 O9 | NA | 12.374 | 601.3812 | [M+NH4]+1 | 4 |
| (2) | Med_Var0000661 | Exo | C38 H72 N4 O6 P2 | NA | 7.298 | 743.50262 | [M+H]+1 | 4 |
| (2) | Med_Var0000681 | Exo | C32 H52 N8 O5 | Plastin D | 12.95 | 629.41224 | [M+H]+1 | 4 |
| (2) | Med_Var0000691 | Exo | C34 H77 N2 O7 P5 | NA | 7.39 | 391.22876 | [M+2H]+2 | 4 |
| (2) | Med_Var0000693 | Exo | C45 H77 O4 P S2 | NA | 8.197 | 259.8413 | [M+3H]+3 | 4 |
| (2) | Med_Var0000724 | Exo | C31 H65 N7 O17 | NA | 7.823 | 270.15485 | [M+3H]+3 | 4 |
| (2) | Med_Var0000725 | Exo | C38 H76 N6 O3 P2 S2 | NA | 8.271 | 264.50481 | [M+3H]+3 | 4 |
| (2) | Med_Var0000732 | Exo | C21 H42 N4 O4 | NA | 6.929 | 437.31026 | [M+Na]+1 | 4 |
| (2) | Med_Var0000743 | Exo | C37 H82 N4 O5 P4 | NA | 7.343 | 394.26848 | [M+2H]+2 | 4 |
| (2) | Med_Var0000766 | Exo | C44 H76 N2 O5 S3 | NA | 7.821 | 405.25321 | [M+2H]+2 | 4 |
| (2) | Med_Var0000783 | Exo | C11 H19 N O3 | N-heptanoyl homoserine lactone | 7.246 | 214.14403 | [M+H]+1 | 4 |
| (2) | Med_Var0000799 | Exo | C34 H72 N8 O7 S | NA | 8.197 | 369.26842 | [M+2H]+2 | 4 |
| (2) | Med_Var0000811 | Exo | C35 H70 N6 O11 | NA | 8.275 | 376.26351 | [M+2H]+2 | 4 |
| (2) | Med_Var0000828 | Exo | C32 H55 N3 O10 | Enniatin P1 | 11.496 | 659.42281 | [M+NH4]+1 | 4 |
| (2) | Med_Var0000935 | Exo | C9 H10 O3 | (R)-3-methyl-1,3-dihydroisobenzofuran-4,6-diol | 6.702 | 167.07053 | [M+H]+1 | 4 |
| (2) | Med_Var0000951 | Exo | C32 H73 N9 O9 P2 | NA | 8.035 | 395.75784 | [M+2H]+2 | 4 |
| (2) | Med_Var0000973 | Exo | C45 H77 O5 P S2 | NA | 7.928 | 397.25568 | [M+2H]+2 | 4 |
| (2) | Med_Var0000977 | Exo | C30 H74 N10 O4 P4 S | NA | 7.807 | 398.2364 | [M+2H]+2 | 4 |
| (2) | Med_Var0001010 | Exo | C8 H15 N O3 | Hexanoylglycine | 4.795 | 174.11277 | [M+H]+1 | 3 |
| (2) | Med_Var0001110 | Exo | C37 H72 O14 | NA | 7.581 | 741.49805 | [M+H]+1 | 4 |
| (2) | Myc_Var0000001 | Endo | C35 H67 N2 O3 P3 | NA | 14.05 | 657.44291 | [M+H]+1 | 4 |
| (2) | Myc_Var0000002 | Endo | C34 H59 N3 O9 | Enniatin B1 | 14.559 | 654.43206 | [M+H]+1 | 1 |
| (2) | Myc_Var0000003 | Endo | C33 H57 N3 O9 | Enniatin B | 13.956 | 640.41628 | [M+H]+1 | 1 |
| (2) | Myc_Var0000004 | Endo | C35 H61 N3 O9 | Enniatin A1_iso1 | 15.13 | 668.44751 | [M+H]+1 | 4 |
| (2) | Myc_Var0000005 | Endo | C35 H61 N3 O9 | Enniatin A1 | 15.391 | 668.44827 | [M+H]+1 | 1 |
| (2) | Myc_Var0000007 | Endo | C34 H73 N8 O10 P | NA | 8.071 | 785.52287 | [M+H]+1 | 4 |
| (2) | Myc_Var0000008 | Endo | C31 H53 N3 O9 | Enniatin J1 | 13.43 | 629.41146 | [M+NH4]+1 | 4 |
| (2) | Myc_Var0000009 | Endo | C35 H75 N6 O10 P | NA | 8.032 | 771.53245 | [M+H]+1 | 4 |
| (2) | Myc_Var0000016 | Endo | C31 H60 N10 O7 | NA | 14.222 | 685.47433 | [M+H]+1 | 4 |
| (2) | Myc_Var0000018 | Endo | C38 H73 N2 O3 P3 | NA | 14.772 | 699.48999 | [M+H]+1 | 4 |
| (2) | Myc_Var0000025 | Endo | C37 H79 N4 O6 P3 | NA | 8.267 | 769.52789 | [M+H]+1 | 4 |
| (2) | Myc_Var0000032 | Endo | C32 H55 N3 O9 | Enniatin J2 | 13.611 | 626.40099 | [M+H]+1 | 4 |
| (2) | Myc_Var0000035 | Endo | C35 H58 N8 O5 | NA | 13.664 | 671.45875 | [M+H]+1 | 4 |
| (2) | Myc_Var0000053 | Endo | C35 H75 N6 O9 P | NA | 8.192 | 755.53793 | [M+H]+1 | 4 |
| (2) | Myc_Var0000062 | Endo | C36 H65 N3 O10 | NA | 13.132 | 700.47414 | [M+H]+1 | 4 |
| (2) | Myc_Var0000064 | Endo | C36 H63 N3 O9 | Enniatin A | 16.614 | 682.46353 | [M+H]+1 | 1 |
| (2) | Myc_Var0000087 | Endo | C40 H85 O4 P5 | NA | 8.006 | 785.52315 | [M+H]+1 | 4 |
| (2) | Myc_Var0000090 | Endo | C42 H83 O4 P5 | NA | 8.082 | 807.50557 | [M+H]+1 | 4 |
| (2) | Myc_Var0000106 | Endo | C32 H62 N O3 P5 | NA | 13.711 | 332.67795 | [M+2H]+2 | 4 |
| (2) | Myc_Var0000120 | Endo | C38 H78 N2 O10 P2 | NA | 8.184 | 785.52363 | [M+H]+1 | 4 |
| (2) | Myc_Var0000125 | Endo | C42 H62 N8 O10 | [L-Homoarginine2]nodularin | 7.881 | 280.49375 | [M+3H]+3 | 4 |
| (2) | Myc_Var0000126 | Endo | C33 H60 N4 O9 | NA | 13.424 | 657.44326 | [M+H]+1 | 4 |
| (2) | Myc_Var0000153 | Endo | C37 H74 N6 O5 P4 | NA | 8.268 | 269.83045 | [M+3H]+3 | 4 |
| (2) | Myc_Var0000217 | Endo | C38 H81 N2 O7 P3 | NA | 7.922 | 771.5335 | [M+H]+1 | 4 |
| (2) | Myc_Var0000259 | Endo | C35 H20 N4 O5 P2 | NA | 9.92 | 639.09805 | [M+H]+1 | 4 |
| (2) | Myc_Var0000263 | Endo | C29 H71 N10 O11 P | NA | 8.08 | 384.26084 | [M+2H]+2 | 4 |
| (2) | Myc_Var0000264 | Endo | C33 H75 N6 O10 P3 | NA | 8.031 | 405.24426 | [M+2H]+2 | 4 |
| (2) | Myc_Var0000268 | Endo | C31 H53 N3 O9 | Enniatin J1 | 12.987 | 612.38557 | [M+H]+1 | 4 |
| (2) | Myc_Var0000271 | Endo | C34 H74 N6 O11 S2 | NA | 8.087 | 269.83633 | [M+3H]+3 | 4 |
| (2) | Myc_Var0000278 | Endo | C36 H78 N6 O4 P4 | NA | 7.875 | 392.25821 | [M+2H]+2 | 4 |
| (2) | Myc_Var0000292 | Endo | C36 H74 N4 O13 | NA | 8.141 | 771.53344 | [M+H]+1 | 4 |
| (2) | Myc_Var0000294 | Endo | C39 H32 N2 O9 P2 | NA | 8.6 | 368.08722 | [M+2H]+2 | 4 |
| (2) | Myc_Var0000340 | Endo | C37 H65 N10 O9 P | NA | 7.818 | 413.24176 | [M+2H]+2 | 4 |
| (2) | Myc_Var0000371 | Endo | C38 H78 O14 S2 | NA | 7.881 | 275.168 | [M+3H]+3 | 4 |
| (2) | Myc_Var0000384 | Endo | C34 H74 N4 O12 P2 | NA | 8.193 | 397.24683 | [M+2H]+2 | 4 |
| (2) | Myc_Var0000441 | Endo | C31 H70 N10 O11 S | NA | 8.268 | 264.50473 | [M+3H]+3 | 4 |
| (2) | Myc_Var0000447 | Endo | C26 H30 N2 O9 P4 | NA | 9.771 | 639.09788 | [M+H]+1 | 4 |
| (2) | Myc_Var0000481 | Endo | C34 H24 O9 P2 | NA | 8.901 | 639.09807 | [M+H]+1 | 4 |
| (2) | Myc_Var0000493 | Endo | C40 H70 N4 O9 | NA | 8.268 | 376.26345 | [M+2H]+2 | 4 |
| (2) | Myc_Var0000519 | Endo | C36 H20 N4 O7 S | NA | 9.482 | 653.11359 | [M+H]+1 | 4 |
| (2) | Myc_Var0000559 | Endo | C35 H73 N6 O9 P | NA | 8.031 | 377.26568 | [M+2H]+2 | 4 |
| (2) | Myc_Var0000580 | Endo | C31 H59 N9 O8 | NA | 12.727 | 686.45874 | [M+H]+1 | 4 |
| (2) | Myc_Var0000656 | Endo | NA | NA | 8.031 | 265.17282 | [M+3H]+3 | 5 |
| (2) | Myc_Var0000666 | Endo | C35 H20 N4 O5 P2 | NA | 9.586 | 639.098 | [M+H]+1 | 4 |
| (2) | Myc_Var0000674 | Endo | C33 H71 N6 O10 P | NA | 7.385 | 765.48397 | [M+Na]+1 | 4 |
| (2) | Myc_Var0000740 | Endo | C26 H30 N2 O9 P4 | NA | 8.769 | 639.09802 | [M+H]+1 | 4 |
| (2) | Myc_Var0000760 | Endo | C33 H68 N6 O13 | NA | 7.43 | 757.49268 | [M+H]+1 | 4 |
| (2) | Myc_Var0000764 | Endo | C30 H22 O16 | NA | 8.401 | 639.09814 | [M+H]+1 | 4 |
| (2) | Myc_Var0000771 | Endo | C52 H64 N4 O4 | NA | 7.818 | 270.50449 | [M+3H]+3 | 4 |
| (2) | Myc_Var0000784 | Endo | C30 H22 O16 | NA | 9.248 | 639.09803 | [M+H]+1 | 4 |
| (2) | Myc_Var0000789 | Endo | C31 H18 N4 O12 | NA | 8.525 | 639.09818 | [M+H]+1 | 4 |
| (2) | Myc_Var0000867 | Endo | C54 H45 N4 O27 P3 | NA | 10.226 | 638.08168 | [M+2H]+2 | 4 |
| (3) | Med_Var0000062 | Exo | C6 H8 O2 | 2-(Methoxymethyl)furan | 2.524 | 113.06002 | [M+H]+1 | 3 |
| (3) | Myc_Var0000083 | Endo | C26 H52 N O7 P | 1-Oleoylglycerophosphocholine | 13.968 | 522.35569 | [M+H]+1 | 4 |
| (3) | Myc_Var0000509 | Endo | C24 H46 N7 O5 P | NA | 13.952 | 544.33752 | [M+H]+1 | 4 |
| (3) | Myc_Var0000761 | Endo | C15 H33 N5 O4 P4 S | NA | 4.457 | 504.12857 | [M+H]+1 | 4 |
| (3) | Myc_Var0000855 | Endo | C26 H51 N5 O4 | NA | 5.958 | 498.40165 | [M+H]+1 | 4 |

Table S2: Strain-specific endometabolites biomarkers identified through Upset plot. Each feature is described by: a producer (Strain_specific_feature), an identifier (**Variable_ID**); a retention time in minutes (**RT_min**); a mass-to-charge ratio associated with an adduct (**m/z_of_adduct** and **Adduct**); a putative molecular formula and name (**Putative_formula** and **Putative_name**) as assigned by Compound Discoverer software; and a confidence level of identification (Schymanski et al., 2014).

| Strain_specific_ feature | Variable_ID | Putative_formula | Putative_name | RT_ min | m/z_of_adduct | Adduct | Confidence_level_of_ identification |
| --- | --- | --- | --- | --- | --- | --- | --- |
| Fav498 | Myc_Var0000537 | C37 H79 N4 O7 P3 | NA | 7.512 | 393.26607 | [M+2H]+2 | 4 |
| Fav498 | Myc_Var0000566 | C40 H72 N4 O10 | NA | 8.374 | 769.52892 | [M+H]+1 | 4 |
| Fav498 | Myc_Var0000585 | C26 H26 O7 | Effphenol A | 8.369 | 451.17543 | [M+H]+1 | 4 |
| Fav498 | Myc_Var0000596 | C42 H62 N8 O9 | Anabaenopeptin SA3_iso3 | 8.181 | 412.23968 | [M+2H]+2 | 4 |
| Fav498 | Myc_Var0000630 | C27 H32 O8 | Isoterreulactone A | 9.356 | 485.21737 | [M+H]+1 | 4 |
| Fav498 | Myc_Var0000685 | C28 H38 O6 | Blazeispirol V1 | 12.883 | 471.27444 | [M+H]+1 | 4 |
| Fav498 | Myc_Var0000770 | C35 H75 N8 O10 P | NA | 8.493 | 799.53874 | [M+H]+1 | 4 |
| Fav498 | Myc_Var0000881 | C41 H73 N O9 | Bacteriohopanepentol ether | 16.379 | 724.53558 | [M+H]+1 | 4 |
| Fav498 | Myc_Var0000889 | C28 H40 O6 | 11-hydroxychevalone C | 12.391 | 473.29007 | [M+H]+1 | 4 |
| Fcu337 | Myc_Var0000119 | C19 H27 N O9 | NA | 6.237 | 414.17582 | [M+H]+1 | 4 |
| Fcu337 | Myc_Var0000175 | C19 H39 N O2 | NA | 13.982 | 314.30538 | [M+H]+1 | 4 |
| Fcu337 | Myc_Var0000233 | C29 H24 O9 | Aschernaphthopyrone B | 6.239 | 259.07843 | [M+2H]+2 | 4 |
| Fcu337 | Myc_Var0000306 | C19 H22 O8 | Aigialospirol | 6.238 | 379.13894 | [M+H]+1 | 4 |
| Fcu337 | Myc_Var0000317 | C19 H26 O8 | 7,8-Dihydroxycalonectrin | 6.613 | 383.17018 | [M+H]+1 | 3 |
| Fcu337 | Myc_Var0000332 | C18 H22 O6 | Tetrodecamycin | 6.613 | 317.13843 | [M+H-H2O]+1 | 4 |
| Fcu337 | Myc_Var0000345 | C24 H18 O8 | Sinaticin | 6.237 | 435.10543 | [M+H]+1 | 4 |
| Fcu337 | Myc_Var0000363 | C21 H41 N O2 | NA | 13.966 | 340.3211 | [M+H]+1 | 4 |
| Fcu337 | Myc_Var0000397 | NA | NA | 6.239 | 416.12307 | [M+2H]+2 | 5 |
| Fcu337 | Myc_Var0000401 | C17 H22 O8 | Fusarenon-X | 4.53 | 355.13887 | [M+H]+1 | 1 |
| Fcu337 | Myc_Var0000473 | C17 H42 N9 O6 P S2 | NA | 8.756 | 564.25216 | [M+H]+1 | 4 |
| Fcu337 | Myc_Var0000513 | C26 H32 N2 O2 S3 | NA | 6.239 | 251.08973 | [M+2H]+2 | 4 |
| Fcu337 | Myc_Var0000565 | C23 H26 N5 O7 P | NA | 4.532 | 258.58648 | [M+2H]+2 | 4 |
| Fcu337 | Myc_Var0000571 | C17 H20 O5 | PF1092B_iso3 | 6.613 | 305.13849 | [M+H]+1 | 3 |
| Fcu337 | Myc_Var0000574 | C18 H20 N6 O7 | NA | 7.069 | 433.14714 | [M+H]+1 | 4 |
| Fcu337 | Myc_Var0000599 | C29 H26 O8 | Demethoxyisogemichalcone C | 6.613 | 252.08888 | [M+2H]+2 | 4 |
| Fcu337 | Myc_Var0000639 | C22 H45 N O2 | N-(2-hydroxyethyl)icosanamide | 15.228 | 356.3525 | [M+H]+1 | 4 |
| Fcu337 | Myc_Var0000652 | C21 H46 N3 O8 P3 S2 | NA | 9.237 | 313.60334 | [M+2H]+2 | 4 |
| Fcu337 | Myc_Var0000720 | C19 H24 O8 | 3,15-Acetyldeoxynivalenol | 6.789 | 403.13646 | [M+Na]+1 | 3 |
| Fcu337 | Myc_Var0000732 | C19 H28 O9 | Ghanamycin B | 4.718 | 423.16265 | [M+Na]+1 | 4 |
| Fcu337 | Myc_Var0000786 | C19 H24 O9 | 4,15-Diacetylnivalenol | 6.239 | 397.14965 | [M+H]+1 | 4 |
| Fcu337 | Myc_Var0000803 | C19 H24 O6 | Gibberellin A1_iso2 | 5.481 | 349.16475 | [M+H]+1 | 4 |
| Fcu337 | Myc_Var0000833 | C27 H24 O7 | BE-24566B_iso2 | 5.16 | 231.08361 | [M+2H]+2 | 4 |
| Fcu337 | Myc_Var0000849 | C15 H18 O | 1-Hydroxymethyl-4-methyl-7-isopropenylaz_iso3 | 13.482 | 215.14315 | [M+H]+1 | 4 |
| Fcu337 | Myc_Var0000863 | C14 H14 O2 | 7-(1-hydroxyethyl)-4-methyl-1-azulenecarboxaldehyde_iso3 | 13.482 | 247.13301 | [M+H+MeOH]+1 | 4 |
| Fcu337 | Myc_Var0000870 | C23 H45 N O2 | NA | 15.084 | 368.35258 | [M+H]+1 | 4 |
| Fcu337 | Myc_Var0000880 | C23 H24 O10 | Pectolinarigenin 7-rhamnoside | 7.784 | 461.14204 | [M+H]+1 | 4 |
| Fcu337 | Myc_Var0000894 | C15 H20 O | Anaephene C_iso6 | 14.265 | 217.1588 | [M+H]+1 | 4 |
| Fcu337 | Myc_Var0000904 | C14 H14 O2 | 2,4,6,8,10,12-tetradeca-1,14-hexenedial | 6.669 | 215.10677 | [M+H]+1 | 4 |
| Fcu337 | Myc_Var0000907 | C22 H24 N6 O7 S | Penilumamide | 6.127 | 259.07853 | [M+2H]+2 | 4 |
| Fcu337 | Myc_Var0000911 | C15 H16 O5 | Conosilane A | 6.239 | 277.10724 | [M+H]+1 | 4 |
| Fcu337 | Myc_Var0000920 | C21 H39 N O2 | NA | 13.087 | 338.30557 | [M+H]+1 | 4 |
| Fcu337 | Myc_Var0000925 | C15 H14 O4 | (S)-2,5-dihydroxy-2-methyl-1,2,3,4-tetrahydroanthracene-9,10-dione | 6.238 | 259.09644 | [M+H]+1 | 4 |
| Fgr149 | Myc_Var0000187 | C32 H44 O14 P2 | NA | 5.565 | 358.11723 | [M+2H]+2 | 4 |
| Fgr149 | Myc_Var0000215 | C27 H22 O7 | Naphthacemycin B1 | 5.561 | 230.07567 | [M+2H]+2 | 4 |
| Fgr149 | Myc_Var0000253 | C15 H18 O5 | Hirsutenol D | 5.565 | 261.11216 | [M+H-H2O]+1 | 3 |
| Fgr149 | Myc_Var0000298 | NA | NA | 5.564 | 350.12865 | [M+2H]+2 | 5 |
| Fgr149 | Myc_Var0000352 | C28 H19 N5 O2 | NA | 3.074 | 229.58372 | [M+2H]+2 | 4 |
| Fgr149 | Myc_Var0000391 | C10 H19 N O5 | NA | 1.576 | 234.13368 | [M+H]+1 | 4 |
| Fgr149 | Myc_Var0000393 | C17 H26 O2 | PI-200_iso1 | 11.222 | 263.20064 | [M+H]+1 | 4 |
| Fgr149 | Myc_Var0000430 | C17 H22 O7 | 15-Acetyldeoxynivalenol | 5.565 | 361.12582 | [M+Na]+1 | 1 |
| Fgr149 | Myc_Var0000432 | C20 H30 N2 O5 S2 | Bilain B | 5.565 | 222.08694 | [M+2H]+2 | 4 |
| Fgr149 | Myc_Var0000434 | C20 H26 N6 O4 P2 | NA | 5.567 | 239.08099 | [M+2H]+2 | 4 |
| Fgr149 | Myc_Var0000454 | NA | NA | 7.175 | 413.21064 | [M+H]+1 | 5 |
| Fgr149 | Myc_Var0000496 | C18 H29 N O4 S | NA | 7.068 | 356.18913 | [M+H]+1 | 4 |
| Fgr149 | Myc_Var0000506 | C14 H34 N5 O3 P3 | NA | 7.374 | 414.19467 | [M+H]+1 | 4 |
| Fgr149 | Myc_Var0000538 | C15 H22 N6 O2 | NA | 9.896 | 319.188 | [M+H]+1 | 4 |
| Fgr149 | Myc_Var0000627 | C20 H31 N O6 S | NA | 6.797 | 396.18407 | [M+H-H2O]+1 | 4 |
| Fgr149 | Myc_Var0000668 | C15 H24 O2 | 12,13-Epoxy-9,10-trichoene-2-ol_iso4 | 10.365 | 237.18508 | [M+H]+1 | 3 |
| Fgr149 | Myc_Var0000676 | C9 H17 N O2 | (E)-3-hydroxy-2,4-dimethylhept-4-enamide | 3.633 | 172.13332 | [M+H]+1 | 4 |
| Fgr149 | Myc_Var0000689 | C20 H29 N5 O | NA | 9.382 | 356.24326 | [M+H]+1 | 4 |
| Fgr149 | Myc_Var0000699 | C15 H29 N4 O4 P | NA | 9.382 | 361.19862 | [M+H]+1 | 4 |
| Fgr149 | Myc_Var0000715 | C15 H22 O2 | 12,13-Epoxytrichothec-9-ene_iso5 | 7.929 | 235.16937 | [M+H]+1 | 3 |
| Fgr149 | Myc_Var0000739 | C17 H26 O2 | PI-200_iso3 | 10.844 | 263.20061 | [M+H]+1 | 4 |
| Fgr149 | Myc_Var0000746 | C21 H28 O | NA | 15.478 | 297.22136 | [M+H]+1 | 4 |
| Fgr149 | Myc_Var0000791 | C15 H18 O4 | Heterocornol O_iso2 | 5.687 | 263.12792 | [M+H]+1 | 3 |
| Fgr149 | Myc_Var0000848 | C35 H73 N8 O12 P | NA | 8.164 | 829.51327 | [M+H]+1 | 4 |
| Fgr149 | Myc_Var0000865 | C18 H34 O2 | cis-Vaccenate | 14.697 | 283.26334 | [M+H]+1 | 4 |
| Fgr149 | Myc_Var0000877 | C17 H26 O5 | Trichodermarin F_iso3 | 7.186 | 311.18535 | [M+H]+1 | 3 |
| Fgr149 | Myc_Var0000888 | C12 H23 N O4 | 3-hydroxy-N-(1-hydroxy-3-methylpentan-2-yl)-5-oxohexanamide_iso5 | 5.658 | 246.17013 | [M+H]+1 | 4 |
| Fgr149 | Myc_Var0000929 | C15 H18 | Guaiazulene_iso2 | 14.373 | 199.14832 | [M+H]+1 | 4 |
| Fla509 | Myc_Var0000164 | C12 H16 O3 | F-11334-B2 | 9.466 | 209.11731 | [M+H]+1 | 4 |
| Fla509 | Myc_Var0000272 | C16 H17 N O2 | 3-acetonylidene-7-prenylindolin-2-one | 7.505 | 273.1598 | [M+NH4]+1 | 4 |
| Fla509 | Myc_Var0000309 | C18 H19 N O2 | NA | 8.23 | 299.17538 | [M+NH4]+1 | 4 |
| Fla509 | Myc_Var0000806 | C28 H42 O3 | 5a,8a-Epidioxyergosta-6,9(11),22-trien-3_iso2 | 15.064 | 427.32107 | [M+H]+1 | 4 |
| Fla509 | Myc_Var0000899 | C30 H50 O3 | Soyasapogenol B | 13.635 | 481.36552 | [M+Na]+1 | 3 |
| Fpo073 | Myc_Var0000236 | C27 H57 N3 O2 | NA | 12.383 | 456.45254 | [M+H]+1 | 4 |
| Fpo073 | Myc_Var0000256 | C18 H18 O8 | Isosecosterigmatocystin | 8.583 | 363.1074 | [M+H]+1 | 3 |
| Fpo073 | Myc_Var0000546 | NA | NA | 4.856 | 411.65849 | [M+2H]+2 | 5 |
| Fpo073 | Myc_Var0000744 | C21 H34 O3 | NA | 13.983 | 317.24763 | [M+H-H2O]+1 | 4 |
| Fsp101 | Myc_Var0000072 | C17 H18 O4 | Trypethelon-methylether_iso1 | 10.392 | 287.12783 | [M+H]+1 | 4 |
| Fsp101 | Myc_Var0000075 | C19 H22 O6 | Gibberellin A3 | 10.392 | 347.14897 | [M+H]+1 | 4 |
| Fsp101 | Myc_Var0000078 | C14 H14 O2 | 7-(1-hydroxyethyl)-4-methyl-1-azulenecarboxaldehyde_iso1 | 10.392 | 215.1067 | [M+H]+1 | 4 |
| Fsp101 | Myc_Var0000079 | C21 H26 O8 | Lunatoic acid D | 10.392 | 407.17015 | [M+H]+1 | 4 |
| Fsp101 | Myc_Var0000080 | C14 H12 O | cis-Stilbeneoxide | 10.391 | 197.09623 | [M+H]+1 | 4 |
| Fsp101 | Myc_Var0000086 | C14 H14 O2 | 7-(1-hydroxyethyl)-4-methyl-1-azulenecarboxaldehyde_iso2 | 9.17 | 215.1067 | [M+H]+1 | 4 |
| Fsp101 | Myc_Var0000135 | C15 H16 O3 | 7-(1,2-Dihydroxy-1-methylethyl)-4-methylazulene-1-carbaldehyde_iso1 | 9.17 | 245.11726 | [M+H]+1 | 4 |
| Fsp101 | Myc_Var0000136 | C17 H18 O5 | Acremeremophilane N | 9.17 | 335.14897 | [M+H+MeOH]+1 | 4 |
| Fsp101 | Myc_Var0000161 | C16 H16 O3 | 7,4'-Dihydroxy-8-methylflavan_iso1 | 10.391 | 257.1173 | [M+H]+1 | 4 |
| Fsp101 | Myc_Var0000162 | C13 H12 O | NA | 9.17 | 185.09618 | [M+H]+1 | 4 |
| Fsp101 | Myc_Var0000165 | C15 H16 O3 | 7-(1,2-Dihydroxy-1-methylethyl)-4-methylazulene-1-carbaldehyde_iso2 | 10.391 | 245.11733 | [M+H]+1 | 4 |
| Fsp101 | Myc_Var0000171 | C24 H34 O8 | Maunakeanolin | 9.793 | 473.21438 | [M+Na]+1 | 4 |
| Fsp101 | Myc_Var0000183 | NA | NA | 10.395 | 528.21211 | [M+2H]+2 | 5 |
| Fsp101 | Myc_Var0000191 | C28 H42 N2 O10 P2 | NA | 10.392 | 315.12284 | [M+2H]+2 | 4 |
| Fsp101 | Myc_Var0000204 | C19 H23 N O5 | Trichosorbicillin A | 9.17 | 346.16504 | [M+H]+1 | 4 |
| Fsp101 | Myc_Var0000221 | C13 H12 O | NA | 10.392 | 185.09623 | [M+H]+1 | 4 |
| Fsp101 | Myc_Var0000235 | NA | NA | 9.17 | 294.11757 | [M+2H]+2 | 5 |
| Fsp101 | Myc_Var0000245 | C16 H18 O4 | Yukonin_iso1 | 9.17 | 275.1279 | [M+H]+1 | 4 |
| Fsp101 | Myc_Var0000255 | C13 H12 | NA | 10.392 | 169.10129 | [M+H]+1 | 4 |
| Fsp101 | Myc_Var0000261 | C19 H20 O5 | NA | 10.392 | 329.13855 | [M+H]+1 | 4 |
| Fsp101 | Myc_Var0000273 | C21 H26 O7 | Pilatin | 10.973 | 391.17526 | [M+H]+1 | 4 |
| Fsp101 | Myc_Var0000285 | C13 H12 | NA | 9.17 | 169.10127 | [M+H]+1 | 4 |
| Fsp101 | Myc_Var0000286 | C19 H24 O7 | Neoabyssomicin A2 | 9.17 | 365.15967 | [M+H]+1 | 4 |
| Fsp101 | Myc_Var0000295 | C12 H10 O | NA | 9.17 | 203.10677 | [M+H+MeOH]+1 | 4 |
| Fsp101 | Myc_Var0000301 | C16 H16 O3 | 7,4'-Dihydroxy-8-methylflavan_iso2 | 9.17 | 257.11733 | [M+H]+1 | 4 |
| Fsp101 | Myc_Var0000305 | C32 H41 N O4 | Phomopsichalasin | 11.432 | 504.30891 | [M+H]+1 | 4 |
| Fsp101 | Myc_Var0000315 | C7 H8 O2 | (-)-hepta-4,6-diyne-2,3-diol_iso1 | 10.974 | 125.0599 | [M+H]+1 | 4 |
| Fsp101 | Myc_Var0000335 | C16 H18 O4 | Yukonin_iso2 | 10.392 | 275.12795 | [M+H]+1 | 4 |
| Fsp101 | Myc_Var0000342 | NA | NA | 9.172 | 486.2015 | [M+2H]+2 | 5 |
| Fsp101 | Myc_Var0000362 | C19 H28 O5 | Cytosporin W_iso2 | 10.228 | 337.20096 | [M+H]+1 | 4 |
| Fsp101 | Myc_Var0000366 | C47 H80 N5 O5 P3 | NA | 12.065 | 888.54305 | [M+H]+1 | 4 |
| Fsp101 | Myc_Var0000372 | C28 H40 O5 | 3beta,14beta-dihydroxy-6-oxo-A-norergosta-7,22-diene-4-oic acid delta-lactone | 13.481 | 479.27684 | [M+Na]+1 | 4 |
| Fsp101 | Myc_Var0000377 | C17 H18 O3 | 4-Hydrocinnamoyl-2,2,5-trimethyl-4-cyclopentene-1,3-dione | 10.973 | 271.133 | [M+H]+1 | 4 |
| Fsp101 | Myc_Var0000394 | C17 H20 O4 | 7alpha-Acetoxydehydrobotrydienal_iso1 | 10.476 | 289.14356 | [M+H]+1 | 4 |
| Fsp101 | Myc_Var0000408 | C15 H14 O | Dihydrochalcone | 10.974 | 211.11191 | [M+H]+1 | 4 |
| Fsp101 | Myc_Var0000409 | C26 H36 O9 | Terreusterpene C | 10.972 | 515.22528 | [M+Na]+1 | 4 |
| Fsp101 | Myc_Var0000410 | C28 H34 N4 O6 | NA | 10.912 | 540.28068 | [M+NH4]+1 | 4 |
| Fsp101 | Myc_Var0000419 | C24 H34 O8 | Luminacin B1/B2 | 10.476 | 451.23288 | [M+H]+1 | 4 |
| Fsp101 | Myc_Var0000424 | C29 H41 N O4 | Lobosamide C | 12.719 | 468.3088 | [M+H]+1 | 4 |
| Fsp101 | Myc_Var0000477 | C19 H22 O5 | Gibberellin A5 | 10.973 | 331.1542 | [M+H]+1 | 4 |
| Fsp101 | Myc_Var0000502 | C27 H34 O9 | Verrucarin A | 9.735 | 503.22536 | [M+H]+1 | 4 |
| Fsp101 | Myc_Var0000516 | C17 H20 O5 | PF1092B_iso2 | 10.393 | 305.13855 | [M+H]+1 | 4 |
| Fsp101 | Myc_Var0000520 | C20 H25 N5 O4 | NA | 5.139 | 400.19682 | [M+H]+1 | 4 |
| Fsp101 | Myc_Var0000527 | C19 H24 O6 | Gibberellin A1_iso1 | 10.476 | 349.16479 | [M+H]+1 | 4 |
| Fsp101 | Myc_Var0000578 | C24 H30 O8 | Estroneglucuronide | 8.361 | 447.19928 | [M+H]+1 | 4 |
| Fsp101 | Myc_Var0000581 | C31 H39 N O4 | Pyreudione B_iso3 | 11.134 | 490.293 | [M+H]+1 | 4 |
| Fsp101 | Myc_Var0000591 | C19 H28 O7 | Inonoalliacane C | 5.814 | 391.17289 | [M+Na]+1 | 4 |
| Fsp101 | Myc_Var0000610 | C43 H78 N5 O12 P | NA | 12.127 | 888.54313 | [M+H]+1 | 4 |
| Fsp101 | Myc_Var0000628 | C20 H30 O4 | Aphidicolin A54 | 11.731 | 357.20385 | [M+Na]+1 | 3 |
| Fsp101 | Myc_Var0000694 | C14 H14 O2 | 2,4,6,8,10,12-tetradeca-1,14-hexenedial | 10.918 | 215.10678 | [M+H]+1 | 4 |
| Fsp101 | Myc_Var0000711 | C17 H18 O4 | Trypethelon-methylether_iso2 | 10.916 | 287.12799 | [M+H]+1 | 4 |
| Fsp101 | Myc_Var0000713 | C17 H22 O6 | (S)-7-hydroxytrichothecolone acetate | 9.17 | 323.14905 | [M+H]+1 | 4 |
| Fsp101 | Myc_Var0000718 | C25 H49 N O4 | N-[(1R)-2-hydroxy-1-methoxy-methyl ethyl]-(4E,7S)-7-methoxy-4-eicosenamide | 14.331 | 428.37373 | [M+H]+1 | 4 |
| Fsp101 | Myc_Var0000721 | C22 H28 O8 | Emerone A | 10.918 | 421.18602 | [M+H]+1 | 4 |
| Fsp101 | Myc_Var0000742 | C29 H24 O6 | Dichamanetin | 10.474 | 235.08615 | [M+2H]+2 | 4 |
| Fsp101 | Myc_Var0000767 | C28 H40 O5 | Blazeispirol Z1 | 13.689 | 479.27703 | [M+Na]+1 | 4 |
| Fsp101 | Myc_Var0000792 | C20 H32 N6 O2 | NA | 11.738 | 389.26646 | [M+H]+1 | 4 |
| Fsp101 | Myc_Var0000795 | C43 H83 N3 O8 P4 S | NA | 12.065 | 463.7494 | [M+2H]+2 | 4 |
| Fsp101 | Myc_Var0000804 | C15 H16 O2 | Variabilone_iso1 | 9.792 | 229.12245 | [M+H]+1 | 4 |
| Fsp101 | Myc_Var0000824 | C27 H38 O9 | 11-Hydroxyprogesterone11-glucuronide | 11.518 | 529.2411 | [M+Na]+1 | 4 |
| Fsp101 | Myc_Var0000830 | C17 H20 O4 | 7alpha-Acetoxydehydrobotrydienal_iso2 | 9.791 | 289.14357 | [M+H]+1 | 4 |
| Fsp101 | Myc_Var0000838 | C15 H20 N6 O | NA | 10.624 | 301.17752 | [M+H]+1 | 4 |
| Fsp101 | Myc_Var0000875 | C15 H12 O8 S | 5,7,4'-Trihydroxyflavanone 7-sulfate | 7.02 | 353.03279 | [M+H]+1 | 4 |
| Fsp101 | Myc_Var0000884 | C26 H30 O9 | Astellolide B | 8.89 | 487.19408 | [M+H]+1 | 4 |
| Fsp101 | Myc_Var0000887 | C19 H34 N2 O6 | Bengamide F' | 12.36 | 387.25089 | [M+H]+1 | 4 |
| Fsp101 | Myc_Var0000893 | C17 H26 O4 | Cytosporone T_iso3 | 10.195 | 317.17244 | [M+Na]+1 | 4 |
| Ftr521 | Myc_Var0000071 | C22 H27 N O7 | Tirandamycin A_iso1 | 8.852 | 440.16786 | [M+Na]+1 | 4 |
| Ftr521 | Myc_Var0000076 | C22 H27 N O7 | Tirandamycin A_iso2 | 8.631 | 440.16792 | [M+Na]+1 | 4 |
| Ftr521 | Myc_Var0000088 | C22 H27 N O7 | Tirandamycin A_iso3 | 8.952 | 418.18602 | [M+H]+1 | 4 |
| Ftr521 | Myc_Var0000091 | C22 H27 N O7 | Tirandamycin A_iso4 | 8.793 | 440.16785 | [M+Na]+1 | 4 |
| Ftr521 | Myc_Var0000099 | C22 H27 N O7 | Cephalimysin M | 9.021 | 418.18604 | [M+H]+1 | 4 |
| Ftr521 | Myc_Var0000201 | C16 H18 O2 | 1,4-diphenyl-2,3-butanediol | 11.109 | 275.16425 | [M+H+MeOH]+1 | 4 |
| Ftr521 | Myc_Var0000232 | C22 H27 N O5 | Streptoglyceride C | 9.778 | 386.19626 | [M+H]+1 | 4 |
| Ftr521 | Myc_Var0000242 | C19 H32 N2 O9 S2 | NA | 8.802 | 249.0836 | [M+2H]+2 | 4 |
| Ftr521 | Myc_Var0000281 | C35 H58 N4 O7 | NA | 12.744 | 647.43806 | [M+H]+1 | 4 |
| Ftr521 | Myc_Var0000290 | C22 H27 N O6 | Tirandamycin D_iso1 | 8.87 | 402.19145 | [M+H]+1 | 4 |
| Ftr521 | Myc_Var0000344 | C22 H27 N O6 | Tirandamycin D_iso2 | 9.457 | 402.19138 | [M+H]+1 | 4 |
| Ftr521 | Myc_Var0000379 | C26 H32 N2 P4 | NA | 8.953 | 249.08361 | [M+2H]+2 | 4 |
| Ftr521 | Myc_Var0000386 | C19 H32 N2 O9 S2 | NA | 9.021 | 249.08363 | [M+2H]+2 | 4 |
| Ftr521 | Myc_Var0000451 | C14 H14 O | NA | 8.852 | 199.11191 | [M+H]+1 | 4 |
| Ftr521 | Myc_Var0000453 | C14 H14 O | NA | 8.952 | 199.11193 | [M+H]+1 | 4 |
| Ftr521 | Myc_Var0000471 | C20 H32 O3 | Decatamariic acid_iso1 | 15.103 | 321.24258 | [M+H]+1 | 3 |
| Ftr521 | Myc_Var0000476 | C22 H27 N O6 | Tirandamycin D_iso3 | 9.406 | 402.19142 | [M+H]+1 | 4 |
| Ftr521 | Myc_Var0000491 | C21 H19 N O4 | NA | 8.951 | 382.16524 | [M+H+MeOH]+1 | 4 |
| Ftr521 | Myc_Var0000504 | C19 H32 N2 O9 S2 | NA | 8.851 | 249.08359 | [M+2H]+2 | 4 |
| Ftr521 | Myc_Var0000507 | C19 H30 O3 | Xylarinorditerpene K | 13.017 | 307.2268 | [M+H]+1 | 4 |
| Ftr521 | Myc_Var0000511 | C35 H61 N6 O5 P | NA | 12.673 | 677.44822 | [M+H]+1 | 4 |
| Ftr521 | Myc_Var0000528 | C15 H14 O2 | 7-Hydroxyflavan_iso3 | 8.851 | 259.13305 | [M+H+MeOH]+1 | 4 |
| Ftr521 | Myc_Var0000562 | C14 H14 O | NA | 8.63 | 199.11196 | [M+H]+1 | 4 |
| Ftr521 | Myc_Var0000572 | C15 H14 O2 | 7-Hydroxyflavan_iso4 | 8.952 | 259.13308 | [M+H+MeOH]+1 | 4 |
| Ftr521 | Myc_Var0000606 | C22 H23 N O5 | Yaequinolone E | 8.852 | 382.16515 | [M+H]+1 | 4 |
| Ftr521 | Myc_Var0000619 | C15 H14 O2 | 7-Hydroxyflavan_iso5 | 8.63 | 259.1331 | [M+H+MeOH]+1 | 4 |
| Ftr521 | Myc_Var0000620 | C22 H34 O3 | Penostatin D | 16.252 | 347.25829 | [M+H]+1 | 4 |
| Ftr521 | Myc_Var0000678 | C21 H19 N O4 | NA | 9.013 | 382.16528 | [M+H+MeOH]+1 | 4 |
| Ftr521 | Myc_Var0000698 | C23 H23 N5 O3 | N6-Formyl-roquefortin C | 8.472 | 440.16827 | [M+Na]+1 | 4 |
| Ftr521 | Myc_Var0000708 | C15 H14 O2 | 7-Hydroxyflavan_iso6 | 8.794 | 259.13311 | [M+H+MeOH]+1 | 4 |
| Ftr521 | Myc_Var0000734 | C16 H20 O3 | Lahorenoic acid A | 11.315 | 261.14867 | [M+H]+1 | 4 |
| Ftr521 | Myc_Var0000743 | C22 H29 N O6 | Torrubiellone B | 8.53 | 404.20704 | [M+H]+1 | 4 |
| Ftr521 | Myc_Var0000753 | C21 H23 N O5 | Azaspirene | 8.863 | 370.16526 | [M+H]+1 | 4 |
| Ftr521 | Myc_Var0000765 | C28 H55 N8 O6 P | NA | 11.566 | 631.40655 | [M+H]+1 | 4 |
| Ftr521 | Myc_Var0000766 | C37 H61 N3 O8 | NA | 12.982 | 676.45304 | [M+H]+1 | 4 |
| Ftr521 | Myc_Var0000772 | C35 H52 N8 O5 S | NA | 12.712 | 349.19608 | [M+2H]+2 | 4 |
| Ftr521 | Myc_Var0000778 | C20 H30 O3 | ent-7alpha-Hydroxykaur-16-en-19-Oate | 13.848 | 319.22695 | [M+H]+1 | 3 |
| Ftr521 | Myc_Var0000797 | C33 H52 N4 O7 | NA | 11.215 | 617.39085 | [M+H]+1 | 4 |
| Ftr521 | Myc_Var0000802 | C21 H23 N O4 | Cuevaene D | 9.777 | 354.17026 | [M+H]+1 | 4 |
| Ftr521 | Myc_Var0000812 | C20 H32 O3 | Decatamariic acid_iso2 | 14.222 | 321.24259 | [M+H]+1 | 3 |
| Ftr521 | Myc_Var0000839 | C22 H34 O3 | Penostatin D | 14.818 | 347.25832 | [M+H]+1 | 4 |
| Ftr521 | Myc_Var0000845 | C23 H29 N O6 | Xylactam C | 9.658 | 416.20703 | [M+H]+1 | 4 |
| Ftr521 | Myc_Var0000874 | C34 H57 N3 O6 | NA | 13.608 | 604.43237 | [M+H]+1 | 4 |
| Ftr521 | Myc_Var0000879 | C31 H52 N10 O5 | NA | 12.408 | 645.42219 | [M+H]+1 | 4 |

Table S3: Strain-specific exometabolites biomarkers identified through Upset plot. Each feature is described by: a producer (Strain_specific_feature), an identifier (**Variable_ID**); a retention time in minutes (**RT_min**); a mass-to-charge ratio associated with an adduct (**m/z_of_adduct** and **Adduct**); a putative molecular formula and name (**Putative_formula** and **Putative_name**) as assigned by Compound Discoverer software; and aconfidence level of identification (Schymanski et al., 2014).

| Strain_specific_ feature | Variable_ID | Putative_formula | Putative_name | RT_ min | m/z_of_adduct | Adduct | Confidence_level_of_ identification |
| --- | --- | --- | --- | --- | --- | --- | --- |
| Fav498 | Med_Var0000549 | C27 H32 O8 | JBIR-79 | 9.346 | 485.2173 | [M+H]+1 | 4 |
| Fav498 | Med_Var0000604 | C42 H62 N8 O9 | Anabaenopeptin SA3_iso2 | 8.182 | 412.23974 | [M+2H]+2 | 4 |
| Fav498 | Med_Var0000690 | C62 H125 N8 O19 P5 | NA | 5.108 | 481.26531 | [M+3H]+3 | 4 |
| Fcu337 | Med_Var0000146 | C17 H22 O8 | Fusarenon-X | 4.523 | 355.13896 | [M+H]+1 | 1 |
| Fcu337 | Med_Var0000195 | NA | NA | 6.229 | 416.12333 | [M+2H]+2 | 5 |
| Fcu337 | Med_Var0000206 | C50 H69 N8 O22 P3 | NA | 6.228 | 614.19412 | [M+2H]+2 | 4 |
| Fcu337 | Med_Var0000263 | C22 H27 N7 O4 P2 | NA | 4.522 | 258.58657 | [M+2H]+2 | 4 |
| Fcu337 | Med_Var0000271 | C39 H43 N6 O11 P | NA | 6.602 | 402.14382 | [M+2H]+2 | 4 |
| Fcu337 | Med_Var0000335 | C17 H18 O6 | Clavilactone E_iso1 | 6.227 | 319.11803 | [M+H]+1 | 4 |
| Fcu337 | Med_Var0000340 | C17 H20 O7 | (-)-applanatumol L | 6.227 | 337.12856 | [M+H]+1 | 4 |
| Fcu337 | Med_Var0000345 | C27 H24 O7 | BE-24566B_iso1 | 5.153 | 231.08367 | [M+2H]+2 | 4 |
| Fcu337 | Med_Var0000359 | C20 H26 O9 | Abyssomicin S | 7.057 | 433.1473 | [M+Na]+1 | 4 |
| Fcu337 | Med_Var0000395 | C19 H24 O9 | 4,15-Diacetylnivalenol | 6.227 | 397.14993 | [M+H]+1 | 4 |
| Fcu337 | Med_Var0000427 | NA | NA | 6.232 | 251.08986 | [M+2H]+2 | 5 |
| Fcu337 | Med_Var0000440 | C18 H22 N4 O3 | Polonimide C | 2.917 | 365.1574 | [M+Na]+1 | 4 |
| Fcu337 | Med_Var0000470 | C19 H24 O8 | 3,15-Acetyldeoxynivalenol_iso1 | 6.775 | 403.1367 | [M+Na]+1 | 3 |
| Fcu337 | Med_Var0000514 | C14 H14 O4 | Antrocinnamomin C | 4.522 | 247.09674 | [M+H]+1 | 4 |
| Fcu337 | Med_Var0000523 | C39 H50 N4 O7 S4 | NA | 6.229 | 408.13487 | [M+2H]+2 | 4 |
| Fcu337 | Med_Var0000527 | C22 H24 N6 O7 S | Penilumamide | 6.118 | 259.07859 | [M+2H]+2 | 4 |
| Fcu337 | Med_Var0000535 | C17 H24 O7 | Toxin NT-2_iso1 | 5.153 | 341.15989 | [M+H]+1 | 2a |
| Fcu337 | Med_Var0000540 | C22 H27 N5 O5 | NA | 6.241 | 442.20771 | [M+H]+1 | 4 |
| Fcu337 | Med_Var0000579 | C27 H63 N9 O3 P4 S2 | NA | 6.81 | 375.68031 | [M+2H]+2 | 4 |
| Fcu337 | Med_Var0000635 | C17 H18 O6 | Clavilactone E_iso2 | 4.523 | 319.11795 | [M+H]+1 | 4 |
| Fcu337 | Med_Var0000679 | C64 H113 N3 O26 | NA | 5.405 | 447.59489 | [M+3H]+3 | 4 |
| Fcu337 | Med_Var0000735 | C14 H14 O4 | 5-((2E,4E)-Hexa-2,4-dienoyl)-4-hydroxy-2-methoxybenzaldehyde | 6.227 | 247.09681 | [M+H]+1 | 4 |
| Fcu337 | Med_Var0000736 | C17 H22 O6 | (S)-7-hydroxytrichothecolone acetate_iso4 | 6.601 | 323.14926 | [M+H]+1 | 4 |
| Fcu337 | Med_Var0000795 | C15 H16 O5 | Conosilane A | 6.228 | 277.10739 | [M+H]+1 | 4 |
| Fcu337 | Med_Var0000826 | C21 H23 N4 O7 P | NA | 4.436 | 238.07329 | [M+2H]+2 | 4 |
| Fcu337 | Med_Var0000842 | C29 H26 O10 | Cercosporin | 6.233 | 268.08395 | [M+2H]+2 | 4 |
| Fcu337 | Med_Var0000846 | C8 H8 O | Phenylacetaldehyde_iso3 | 6.603 | 121.06509 | [M+H]+1 | 4 |
| Fcu337 | Med_Var0000850 | C15 H16 O5 | 5-hydroxy-4-oxo-2-(2-oxopropyl)-1,2,3,4-tetrahydronaphthalen-1-yl acetate | 4.522 | 277.10738 | [M+H]+1 | 4 |
| Fcu337 | Med_Var0000872 | C14 H12 O3 | 3,7-dihydroxy-1,9-dimethyldibenzofuran | 6.227 | 229.08625 | [M+H]+1 | 4 |
| Fcu337 | Med_Var0000875 | C15 H14 O4 | (S)-2,5-dihydroxy-2-methyl-1,2,3,4-tetrahydroanthracene-9,10-dione | 6.228 | 259.09671 | [M+H]+1 | 4 |
| Fcu337 | Med_Var0000918 | C19 H22 N4 O9 | Nikkomycin WX | 6.602 | 451.14548 | [M+H]+1 | 4 |
| Fcu337 | Med_Var0000922 | C30 H26 O9 | (-)-dalmanol C | 7.059 | 266.08648 | [M+2H]+2 | 4 |
| Fcu337 | Med_Var0000930 | C19 H37 N O5 | Curvularide C | 8.42 | 360.27492 | [M+H]+1 | 4 |
| Fcu337 | Med_Var0000970 | C12 H23 N O2 | NA | 5.72 | 214.18042 | [M+H]+1 | 4 |
| Fcu337 | Med_Var0000996 | C11 H23 N O | NA | 7.707 | 186.18552 | [M+H]+1 | 4 |
| Fcu337 | Med_Var0001043 | C14 H12 O | cis-Stilbeneoxide_iso4 | 6.625 | 197.09643 | [M+H]+1 | 4 |
| Fcu337 | Med_Var0001047 | C14 H14 O3 | Eucalyptene A_iso2 | 6.227 | 231.1019 | [M+H]+1 | 4 |
| Fcu337 | Med_Var0001050 | C14 H16 O3 | Sorbicillin_iso3 | 6.602 | 233.11754 | [M+H]+1 | 4 |
| Fcu337 | Med_Var0001052 | C9 H17 N | 2-Butyl-1-azacyclohexene iminium salt_iso3 | 7.094 | 172.16983 | [M+H+MeOH]+1 | 4 |
| Fcu337 | Med_Var0001065 | NA | NA | 6.227 | 201.09131 | [M+H-H2O]+1 | 5 |
| Fcu337 | Med_Var0001121 | C19 H15 N6 O5 P | NA | 4.525 | 439.09114 | [M+H]+1 | 4 |
| Fcu337 | Med_Var0001130 | C12 H25 N O3 | NA | 5.72 | 232.19105 | [M+H]+1 | 4 |
| Fcu337 | Med_Var0001151 | C29 H26 O8 | Demethoxyisogemichalcone C | 5.527 | 252.08905 | [M+2H]+2 | 4 |
| Fcu337 | Med_Var0001154 | NA | NA | 8.827 | strains | [M+H]+1 | 5 |
| Fcu337 | Med_Var0001159 | C19 H24 O8 | 3,15-Acetyldeoxynivalenol_iso3 | 6.183 | 403.13675 | [M+Na]+1 | 4 |
| Fcu337 | Med_Var0001168 | NA | NA | 9.001 | 334.19801 | [2M+H]+1 | 5 |
| Fgr149 | Med_Var0000079 | C36 H46 O7 P4 | NA | 5.554 | 358.11747 | [M+2H]+2 | 4 |
| Fgr149 | Med_Var0000081 | C51 H61 N2 O20 P | NA | 5.554 | 527.18574 | [M+2H]+2 | 4 |
| Fgr149 | Med_Var0000087 | C15 H20 O6 | Deoxynivalenol | 3.054 | 297.13346 | [M+H]+1 | 1 |
| Fgr149 | Med_Var0000124 | C19 H29 N3 O6 P2 | NA | 3.054 | 229.58386 | [M+2H]+2 | 4 |
| Fgr149 | Med_Var0000166 | NA | NA | 5.554 | 350.12888 | [M+2H]+2 | 5 |
| Fgr149 | Med_Var0000179 | C25 H22 O7 | Cycloartobiloxanthone | 3.054 | 218.0758 | [M+2H]+2 | 4 |
| Fgr149 | Med_Var0000186 | C27 H22 O7 | Naphthacemycin B1 | 5.553 | 230.07581 | [M+2H]+2 | 4 |
| Fgr149 | Med_Var0000285 | C10 H17 N O3 | N-Hexanoyl-L-homoserine lactone | 2.836 | 200.1284 | [M+H]+1 | 4 |
| Fgr149 | Med_Var0000297 | C20 H26 N6 O4 P2 | NA | 5.564 | 239.08112 | [M+2H]+2 | 4 |
| Fgr149 | Med_Var0000310 | C17 H22 O7 | 15-Acetyldeoxynivalenol | 5.554 | 339.14424 | [M+H]+1 | 1 |
| Fgr149 | Med_Var0000332 | C10 H18 O4 | Sebacic acid | 5.593 | 225.10997 | [M+Na]+1 | 4 |
| Fgr149 | Med_Var0000356 | C20 H31 N O6 S | NA | 6.789 | 414.19495 | [M+H]+1 | 4 |
| Fgr149 | Med_Var0000390 | NA | NA | 5.561 | 222.0871 | [M+2H]+2 | 5 |
| Fgr149 | Med_Var0000422 | C18 H19 N2 O8 P | NA | 5.553 | 464.12337 | [M+ACN+H]+1 | 4 |
| Fgr149 | Med_Var0000464 | C35 H73 N8 O12 P | NA | 8.167 | 829.51371 | [M+H]+1 | 4 |
| Fgr149 | Med_Var0000591 | C15 H22 N6 O2 | NA | 9.877 | 319.18818 | [M+H]+1 | 4 |
| Fgr149 | Med_Var0000606 | C15 H22 O | Anaephene A_iso8 | 9.223 | 219.17461 | [M+H]+1 | 3 |
| Fgr149 | Med_Var0000608 | C9 H16 O2 | 4,6-Nonanedione | 5.596 | 157.12251 | [M+H]+1 | 4 |
| Fgr149 | Med_Var0000634 | C37 H67 N6 O7 P3 S2 | NA | 7.927 | 865.3789 | [M+H]+1 | 4 |
| Fgr149 | Med_Var0000649 | C20 H27 N O4 S | NA | 6.79 | 378.17379 | [M+H]+1 | 4 |
| Fgr149 | Med_Var0000685 | C21 H39 N3 O13 | KA-5685 | 7.123 | 542.2536 | [M+H]+1 | 4 |
| Fgr149 | Med_Var0000728 | C15 H18 O5 | Hirsutenol D | 5.554 | 279.12302 | [M+H]+1 | 3 |
| Fgr149 | Med_Var0000777 | C20 H42 N O7 P3 S | NA | 6.789 | 267.60121 | [M+2H]+2 | 4 |
| Fgr149 | Med_Var0000836 | C17 H25 N O3 | Cyclizidine | 6.972 | 292.19108 | [M+H]+1 | 4 |
| Fgr149 | Med_Var0000857 | C19 H33 N O5 | NA | 6.17 | 356.24357 | [M+H]+1 | 4 |
| Fgr149 | Med_Var0000883 | C14 H14 O3 | Eucalyptene A_iso1 | 5.556 | 231.10183 | [M+H]+1 | 4 |
| Fgr149 | Med_Var0000903 | C21 H34 N4 O4 | Sclerotiotide B | 5.498 | 204.13656 | [M+2H]+2 | 3 |
| Fgr149 | Mebiomarker05 | C36 H62 N5 O13 P5 | NA | 5.555 | 464.65911 | [M+2H]+2 | 4 |
| Fgr149 | Med_Var0000943 | C9 H14 O | Isophorone | 4.778 | 139.11196 | [M+H]+1 | 4 |
| Fgr149 | Med_Var0000981 | C20 H40 N4 O5 | NA | 6.271 | 417.30755 | [M+H]+1 | 4 |
| Fgr149 | Med_Var0001001 | C14 H16 O3 | Creolophin C | 5.651 | 215.10695 | [M+H-H2O]+1 | 4 |
| Fgr149 | Med_Var0001002 | NA | NA | 4.383 | 275.12817 | [2M+Na]+1 | 5 |
| Fgr149 | Med_Var0001041 | C10 H14 O3 | (2,2,3-Trimethyl-5-oxocyclopent-3-en-1-yl)acetic acid | 3.875 | 183.10185 | [M+H]+1 | 4 |
| Fgr149 | Med_Var0001051 | C10 H14 O2 | 4-deoxyphomapyrone C | 4.778 | 167.10691 | [M+H]+1 | 4 |
| Fgr149 | Med_Var0001062 | C14 H14 O | NA | 4.383 | 199.11208 | [M+H]+1 | 4 |
| Fgr149 | Med_Var0001073 | C9 H21 N O5 S | NA | 5.32 | 256.12241 | [M+H]+1 | 4 |
| Fgr149 | Med_Var0001086 | C9 H17 N O2 | (E)-3-hydroxy-2,4-dimethylhept-4-enamide | 5.804 | 172.13343 | [M+H]+1 | 4 |
| Fgr149 | Med_Var0001097 | C14 H16 O4 | Illinitone B | 5.555 | 249.11246 | [M+H]+1 | 3 |
| Fgr149 | Med_Var0001100 | C12 H29 N6 O2 P S2 | NA | 4.382 | 193.08445 | [M+2H]+2 | 4 |
| Fgr149 | Med_Var0001103 | C7 H8 O | Anisole | 4.383 | 217.12267 | [2M+H]+1 | 4 |
| Fgr149 | Med_Var0001170 | NA | NA | 6.557 | 289.12039 | [M+H-H2O]+1 | 5 |
| Fla509 | Med_Var0000172 | NA | NA | 9.155 | 294.11772 | [M+2H]+2 | 5 |
| Fla509 | Med_Var0000269 | C12 H16 O3 | F-11334-B2 | 9.437 | 209.11749 | [M+H]+1 | 4 |
| Fpo073 | Med_Var0000351 | C47 H88 N9 O18 P | NA | 5.883 | 366.87359 | [M+3H]+3 | 4 |
| Fpo073 | Med_Var0000406 | C25 H51 N3 O2 | NA | 9.303 | 426.40579 | [M+H]+1 | 4 |
| Fpo073 | Med_Var0000439 | C46 H66 N8 O11 | Aselacin C | 5.177 | 454.24823 | [M+2H]+2 | 5 |
| Fsp101 | Med_Var0000085 | C17 H18 O4 | Trypethelon-methylether | 10.37 | 287.12801 | [M+H]+1 | 4 |
| Fsp101 | Med_Var0000091 | C13 H12 | NA | 9.153 | 169.10136 | [M+H]+1 | 4 |
| Fsp101 | Med_Var0000097 | C14 H14 O2 | 7-(1-hydroxyethyl)-4-methyl-1-azulenecarboxaldehyde_iso2 | 10.369 | 215.10688 | [M+H]+1 | 4 |
| Fsp101 | Med_Var0000098 | C19 H24 O7 | Neoabyssomicin A2 | 9.153 | 365.15987 | [M+H]+1 | 4 |
| Fsp101 | Med_Var0000099 | C19 H22 O6 | Gibberellin A3 | 10.369 | 347.14917 | [M+H]+1 | 4 |
| Fsp101 | Med_Var0000102 | C14 H12 O | cis-Stilbeneoxide_iso2 | 10.368 | 197.09627 | [M+H]+1 | 4 |
| Fsp101 | Med_Var0000108 | C21 H26 O8 | Lunatoic acid D | 10.369 | 407.17036 | [M+H]+1 | 4 |
| Fsp101 | Med_Var0000120 | C12 H10 O | NA | 9.153 | 203.10689 | [M+H+MeOH]+1 | 4 |
| Fsp101 | Med_Var0000121 | C16 H16 O3 | 7,4'-Dihydroxy-8-methylflavan_iso1 | 9.153 | 257.11747 | [M+H]+1 | 4 |
| Fsp101 | Med_Var0000125 | C28 H42 N2 O10 P2 | NA | 10.367 | 315.12294 | [M+2H]+2 | 4 |
| Fsp101 | Med_Var0000129 | C37 H35 N2 O2 P | NA | 9.153 | 286.12906 | [M+2H]+2 | 4 |
| Fsp101 | Med_Var0000130 | C17 H18 O4 | Trichorenin A | 9.153 | 287.12805 | [M+H]+1 | 4 |
| Fsp101 | Med_Var0000136 | NA | NA | 8.343 | 447.19922 | [M+H]+1 | 5 |
| Fsp101 | Med_Var0000145 | C24 H34 O8 | Maunakeanolin | 9.781 | 473.21466 | [M+Na]+1 | 4 |
| Fsp101 | Med_Var0000152 | C13 H14 O2 | Methyleutypinol_iso1 | 9.142 | 203.10693 | [M+H]+1 | 4 |
| Fsp101 | Med_Var0000160 | C52 H72 Cl2 O18 | Clostomicin C | 10.37 | 528.21217 | [M+2H]+2 | 5 |
| Fsp101 | Med_Var0000184 | C12 H10 | NA | 9.153 | 187.11196 | [M+H+MeOH]+1 | 4 |
| Fsp101 | Med_Var0000203 | C15 H14 O2 | 7-Hydroxyflavan_iso2 | 10.369 | 227.10686 | [M+H]+1 | 4 |
| Fsp101 | Med_Var0000231 | C14 H16 O3 | Sorbicillin_iso1 | 9.153 | 233.1175 | [M+H]+1 | 4 |
| Fsp101 | Med_Var0000238 | NA | NA | 9.156 | 486.20173 | [M+2H]+2 | 5 |
| Fsp101 | Med_Var0000240 | C15 H16 O3 | 7-(1,2-Dihydroxy-1-methylethyl)-4-methylazulene-1-carbaldehyde_iso1 | 10.37 | 245.11744 | [M+H]+1 | 4 |
| Fsp101 | Med_Var0000242 | C16 H16 O3 | 7,4'-Dihydroxy-8-methylflavan_iso2 | 10.369 | 257.11741 | [M+H]+1 | 4 |
| Fsp101 | Med_Var0000278 | C9 H17 N O3 | 8-Amino-7-oxononanoate | 5.022 | 188.12836 | [M+H]+1 | 4 |
| Fsp101 | Med_Var0000300 | C13 H12 O | NA | 10.369 | 185.0963 | [M+H]+1 | 4 |
| Fsp101 | Med_Var0000327 | C13 H12 | NA | 10.37 | 169.10135 | [M+H]+1 | 4 |
| Fsp101 | Med_Var0000331 | C27 H34 O9 | Vazabitide A_iso2 | 9.721 | 503.22564 | [M+H]+1 | 4 |
| Fsp101 | Med_Var0000353 | C27 H38 O10 | NA | 10.896 | 545.23613 | [M+Na]+1 | 4 |
| Fsp101 | Med_Var0000360 | C15 H18 O4 | Heterocornol O_iso2 | 8.047 | 263.12802 | [M+H]+1 | 4 |
| Fsp101 | Med_Var0000361 | C11 H8 | NA | 9.153 | 173.09631 | [M+H+MeOH]+1 | 4 |
| Fsp101 | Med_Var0000373 | C19 H24 O7 | Abyssomicin 4 | 10.369 | 365.15988 | [M+H]+1 | 4 |
| Fsp101 | Med_Var0000376 | C22 H32 O8 | HT-2 Toxin | 8.045 | 425.2174 | [M+H]+1 | 1 |
| Fsp101 | Med_Var0000382 | NA | NA | 8.348 | 215.10687 | [2M+Na]+1 | 5 |
| Fsp101 | Med_Var0000384 | C13 H14 | NA | 9.154 | 171.11704 | [M+H]+1 | 4 |
| Fsp101 | Med_Var0000403 | C17 H22 O6 | (S)-7-hydroxytrichothecolone acetate_iso1 | 9.154 | 323.14926 | [M+H]+1 | 4 |
| Fsp101 | Med_Var0000459 | C6 H10 N P | NA | 9.152 | 128.06227 | [M+H]+1 | 4 |
| Fsp101 | Med_Var0000462 | C11 H10 | NA | 9.153 | 143.08571 | [M+H]+1 | 4 |
| Fsp101 | Med_Var0000486 | C17 H22 O6 | (S)-7-hydroxytrichothecolone acetate_iso2 | 8.346 | 323.14916 | [M+H]+1 | 4 |
| Fsp101 | Med_Var0000488 | C16 H18 O4 | Collimonin C | 10.37 | 275.12807 | [M+H]+1 | 4 |
| Fsp101 | Med_Var0000490 | C12 H10 | NA | 10.369 | 187.11197 | [M+H+MeOH]+1 | 4 |
| Fsp101 | Med_Var0000504 | C21 H35 N O7 | NA | 5.809 | 414.24889 | [M+H]+1 | 4 |
| Fsp101 | Med_Var0000519 | C18 H22 O6 | Dihydroepiheveadride | 5.128 | 335.14921 | [M+H]+1 | 4 |
| Fsp101 | Med_Var0000520 | C24 H34 O9 | T-2 Toxin_iso1 | 9.33 | 467.22799 | [M+H]+1 | 3 |
| Fsp101 | Med_Var0000528 | C14 H14 O | NA | 10.37 | 199.11198 | [M+H]+1 | 4 |
| Fsp101 | Med_Var0000530 | C15 H16 O3 | 7-(1,2-Dihydroxy-1-methylethyl)-4-methylazulene-1-carbaldehyde_iso2 | 8.044 | 245.11744 | [M+H]+1 | 4 |
| Fsp101 | Med_Var0000573 | C26 H32 O10 | Sterenin I | 6.998 | 505.205 | [M+H]+1 | 4 |
| Fsp101 | Med_Var0000601 | C8 H12 N P | NA | 9.153 | 154.07788 | [M+H]+1 | 4 |
| Fsp101 | Med_Var0000615 | C14 H16 O3 | Sorbicillin_iso2 | 8.347 | 233.11747 | [M+H]+1 | 4 |
| Fsp101 | Med_Var0000616 | C26 H35 N5 O5 | NA | 9.724 | 498.27032 | [M+H]+1 | 4 |
| Fsp101 | Med_Var0000644 | C24 H30 O8 | Ramalinic acid B | 7.401 | 447.19931 | [M+H]+1 | 4 |
| Fsp101 | Med_Var0000650 | C55 H115 N9 O22 P2 | NA | 5.71 | 439.59486 | [M+3H]+3 | 4 |
| Fsp101 | Med_Var0000655 | C14 H14 O2 | 2,4,6,8,10,12-tetradeca-1,14-hexenedial_iso3 | 8.051 | 215.10688 | [M+H]+1 | 4 |
| Fsp101 | Med_Var0000698 | C27 H22 O6 | Bequinostatin D | 8.045 | 222.0784 | [M+2H]+2 | 4 |
| Fsp101 | Med_Var0000713 | C17 H20 O5 | PF1092B_iso2 | 10.369 | 305.13866 | [M+H]+1 | 4 |
| Fsp101 | Med_Var0000723 | C74 H132 N3 O17 P5 | NA | 4.682 | 497.61619 | [M+3H]+3 | 4 |
| Fsp101 | Med_Var0000739 | C26 H32 O9 | Chenopodolin B | 8.789 | 489.20995 | [M+H]+1 | 4 |
| Fsp101 | Med_Var0000746 | C38 H83 N4 O17 P | NA | 5.348 | 450.28215 | [M+2H]+2 | 4 |
| Fsp101 | Med_Var0000751 | C15 H18 O4 | Coniothyrinone B | 9.153 | 263.12805 | [M+H]+1 | 4 |
| Fsp101 | Med_Var0000757 | C17 H20 O5 | PF1092B_iso3 | 9.329 | 305.13861 | [M+H]+1 | 4 |
| Fsp101 | Med_Var0000769 | C15 H16 O2 | Variabilone_iso2 | 9.779 | 229.12256 | [M+H]+1 | 4 |
| Fsp101 | Med_Var0000772 | C7 H8 O2 | 3-Methylcatechol | 9.779 | 125.05994 | [M+H]+1 | 4 |
| Fsp101 | Med_Var0000787 | C19 H24 O6 | Gibberellin A1_iso3 | 9.778 | 349.16491 | [M+H]+1 | 4 |
| Fsp101 | Med_Var0000798 | C10 H10 N2 O2 | 2-(1-hydroxyethyl)-4(3H)quinazoline | 4.593 | 191.08178 | [M+H]+1 | 4 |
| Fsp101 | Med_Var0000802 | NA | NA | 9.52 | 215.10682 | [2M+Na]+1 | 5 |
| Fsp101 | Med_Var0000829 | C17 H26 O4 | Tricholopardin B_iso5 | 8.865 | 295.19065 | [M+H]+1 | 4 |
| Fsp101 | Med_Var0000843 | C13 H14 O2 | Methyleutypinol_iso2 | 8.347 | 203.10692 | [M+H]+1 | 4 |
| Fsp101 | Med_Var0000901 | C19 H28 O5 | Alterchothecene B_iso2 | 10.203 | 337.20129 | [M+H]+1 | 4 |
| Fsp101 | Med_Var0000916 | C14 H12 O | cis-Stilbeneoxide_iso3 | 8.348 | 197.09636 | [M+H]+1 | 4 |
| Fsp101 | Med_Var0000985 | C14 H14 O2 | 2,4,6,8,10,12-tetradeca-1,14-hexenedial_iso4 | 8.502 | 215.10689 | [M+H]+1 | 4 |
| Fsp101 | Med_Var0000993 | C17 H22 O5 | 13-hydroxy-15-methoxy-3-methyl-3,4,5,6,9,10-hexahydro-1H-benzo[c ][1]oxacyclododecine-1,7(8H)-dione | 10.45 | 289.1436 | [M+H-H2O]+1 | 4 |
| Fsp101 | Med_Var0001000 | C19 H28 O7 | Bellidisin A | 5.81 | 369.19115 | [M+H]+1 | 4 |
| Fsp101 | Med_Var0001017 | NA | NA | 7.626 | 431.16807 | [M+H]+1 | 5 |
| Fsp101 | Med_Var0001076 | C14 H14 O | NA | 5.133 | 199.11196 | [M+H]+1 | 4 |
| Fsp101 | Med_Var0001111 | C7 H8 O2 | Trichodenone A | 10.952 | 125.05996 | [M+H]+1 | 4 |
| Fsp101 | Med_Var0001113 | C17 H26 O4 | Cytosporone T_iso2 | 9.556 | 295.19067 | [M+H]+1 | 4 |
| Fsp101 | Med_Var0001134 | C16 H29 N3 O4 S | NA | 5.277 | 360.19573 | [M+H]+1 | 4 |
| Ftr521 | Med_Var0000114 | C22 H27 N O7 | Tirandamycin A_iso1 | 8.606 | 440.16806 | [M+Na]+1 | 4 |
| Ftr521 | Med_Var0000158 | C22 H27 N O7 | Tirandamycin A_iso2 | 8.826 | 440.1681 | [M+Na]+1 | 4 |
| Ftr521 | Med_Var0000227 | C22 H27 N O7 | Tirandamycin A_iso3 | 8.765 | 440.16814 | [M+Na]+1 | 4 |
| Ftr521 | Med_Var0000347 | C22 H27 N O7 | Tirandamycin A_iso4 | 8.933 | 418.18641 | [M+H]+1 | 4 |
| Ftr521 | Med_Var0000349 | C22 H27 N O7 | Tirandamycin A_iso5 | 9.01 | 418.18645 | [M+H]+1 | 4 |
| Ftr521 | Med_Var0000600 | C22 H27 N O5 | Streptoglyceride C | 9.767 | 386.19658 | [M+H]+1 | 4 |
| Ftr521 | Med_Var0000627 | C22 H25 N O6 | Cephalimysin A | 8.602 | 400.17588 | [M+H]+1 | 4 |
| Ftr521 | Med_Var0000915 | C14 H14 O | NA | 8.603 | 199.11202 | [M+H]+1 | 4 |
| Ftr521 | Med_Var0000927 | C14 H14 O | NA | 8.829 | 199.11205 | [M+H]+1 | 4 |
| Ftr521 | Med_Var0000954 | C8 H6 O | NA | 3.199 | 136.07593 | [M+NH4]+1 | 4 |
| Ftr521 | Med_Var0001123 | C11 H13 N O3 | N-Acetyl-L-phenylalanine | 5.288 | 208.09717 | [M+H]+1 | 4 |
| Ftr521 | Med_Var0001128 | NA | NA | 2.685 | 256.09717 | [M+ACN+H]+1 | 5 |
| Ftr521 | Med_Var0001139 | C15 H29 N3 O4 | NA | 4.926 | 316.22354 | [M+H]+1 | 4 |
| Ftr521 | Med_Var0001142 | C9 H11 N O2 | L-Phenylalanine_iso2 | 5.288 | 166.08657 | [M+H]+1 | 4 |

Table S4: Strain-specific endometabolites biomarkers identified in Fusarium co-cultures. Each feature is described by: a producer (Strain_specific_feature), two identifier (**Co-culture_Variable_ID and Single-strain_Varaible_ID**); a retention time in minutes (**RT_min**); a mass-to-charge ratio associated with an adduct (**m/z_of_adduct** and **Adduct**); a putative molecular formula and name (**Putative_formula** and **Putative_name**) as assigned by Compound Discoverer software; and a confidence level of identification (Schymanski et al., 2014).

| Strain_specific_feature | *Co-culture_* Variable_ID | Single-strain_ Variable_ID | Putative_formula | Putative_name | RT_min | m/z_of_adduct | Adduct | Confidence_level_of_identification |
| --- | --- | --- | --- | --- | --- | --- | --- | --- |
| Fcu337 | MF_St_Cond_Myc_Var000038 | Myc_Var0000786 | C19 H24 O9 | 4,15-Diacetylnivalenol | 6.147 | 419.13061 | [M+Na]+1 | 4 |
| Fcu337 | MF_St_Cond_Myc_Var000092 | Myc_Var0000233 | C29 H24 O9 | Aschernaphthopyrone A | 6.147 | 259.07814 | [M+2H]+2 | 4 |
| Fgr149 | MF_St_Cond_Myc_Var000095 | Myc_Var0000888 | C12 H23 N O4 | 3-hydroxy-N-(1-hydroxy-3-methylpentan-2-yl)-5-oxohexanamide_iso1 | 5.64 | 228.15926 | [M+H-H2O]+1 | 4 |
| Fcu337 | MF_St_Cond_Myc_Var000220 | Myc_Var0000397 | NA | NA | 6.147 | 416.12255 | [M+2H]+2 | 5 |
| Fcu337 | MF_St_Cond_Myc_Var000238 | Myc_Var0000363 | C21 H41 N O2 | NA | 13.958 | 340.3208 | [M+H]+1 | 4 |
| Fcu337 | MF_St_Cond_Myc_Var000255 | Myc_Var0000863 | C14 H14 O2 | 2,4,6,8,10,12-tetradeca-1,14-hexenedial_iso1 | 13.405 | 247.13271 | [M+H+MeOH]+1 | 4 |
| Fcu337 | MF_St_Cond_Myc_Var000262 | Myc_Var0000513 | C26 H32 N2 O2 S3 | NA | 6.146 | 251.08946 | [M+2H]+2 | 4 |
| Fcu337 | MF_St_Cond_Myc_Var000287 | Myc_Var0000639 | C22 H45 N O2 | ArachidoylEthanolamide | 15.285 | 356.35204 | [M+H]+1 | 4 |
| Fcu337 | MF_St_Cond_Myc_Var000292 | Myc_Var0000317 | C19 H26 O8 | 7,8-Dihydroxycalonectrin | 6.515 | 383.16974 | [M+H]+1 | 3 |
| Fgr149 | MF_St_Cond_Myc_Var000308 | Myc_Var0000215 | C27 H22 O7 | Naphthacemycin B1 | 5.481 | 230.0755 | [M+2H]+2 | 4 |
| Fcu337 | MF_St_Cond_Myc_Var000320 | Myc_Var0000401 | C17 H22 O8 | Fusarenone-X | 4.449 | 377.12039 | [M+Na]+1 | 1 |
| Fcu337 | MF_St_Cond_Myc_Var000360 | Myc_Var0000911 | C15 H16 O5 | Conosilane A | 6.147 | 277.10697 | [M+H]+1 | 4 |

Table S5: Strain-specific exometabolites biomarkers identified in Fusarium co-cultures. Each feature is described by: a producer (Strain_specific_feature), two identifier (**Co-culture_Variable_ID and Single-strain_Varaible_ID**); a retention time in minutes (**RT_min**); a mass-to-charge ratio associated with an adduct (**m/z_of_adduct** and **Adduct**); a putative molecular formula and name (**Putative_formula** and **Putative_name**) as assigned by Compound Discoverer software; and a confidence level of identification (Schymanski et al., 2014).

| Strain_specific_feature | *Co-culture_* Variable_ID | Single-strain_ Variable_ID | Putative_formula | Putative_name | RT_min | m/z_of_adduct | Adduct | Confidence_level_of_identification |
| --- | --- | --- | --- | --- | --- | --- | --- | --- |
| Fcu337 | MF_St_Cond_Med_Var000063 | Med_Var0000206 | C52 H74 N6 O18 P4 S | NA | 6.147 | 614.1935 | [M+2H]+2 | 4 |
| Fcu337 | MF_St_Cond_Med_Var000070 | Med_Var0000195 | NA | NA | 6.147 | 416.12284 | [M+2H]+2 | 5 |
| Fcu337 | MF_St_Cond_Med_Var000115 | Med_Var0000146 | C17 H22 O8 | Fusarenone-X | 4.45 | 355.13881 | [M+H]+1 | 1 |
| Fcu337 | MF_St_Cond_Med_Var000132 | Med_Var0000263 | C24 H22 N9 O3 P | NA | 4.45 | 258.58645 | [M+2H]+2 | 4 |
| Fcu337 | MF_St_Cond_Med_Var000135 | Med_Var0001151 | C29 H26 O8 | Demethoxyisogemichalcone C | 6.512 | 252.08875 | [M+2H]+2 | 4 |
| Fcu337 | MF_St_Cond_Med_Var000145 | Med_Var0000271 | C39 H43 N6 O11 P | NA | 6.513 | 402.14357 | [M+2H]+2 | 4 |
| Fcu337 | MF_St_Cond_Med_Var000196 | Med_Var0000427 | C23 H28 N6 O3 S2 | NA | 6.152 | 251.0897 | [M+2H]+2 | 4 |
| Fcu337 | MF_St_Cond_Med_Var000229 | Med_Var0000826 | C23 H30 N2 O P4 | NA | 4.365 | 238.07316 | [M+2H]+2 | 4 |
| Fcu337 | MF_St_Cond_Med_Var000230 | Med_Var0000523 | C50 H42 N2 O5 S2 | NA | 6.147 | 408.13437 | [M+2H]+2 | 4 |
| Fcu337 | MF_St_Cond_Med_Var000235 | Med_Var0000340 | C17 H20 O7 | (-)-applanatumol L | 6.147 | 337.1282 | [M+H]+1 | 4 |
| Fcu337 | MF_St_Cond_Med_Var000240 | Med_Var0000395 | C19 H24 O9 | 4,15-Diacetylnivalenol | 6.145 | 397.14952 | [M+H]+1 | 4 |
| Fcu337 | MF_St_Cond_Med_Var000331 | Med_Var0000345 | C27 H24 O7 | BE-24566B_iso2 | 5.071 | 231.08357 | [M+2H]+2 | 4 |
| Fcu337 | MF_St_Cond_Med_Var000335 | Med_Var0000527 | C22 H24 N6 O7 S | Penilumamide | 6.034 | 259.07848 | [M+2H]+2 | 4 |
| Fcu337 | MF_St_Cond_Med_Var000370 | Med_Var0000535 | C17 H24 O7 | Toxin NT-2_iso1 | 5.071 | 341.15964 | [M+H]+1 | 2a |
| Fcu337 | MF_St_Cond_Med_Var000371 | Med_Var0000514 | C14 H14 O4 | Coniochaetone E | 4.451 | 247.0966 | [M+H]+1 | 4 |
| Fcu337 | MF_St_Cond_Med_Var000407 | Med_Var0000795 | C15 H16 O5 | Conosilane A | 6.147 | 277.10718 | [M+H]+1 | 4 |
| Fcu337 | MF_St_Cond_Med_Var000413 | Med_Var0000875 | C15 H14 O4 | (S)-2,5-dihydroxy-2-methyl-1,2,3,4-tetrahydroanthracene-9,10-dione | 6.148 | 259.09642 | [M+H]+1 | 4 |
| Fgr149 | MF_St_Cond_Med_Var000127 | Med_Var0000186 | C27 H22 O7 | Naphthacemycin B1 | 5.481 | 230.07568 | [M+2H]+2 | 4 |
| Fgr149 | MF_St_Cond_Med_Var000161 | Med_Var0000079 | C32 H44 O14 P2 | NA | 5.482 | 358.11739 | [M+2H]+2 | 4 |
| Fgr149 | MF_St_Cond_Med_Var000266 | Med_Var0000297 | C19 H30 N2 O8 P2 | NA | 5.484 | 239.08101 | [M+2H]+2 | 4 |
| Fgr149 | MF_St_Cond_Med_Var000299 | Med_Var0000728 | C15 H18 O5 | Hirsutenol D | 5.483 | 261.11226 | [M+H-H2O]+1 | 3 |
| Fgr149 | MF_St_Cond_Med_Var000317 | Med_Var0000390 | C20 H30 N2 O5 S2 | Bilain B | 5.482 | 222.08698 | [M+2H]+2 | 4 |
| Fgr149 | MF_St_Cond_Med_Var000358 | Med_Var0000166 | C32 H42 O17 | Dihydrophelloside | 5.482 | 350.12875 | [M+2H]+2 | 4 |
| Fgr149 | MF_St_Cond_Med_Var000379 | Med_Var0000857 | C19 H33 N O5 | NA | 5.971 | 356.2433 | [M+H]+1 | 4 |
| Fgr149 | MF_St_Cond_Med_Var000393 | Med_Var0000081 | C49 H66 O21 P2 | NA | 5.482 | 527.18583 | [M+2H]+2 | 4 |
